# Associations between parental perceptions of neighbourhood environment and physical activity in children and adolescents: a systematic review including 149 studies

**DOI:** 10.1186/s12966-025-01733-8

**Published:** 2025-06-06

**Authors:** Ruirui Xing, Jerome N. Rachele, Tena Matolic, Venurs Loh, Ester Cerin, Jiao Jiao, Wendy Yajun Huang, Zeljko Pedisic

**Affiliations:** 1 Institute for Health and Sport, Victoria University, Melbourne, Australia; 2 College of Sport, Health and Engineering, Victoria University, Melbourne, Australia; 3 Faculty of Kinesiology, University of Zagreb, Zagreb, Croatia; 4 Mary MacKillop Institute for Health Research, Australian Catholic University, Melbourne, Australia; 5 School of Public Health, Li Ka Shing Faculty of Medicine, The University of Hong Kong, Hong Kong, China; 6 Academy of Wellness and Human Development, Hong Kong Baptist University, Hong Kong, China

**Keywords:** Built environment, Social environment, Crime, Personal safety, School proximity, Walkability, Walking, Cycling, Independent mobility, Outdoor play

## Abstract

**Background:**

Parental perceptions of the neighbourhood environment can be of particular importance for physical activity of children and adolescents, because parents act as the gatekeepers of their children’s behaviour. However, knowledge gaps remain regarding the associations between parental perceptions of neighbourhood environment and physical activity among children and adolescents. Therefore, the aim of this study was to systematically review and summarise evidence on the association between parental perceptions of the neighbourhood environment and physical activity among children and adolescents (5 – 17 years of age).

**Methods:**

Literature searches were conducted in: CINAHL, Embase, Environmental Science, MEDLINE/PubMed, PsycInfo, Scopus, SPORTDiscus, Transportation Research Information Services, and Web of Science. The associations were coded as: mostly favourable (for 60% – 100% of studies showing a positive association); mostly unfavourable (for 60% – 100% of studies showing a negative association); and mostly non-significant, indeterminate, or inconsistent.

**Results:**

Out of 30,162 records identified in the search, 162 papers from 149 studies were included in the review. The most consistent finding was that a greater distance to school is unfavourably associated with active travel. Evidence of this association was found in children (5/7 associations; pooled sample size in the studies showing significant association [*n*] = 14,113), adolescents (3/4; *n* = 2328), and mixed-age group (8/13; *n* = 5410). There was some consistency in evidence on favourable associations of: (1) access to public transport, good street lighting, and presence of crossing guards with active travel among children; (2) access to sports and recreational facilities, parks and/or playgrounds with sports participation among children; and (3) access to sports and recreational facilities, parks, and/or playgrounds with non-type-specific physical activity among adolescents. Several associations were found in individual studies only, while others were mostly non-significant, indeterminate, or inconsistent. The quality of evidence ranged from very low to low.

**Conclusions:**

Parental perceptions of traffic safety and access to destinations and services are associated with different types of physical activity among children and adolescents. There is a need for longitudinal and experimental studies, more research among adolescents, more studies from low- and middle-income countries, and exploring a wider range of neighbourhood environment attributes.

**Supplementary Information:**

The online version contains supplementary material available at 10.1186/s12966-025-01733-8.

## Background

The benefits of physical activity for the health and well-being of children and adolescents are well established [1, 2]. The World Health Organization (WHO) recommends that children and adolescents aged 5–17 years should accumulate at least 60 min of moderate-to-vigorous physical activity per day [2]. However, a recent Global Matrix 4.0 Report Card found that on average only 27–33% of children and adolescents from 54 countries accumulated the recommended amount of moderate-to-vigorous physical activity [3]. Evidence suggests that a lack of physical activity may have long-term health implications that carry over into adulthood, including increased risk of obesity, metabolic syndrome, poor metal health, and low quality of life [4–6]. Therefore, it is imperative to achieve and maintain adequate levels of physical activity during childhood and adolescence to effectively mitigate these health risks.

Various characteristics of the neighbourhood environment are associated with physical activity among children and adolescents [7, 8], for example, walking and cycling infrastructure [9], street connectivity [10] and greenery and aesthetics [11]. Such characteristics can be assessed subjectively (i.e. as perceived by study participants) and/or objectively (e.g. using Geographic Information Systems [GIS]). A previous review found that both subjective and objective measures of the neighbourhood environment are associated with physical activity among children and adolescents [12].

Parental perceptions have been identified as a subjective measure of the neighbourhood environment that is of particular importance for physical activity of children and adolescents, because parents act as the gatekeepers of their children’s behaviour [13]. Interestingly, children’s active travel to school was found to be more strongly associated with parental perceptions of neighbourhood safety and traffic safety than with objective measures of the neighbourhood environment [14].

Several previous reviews have synthesised evidence on the associations between features of the neighbourhood environment and physical activity among children and adolescents [7, 15–19]. However, knowledge gaps remain regarding the associations between parental perceptions of neighbourhood environment and physical activity among children and adolescents. First, several reviews focused only on specific types of physical activity, such as active travel [7, 15, 16] and outdoor play [17, 18]. Second, although Ding and colleagues [12] and Timperio and colleagues [8] explored various activity types, their reviews included papers published before 2010 and 2015, respectively. Third, a more recent review did not make a distinction between characteristics of the neighbourhood environment reported by children and parents [7]; thus, lacking specific conclusions about the associations between parental perceptions of neighbourhood environment and physical activity among children and adolescents. In addition, their literature search was conducted in 2018, and a number of new papers have since been published [7, 15, 16].

The United Nations International Children's Emergency Fund’s (UNICEF) handbook on child-responsive urban planning highlights how road safety policies have focused on raising awareness of road dangers among children and families [20]. While such policies have reduced road casualties, some of them may have also restricted children’s independent mobility, giving children less freedom to walk, cycle and play in their neighbourhood without adult supervision [20, 21]. Similarly, the Global Designing Cities Initiative and National Association of City Transportation Officials emphasise the importance of tailoring street design to the needs of children and their caregivers [22]. An up-to-date summary of evidence on the association between parental perceptions of neighbourhood environment and physical activity among children and adolescents is needed to inform the development and refinement of neighbourhood design policies and initiatives.

Therefore, the aim of this study was to systematically review and summarise evidence on the association between parental perceptions of the neighbourhood environment and physical activity among children and adolescents.

## Methods

The study was registered in the International Prospective Register of Systematic Reviews (PROSPERO) under the identification code CRD42023379968. The review was written according to the Preferred Reporting Items for Systematic Reviews and Meta-Analyses (PRISMA) statement [23]. Deviations from the registered protocol are described in Additional file 1.

### Search strategy

Literature searches were conducted in December 2023 in the following bibliographic databases: CINAHL, Embase, Environmental Science, MEDLINE/PubMed, PsycInfo, Scopus, SPORTDiscus, Transportation Research Information Services (TRIS), and Web of Science Core Collection. CINAHL, PsycInfo, and SPORTDiscus databases were searched through EBSCOhost, and Environment Science was searched through ProQuest. We searched for documents including terms related to parents, characteristics of the neighbourhood environment, physical activity, perceptions, and children and adolescents in their titles, abstracts, and/or keywords. The search syntax is provided in Additional file 2. Backward citation tracking was performed to identify any relevant documents cited in the included papers. We also searched for any additional relevant documents through Active Living Research, Clinical Excellence Queensland, Heart Foundation, National Institutes of Health (the United States), Open Grey, and Sustrans websites and reference lists of previous reviews on the association between neighbourhood environment and physical activity among children and adolescents.

### Inclusion criteria and study selection process

Studies meeting the following criteria were included in this review: (1) conducted among children and/or adolescents (5 – 17 years of age) selected from a non-clinical population; (2) analysed associations between parental perceptions of the neighbourhood environment as explanatory variables and any type of physical activity (except physical activity at school) as the outcome variable; and (3) published in Chinese or English. Commentaries, editorials, conference abstracts, literature reviews and qualitative studies were excluded. Study selection was undertaken by two authors independently (RX and TM for publications in English and JJ and RX for publications in Chinese). Disagreements were resolved through discussion between the two authors and, when needed, by another author (JNR). The study selection was performed in Covidence [24].

### Data extraction

Data were extracted independently by two authors (JJ and RX). Disagreements were discussed between the two authors. If consensus could not be reached, another author (JNR) was consulted. The following data were extracted: surname of the first author, publication year, country/region in which data were collected, study design, project name, response rate, sample type, sample size, age group, measures of parental perceptions of the neighbourhood environment, function of parental perceptions in relation to the outcome variable (e.g. correlates, mediators), measures of physical activity (e.g. device-measured, self-reports or proxy reports), data analysis method, adjustments for confounding, and key findings.

Two authors (RX and ZP) classified the neighbourhood environment attributes examined in the selected studies. The classification included eight constructs measured by the Neighbourhood Environment Walkability Scale (NEWS) [25], environmental hazards, social environment factors, and cross-category scores. The classification included 28 neighbourhood environment attributes in the following categories: (1) combined scores (for variables that represent attributes belonging to two or more of the remaining categories); (2) access to destinations and services; (3) physical barriers; (4) walking and cycling infrastructure; (5) greenery and aesthetics; (6) street connectivity; (7) residential density; (8) crime/personal safety; (9) traffic safety; (10) environmental hazards; and (11) social environment (Table 1). The classification was based on the NEWS or data driven in cases when neighbourhood environment attributes presented in the included studies could not be fitted into any NEWS category.

**Table 1:** Categorisation of neighbourhood environment attributes

Category	Neighbourhood environment attribute	Description and/or examples
Combined scores	General activity friendliness	Individual item about overall neighbourhood activity friendliness (e.g. “How pleasant is it to walk, run, bike, or play in your neighborhood?” [26]) or combined score calculated from items belonging to five or more categories
	General safety	Individual item about safety in general (e.g. “This is a safe neighbourhood.” [27]) or combined score calculated from items belonging to both traffic safety and crime/personal safety
	Other cross-category scores	Combined score representing two to four categories (e.g. the “Walking Infrastructure” factor representing the following two items: “There are not enough sidewalks” and “There are major barriers/obstacles to walking in my local neighbourhood that make it hard to get from place to place.” [28])
Access to destinations and services	Access to public transport	e.g. “It is easy to walk to a transit stop (bus, train) from my home.” [29]
	Access to shopping places and food outlets	e.g. “Stores are within easy walking distance of my home.” [29]
	Access to sports and recreational facilities, parks, and/or playgrounds	e.g. “There are few sporting venues within our local area.” [30]
	Availability of parking	e.g. “Parking is difficult in local shopping areas.” [29]
	Distance to school	e.g. “There is a long distance from home to school.” [31]
	Land use mix / destination mix score	Individual item asking about access to destinations/services in general (e.g. “There are many places to go within easy walking distance of my home.” [29]) or combined score calculated from items referring to two or more destinations/services
Physical barriers	General physical barriers score	A combined score calculated from items referring to hilliness and major physical barriers limiting the number of routes
	Hilliness	e.g. “The streets in my neighborhood are hilly, making my neighborhood difficult to walk in.” [29]
	Major physical barriers limiting the number of routes	e.g. “There are major barriers to walking/cycling in my local neighbourhood that make it hard for my child to get from place to place (e.g. freeways, major roads).” [32]
Walking and cycling infrastructure	Availability of walking and/or cycling infrastructure	e.g. “There are footpaths on most streets in our local neighborhood.” [33]
	General walking and/or cycling infrastructure score	Individual item about availability and quality of walking and/or cycling infrastructure (e.g. “There are no bicycle lanes or they are in poor conditions.” [31]) or combined score calculated from items referring to availability and quality of walking and/or cycling infrastructure
	Quality of walking and/or cycling infrastructure	e.g. “The sidewalks in my neighborhood are well maintained.” [29]
Greenery and aesthetics	More greenery and/or better aesthetics	e.g. “There are trees along the streets in my neighborhood.” [29]
Street connectivity	Street connectivity	e.g. “There are many shortcuts for walking in my neighbourhood.” [34]
Residential density	Residential density	e.g. “How common are detached single-family residences in your immediate neighborhood?” [29]
Crime/personal safety	General crime/personal safety	e.g. “I fear that my child would become a victim of violence or harassment near home.” [35]
Traffic safety	Availability of pedestrian crossings and signals	e.g. “There are no lights/crossings for my child to use.” [30]
	Busy/dangerous intersections and crossings	e.g. “There are no dangerous crossings.” [36]
	General traffic safety	Individual item about traffic safety in general (e.g. “I am concerned my child will be hurt in a traffic accident on the way to and/or from school.” [31]) or combined score calculated from items referring to different aspects of traffic safety
	Good street lighting	e.g. “My neighborhood streets are well lit at night.” [29]
	Number of roads to cross en route	e.g. “There are too many roads to cross for my child to walk to and/or from school.” [37]
	Presence of crossing guards	e.g. “Concerns about manned crossings.” [38]
	Traffic volume and/or speed	e.g. “There is heavy traffic in our local streets.” [32]
Environmental hazards	High air pollution	e.g. “When walking in my neighborhood, there are a lot of exhaust fumes (such as from cars, buses).” [29]
Social environment	Physical activity of others in the neighbourhood	e.g. “I see many people being physically active in my neighborhood.” [39]
	Social capital and/or cohesion	e.g. “This is a close-knit neighbourhood.” [40]
	Social disorder	e.g. “How much of a problem to you are any of the following in your neighbourhood: (1) beggars and addicts, (2) groups causing trouble, (3) reckless neighbours?” [41]

Findings from the included studies were extracted separately for the following outcome variables: active travel; non-type-specific physical activity; active independent mobility; sports participation; and active outdoor play, similar as in a large international study among children and adolescents [3].

### Data coding and synthesis

The associations between parental perceptions of the neighbourhood environment and physical activity reported in the included studies were categorized as favourable (i.e. positive), unfavourable (i.e. negative), and mixed, inconsistent or non-significant. If an included study reported more than one result for a single association (e.g. separate results obtained using different analytical approaches or for various variables measuring the same neighbourhood environment attribute), the association was coded as: “ + ” or mostly favourable (for 60% – 100% of results showing a positive association); “-” or mostly unfavourable (for 60% – 100% of results showing a negative association); and “?” or mixed (i.e. a mix of favourable and unfavourable associations), inconsistent (i.e. a mix of significant and non-significant associations) or non-significant. When there were two or more papers from the same study, their findings were combined. Findings from all studies that reported a given association (e.g. between distance to school and active travel) were then summarised using the procedure from a previous study [42], which is an adaptation of the method proposed by Sallis and colleagues [43]. According to the procedure, the summary results were coded as: “ + ” or mostly favourable (for 60% – 100% of studies showing a positive association); “-” or mostly unfavourable (for 60% – 100% of studies showing a negative association); and “?” or mostly non-significant, indeterminate, or inconsistent. For favourable, unfavourable and non-significant associations reported in four or more studies, we used summary codes “ + + ”, “--”, and “??”, respectively.

### Methodological quality assessment

One author (RX) assessed the methodological quality of included papers using a scale proposed by Cerin and colleagues [44–48], as in previous neighbourhood environment research [49, 50]. In case of any doubts about the quality assessment, two other authors (JNR and VL) were consulted. The scale has eleven items referring to: (1) study design (cross-sectional = 0 points, longitudinal = 1 point, experimental = 2 points); (2) sample size (< 100 = 0 points, 100–299 = 1/2 points, ≥ 300 = 1 point); (3) study areas or participant recruitment stratified by key environmental attributes (yes = 1 point, no = 0 points); (4) response rate (< 60% or sample representative of the population = 1 point, ≥ 60% at follow-up = 2 points); (5) parental perceptions of neighbourhood environment measures shown to be valid and reliable (yes = 1 point, no = 0 points); (6) physical activity outcome measures shown to be valid and reliable (yes = 1 point, no = 0 points); (7) adjustment for key socio-demographic characteristics, that is, age, sex and education (yes = 1 point, no = 0 points); (8) adjustment for self-selection into neighbourhoods (yes = 1 point, no = 0 points); (9) analytical approach accounted for area-level clustering (yes = 1/3 points, no = 0 points); (10) analytical approach accounted for distributional assumptions (yes = 1/3 points, no = 0 points); and (11) analyses conducted and presented correctly, including the calculation of effect sizes and their statistical significance, standard errors, or confidence intervals (yes = 1/3 points, no = 0 points). The overall score was calculated as the sum of scores for each item and categorised as “low” (0–5.5 points), “moderate” (5.6–8.5 points), and “high” (8.6–11 points).

### Quality of evidence assessment

The quality of evidence assessment was performed independently by two authors (RX and ZP), according to the Grading of Recommendations, Assessment, Development and Evaluation (GRADE) criteria [51], and categorised as “very low”, “low”, “moderate”, and “high”. The quality of evidence coming mostly from observational studies and experimental studies was initially rated as “low” and “high”, respectively. We then considered the following reasons for downgrading the quality of evidence: (1) risk of bias; (2) inconsistency of results; (3) indirectness of evidence; (4) imprecision; and (5) publication bias. Given the nature of evidence synthesis conducted in this review, none of the GRADE indications for upgrading the quality of evidence were applicable to our assessment. More details about the quality of evidence assessment can be found in Additional file 3.

## Results

### Literature search results

**Fig. 1:**
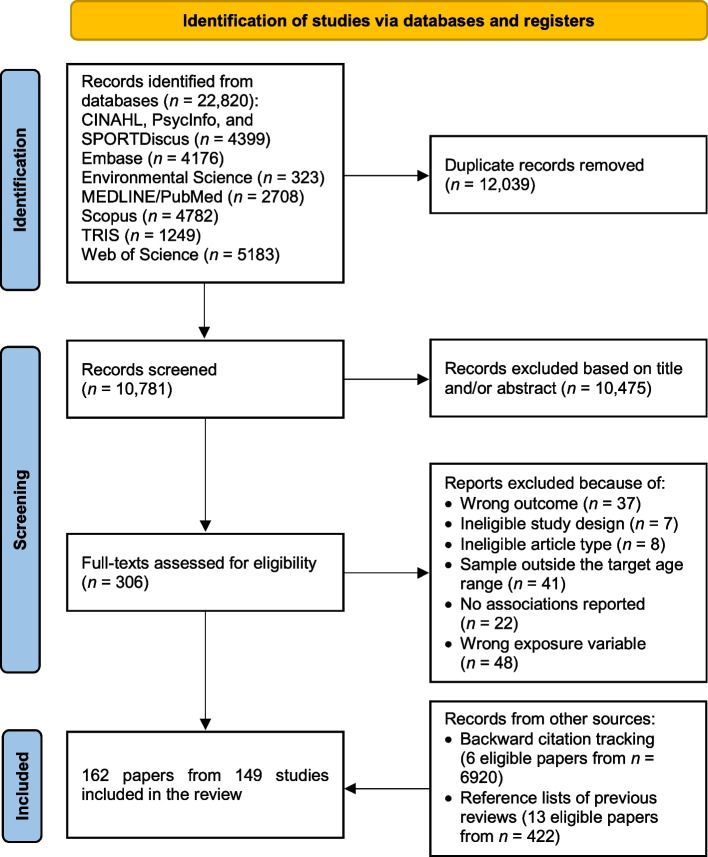
Flow diagram of the search and study selection process

After excluding duplicates from the 22,820 records identified in the search through bibliographic databases, we screened titles and abstracts of 10,781 unique records (Fig. 1). From 306 full-texts that we assessed, 143 met the inclusion criteria. Additional 19 papers meeting the inclusion criteria were identified via backward citation tracking and in reference lists of previous systematic reviews, and a total of 162 papers [9–11, 14, 27, 28, 30–33, 35–38, 41, 52–198] from 149 studies were included in the review.

### Characteristics of included papers

The vast majority of included papers (79.0%) were published post-2010 (Additional file 4). Approximately one-third of the papers (37.3%) originated from the United States, while 14.3% were from Australia (Table 2). The sample sizes ranged from 52 to 68,288, with the response rates from 8.0% to 95.2%. The child samples included only children in 32.7%, only adolescents in 14.8%, and both age groups in 52.5% of the included papers. Two studies included only female participants, while the remaining studies included both sexes. The parent samples included both parents in 1.2%, only mothers in 4.3%, mostly mothers in 17.9%, and mostly fathers in 0.6% of the included papers. Most of the included papers did not report the distribution of sexes in the parent sample.

**Table 2:** Summary characteristics of included papers

**Characteristic**	**No.**^a^	**%**
*Study design*		
Cross-sectional	147	90.7
Longitudinal	14	8.6
Experimental	1	0.6
*Country*		
United States	60	37.3
Australia	23	14.3
Canada	15	9.3
Belgium	10	6.2
UK	6	3.7
New Zealand	5	3.1
Iran	4	2.5
China	3	1.9
Germany	3	1.9
Spain	3	1.9
Switzerland	3	1.9
Hong Kong	2	1.2
Portugal	2	1.2
Republic of Ireland	2	1.2
Albania	1	0.6
Argentina	1	0.6
Austria	1	0.6
Brazil	1	0.6
Cyprus	1	0.6
Ecuador	1	0.6
Ghana	1	0.6
India	1	0.6
Japan	1	0.6
Kenya	1	0.6
Lithuania	1	0.6
Malaysia	1	0.6
Netherlands	1	0.6
Norway	1	0.6
Slovenia	1	0.6
South Africa	1	0.6
Sweden	1	0.6
Turkey	1	0.6
Uganda	1	0.6
12 countries	1	0.6
*Sample size*		
≥ 1001	59	36.4
501–1000	45	27.8
301–500	27	16.7
101–300	28	17.3
≤ 100	3	1.9
*Research project*		
Teen Environment and Neighborhood (TEAN)	6	3.7
TRavel Environment and Kids (TREK)	6	3.7
Safe Routes to School (SRTS) program	5	3.1
Built Environment and Active Transportation Research Project (BEAT)	4	2.5
Children Living in Active Neighbourhoods (CLAN)	4	2.5
Early Childhood Longitudinal Study—Kindergarten Cohort 1998–1999 (ECSL-K)	4	2.5
National Survey of Children's Health (NSCH)	4	2.5
Neighborhood Impact on Kids (NIK)	4	2.5
International Study of Childhood Obesity, Lifestyle and Environment (ISCOLE)	3	1.9
International Physical Activity and the Environment Network (IPEN)	3	1.9
National Household Travel Survey (NHTS)	3	1.9
Pedalea y Anda al COlegio (PACO)	3	1.9
Sport, Physical activity and Eating behaviour: Environmental Determinants in Young People (SPEEDY)	3	1.9
Resilience for Eating and Activity Despite Inequality (READI) and the Active Independent Mobility (AIM)	3	1.9
Built Environment and Active Play (BEAP)	2	1.2
Belgian Environmental Physical Activity Study in Children (BEPAS-child)	2	1.2
Active Transportation (AT) and Independent Mobility (IM) study	2	1.2
Growing Up in Ireland (GUI)	2	1.2
Neighborhoods for Active Kids (NfAK)	2	1.2
Raising healthy Eating and Active Living Kids in Alberta (REAL Kids Alberta)	2	1.2
Texas Childhood Obesity Prevention Policy Evaluation (T-COPPE)	2	1.2
Other	37	22.8
Not reported	56	34.6
*Physical activity assessment method*		
Self- or proxy-report	118	72.8
Device	30	18.6
Both	14	8.7
*Physical activity type*^b^		
Active travel	86	53.1
Non-type-specific physical activity	70	43.5
Active independent mobility	7	4.3
Sports participation	3	1.9
Active outdoor play	2	1.2
*Neighbourhood environment assessment method*		
Neighbourhood Environment Walkability Scale^c^	48	29.8
Safe Routes to School Program questionnaire	7	4.3
Other questionnaire	33	20.5
Questionnaire name not reported^d^	79	48.8
*Neighbourhood environment attribute*^e^		
General crime/personal safety	66	40.7
General safety	60	37.0
General traffic safety	46	28.4
Social capital and/or cohesion	41	25.3
Other cross-category scores	39	24.7
Access to sports and recreational facilities, parks, and/or playgrounds	38	23.5
Traffic volume and/or speed	38	22.8
More greenery and/or better aesthetics	36	22.2
Availability of walking and/or cycling infrastructure	27	16.7
Street connectivity	26	16.0
Distance to school	22	13.6
Land use mix / destination mix score	22	13.6
Residential density	21	13.0
Availability of pedestrian crossings and signals	19	11.7
General walking and/or cycling infrastructure score	17	10.5
Physical activity of others in the neighbourhood	17	10.5
Good street lighting	13	8.6
Access to public transport	11	8.0
General activity friendliness	11	6.8
Hilliness	11	6.8
Busy/dangerous intersections and crossings	8	4.9
Presence of crossing guards	7	4.3
Access to shopping places and food outlets	5	3.1
Quality of walking and/or cycling infrastructure	5	3.1
Number of roads to cross en route	4	2.5
High air pollution	4	2.5
Major physical barriers limiting the number of routes	3	1.9
Availability of parking	2	1.2
General physical barriers score	2	1.2
Social disorder	2	1.2

^a^Number of papers

^b^The percentages do not add up to 100%, because some papers included data on more than one type of physical activity

^c^Neighbourhood Environment Walkability Scale (NEWS), NEWS-Abbreviated, NEWS-Africa, or NEWS for Youth

^d^A questionnaire developed specifically for the purpose of the given study or an existing questionnaire (or a subset of its items), whose name has not been reported in the paper

^e^The percentages do not add up to 100%, because some papers included data on than one environmental attribute

### Physical activity measures

Approximately one fifth (18.5%) of the papers assessed physical activity using devices, self/proxy-reports were used in 72.8% of the papers, and 8.6% of the papers used both methods. Most (53.1%) of the included papers assessed active travel, almost half (43.5%) of the included papers assessed non-type-specific physical activity (mainly moderate-to-vigorous physical activity), while only a few papers assessed active independent mobility (4.3%), sports participation (1.9%), and active outdoor play (1.3%).

### Measures of parental perceptions of neighbourhood environment

Nearly one third of included papers reported using some version of NEWS to assess parental perceptions of neighbourhood environment, while almost half of the papers used a questionnaire developed specifically for the purpose of the given study or an existing questionnaire (or a subset of its items) whose name has not been reported in the paper. The most commonly analysed category of neighbourhood environment variables was combined scores (in 56.2% of papers), followed by traffic safety (49.4%), access to destinations and services (41.4%), crime/personal safety (40.7%), social environment (34.6%), walking and cycling infrastructure (26.5%), greenery and aesthetics (22.2%), street connectivity (16.0%), residential density (13.0%), physical barriers (8.6%), and environmental hazards (2.5%).

### Parental perceptions of neighbourhood environment and physical activity among children

A total of 51 associations of parental perceptions with physical activity among children were analysed, of which 86.3% were found to be non-significant, indeterminate, or inconsistent. For children’s active travel, we found evidence of an unfavourable association with parental perceptions of distance to school (5 out of 7 associations; pooled sample size in the studies showing significant association [*n*] = 14,113; low quality of evidence) and favourable associations with parental perceptions of access to public transport (3 out of 5 associations; *n* = 1415; low quality of evidence), good street lighting (2 out of 3 associations; *n* = 1627; low quality of evidence), and presence of crossing guards (2 out of 3 associations; *n* = 1735; low quality of evidence; Table 3). Parental perceptions of access to sports and recreational facilities, parks, and/or playgrounds were found to be favourably associated with sports participation among children (2 out of 3 associations; *n* = 3890; low quality of evidence). For children’s active outdoor play, we found evidence of favourable associations with parental perceptions of access to sports and recreational facilities, parks, and/or playgrounds and general traffic safety (in 1 study only; *n* = 1081 for both; low quality of evidence).

**Table 3:** Associations between parental perceptions of neighbourhood environment and physical activity among children aged 5–11 years

	**Unfavourable (-)**	**Favourable ( +)**	**Mixed/inconsistent/non-significant (?)**	**Summary code**	**Quality of evidence**
**Active travel**					
*Combined scores*					
- General activity friendliness	[153]	[94]		?	very low
- General safety		[14], [78], [168](M)	[55], [72], [76], [138, 139], [168](F)	??	low
- Other cross-category scores	[153]	[28](M), [144]	[28](F), [120], [154, 155], [162]	??	low
*Access to destinations and services*					
- Access to public transport		[30](F), [138, 139], [142]	[30](M), [33]	+	low
- Access to shopping places and food outlets		[193]	[142]	?	low
- Access to sports and recreational facilities, parks, and/or playgrounds			[30, 180], [162]	?	low
- Distance to school	[38], [55], [78], [95], [138, 139]		[122], [194]	--	low
- Land use mix / destination mix score			[142], [162]	?	low
*Physical barriers*					
- Hilliness			[33], [194]	?	low
*Walking and cycling infrastructure*					
- Availability of walking and/or cycling infrastructure		[149]	[33], [38], [55], [128, 129], [142], [162], [194]	??	low
- Quality of walking and/or cycling infrastructure		[149]	[94]	?	low
*Greenery and aesthetics*					
- More greenery and/or better aesthetics			[33], [142], [162], [194]	??	low
*Street connectivity*					
- Street connectivity			[33], [142], [162]	?	low
*Residential density*					
- Residential density			[162]	? (SSE)	low
*Crime/personal safety*					
- General crime/personal safety		[122], [128, 129]	[30, 180], [33], [76], [95], [142], [149], [162], [172], [194]	??	low
*Traffic safety*					
- Availability of pedestrian crossings and signals		[142], [149]	[30, 180], [33]	?	low
- Busy/dangerous intersections and crossings			[55]	? (SSE)	low
- General traffic safety	[193]	[28](M), [122], [166], [194]	[28](F), [33], [38], [76], [95], [162], [172]	??	very low
- Good street lighting		[142], [172]	[194]	+	low
- Presence of crossing guards		[128, 129], [149]	[38]	+	low
- Traffic volume and/or speed	[14], [55], [149], [194]		[30, 180], [33], [128, 129], [142]	??	low
*Environmental hazards*					
- High air pollution			[38]	? (SSE)	very low
*Social environment*					
- Physical activity of others in the neighbourhood			[55], [149], [194]	?	low
- Social capital and/or cohesion		[122], [149]	[33], [76], [142], [153], [154, 155], [168], [172], [193]	??	low
**Non-type-specific physical activity**					
*Combined scores*					
- General activity friendliness		[90]	[148]	?	low
- General safety		[80], [96]	[52](F), [61, 63], [67], [69], [70], [72], [90], [132], [159], [184]	??	low
- Other cross-category scores		[90], [172]	[32], [61, 63], [72], [96], [107], [118], [160], [162], [184]	??	low
*Access to destinations and services*					
- Access to public transport			[133](F), [142]	?	low
- Access to shopping places and food outlets			[133](F), [142]	?	low
- Access to sports and recreational facilities, parks, and/or playgrounds		[79], [133](F), [184], [198]	[52](F), [107], [140], [159], [162], [179]	??	low
- Land use mix / destination mix score			[118], [140], [142], [162]	??	low
*Physical barriers*					
- Hilliness			[118]	? (SSE)	low
*Walking and cycling infrastructure*					
- Availability of walking and/or cycling infrastructure		[133](F)	[140], [142]	?	low
- General walking and/or cycling infrastructure score			[118], [162], [179]	?	low
*Greenery and aesthetics*					
- More greenery and/or better aesthetics			[118], [133](F), [142], [162], [179]	??	low
*Street connectivity*					
- Street connectivity		[140]	[118], [133](F), [142], [162], [179]	??	low
*Residential density*					
- Residential density	[118]		[35], [140], [162]	?	low
*Crime/personal safety*					
- General crime/personal safety	[35](F)		[32], [35](M), [118], [125, 179], [133](F), [140], [142], [150], [162], [178]	??	low
*Traffic safety*					
- Availability of pedestrian crossings and signals			[142], [150]	?	low
- General traffic safety			[32], [79], [140], [162], [179], [184], [198]	??	low
- Good street lighting			[142], [150]	?	low
- Traffic volume and/or speed		[133](F)	[118], [142], [150]	?	low
*Social environment*					
- Physical activity of others in the neighbourhood		[133](F)	[35]	?	low
- Social capital and/or cohesion			[35], [107], [125], [142], [178], [184]	??	low
- Social disorder			[69]	? (SSE)	very low
**Sports participation**					
*Combined scores*					
- General safety			[54], [189]	?	low
*Access to destinations and services*					
- Access to sports and recreational facilities, parks, and/or playgrounds		[54], [198]	[189]	+	low
*Traffic safety*					
- General traffic safety			[198]	? (SSE)	low
**Active outdoor play**					
*Access to destinations and services*					
- Access to sports and recreational facilities, parks, and/or playgrounds		[65]		+ (SSE)	low
*Crime/personal safety*					
- General crime/personal safety			[65]	? (SSE)	low
*Traffic safety*					
- General traffic safety		[65]		+ (SSE)	low

Notes: ( +) ≥ 60% of associations were favourable; (+ +) ≥ 60% of associations were favourable and ≥ 4 studies found a favourable association; (-) ≥ 60% of associations were unfavourable; (--) ≥ 60% of associations were unfavourable and ≥ 4 studies found an unfavourable association; (?) mostly non-significant, indeterminate, or inconsistent associations; (??) frequently studied association for which findings were generally mixed, inconsistent or non-significant; when there were two or more papers from the same study, their findings were combined and their citations were enclosed in single brackets; (F) female sample; (M) male sample; (SSE) single-study evidence should be interpreted with caution, as it has not been verified in other studies

### Parental perceptions of neighbourhood environment and physical activity among adolescents

A total of 51 associations of parental perceptions with physical activity among adolescents were analysed, of which 86.3% were found to be non-significant, indeterminate, or inconsistent. For adolescents’ active travel, we found evidence of an unfavourable association with parental perceptions of distance to school (3 out of 4 associations; *n* = 2328; low quality of evidence) and favourable associations with parental perceptions of quality of walking and/or cycling infrastructure (in 1 study only; *n* = 1802; low quality of evidence) and presence of crossing guards (in 1 study only; *n* = 628; low quality of evidence; Table 4). Parental perceptions of access to sports and recreational facilities, parks, and/or playgrounds were found to be favourably associated with non-type-specific physical activity level among adolescents (3 out of 4 associations; *n* = 12,320; low quality of evidence). Adolescents’ active independent mobility was found to be unfavourably associated with parental perceptions of availability of pedestrian crossings and signals, presence of busy/dangerous intersections and crossings, and high air pollution (in 1 study only; *n* = 243 for all; very low quality of evidence).

**Table 4:** Associations between parental perceptions of neighbourhood environment and physical activity among adolescents aged 12–17 years

	**Unfavourable (-)**	**Favourable ( +)**	**Mixed/inconsistent/non-significant (?)**	**Summary code**	**Quality of evidence**
**Active travel**					
*Combined scores*					
- General activity friendliness		[75]	[124]	?	low
- General safety			[55], [56], [74], [97], [110]	??	low
- Other cross-category scores			[104], [110]	?	low
*Access to destinations and services*					
- Access to public transport			[33], [58, 59]	?	low
- Access to shopping places and food outlets			[58, 59]	?	low
- Access to sports and recreational facilities, parks, and/or playgrounds		[58, 59]	[74], [124]	?	low
- Distance to school	[55], [58, 59], [115]		[197]	-	low
- Land use mix / destination mix score		[74](F)	[58, 59], [71], [74](M), [124]	??	low
*Physical barriers*					
- General physical barriers score			[58, 59]	?	low
- Hilliness			[33]	? (SSE)	low
*Walking and cycling infrastructure*					
- Availability of walking and/or cycling infrastructure			[33], [115], [151]	?	low
- General walking and/or cycling infrastructure score			[55], [58, 59], [71], [124]	??	low
- Quality of walking and/or cycling infrastructure		[151]		+ (SSE)	low
*Greenery and aesthetics*					
- More greenery and/or better aesthetics			[33], [58, 59], [71], [124], [151]	??	low
*Street connectivity*					
- Street connectivity		[71]	[33], [58, 59], [124], [151]	??	low
*Residential density*					
- Residential density			[58, 59], [124]	?	low
*Crime/personal safety*					
- General crime/personal safety		[103], [197]	[33], [58, 59], [71], [115], [124], [175]	??	low
*Traffic safety*					
- Availability of pedestrian crossings and signals		[33]	[175]	?	low
- Busy/dangerous intersections and crossings	[55]		[115], [175]	?	low
- General traffic safety			[33], [58, 59], [71], [75], [103], [110]	??	low
- Good street lighting			[71], [175]	?	low
- Presence of crossing guards		[115]		+ (SSE)	low
- Traffic volume and/or speed	[55], [74](F)		[33], [74](M), [103], [115], [175], [197]	??	low
*Environmental hazards*					
- High air pollution			[175]	? (SSE)	low
*Social environment*					
- Physical activity of others in the neighbourhood			[55], [175]	?	low
- Social capital and/or cohesion		[115]	[33]	?	low
**Non-type-specific physical activity**					
*Combined scores*					
- General safety		[77], [130]	[57], [60]	?	low
- Other cross-category scores		[77]	[32], [89], [103]	?	low
*Access to destinations and services*					
- Access to sports and recreational facilities, parks, and/or playgrounds		[77], [111], [130]	[89]	+	low
- Land use mix / destination mix score			[89]	? (SSE)	low
*Walking and cycling infrastructure*					
- General walking and/or cycling infrastructure score			[89], [130]	?	low
*Greenery and aesthetics*					
- More greenery and/or better aesthetics			[89], [102, 156]	?	low
*Street connectivity*					
- Street connectivity			[89]	? (SSE)	low
*Residential density*					
- Residential density			[89]	? (SSE)	low
*Crime/personal safety*					
- General crime/personal safety		[103]	[89], [102, 156]	?	low
*Traffic safety*					
- General traffic safety			[32], [102, 156]	?	low
- Traffic volume and/or speed			[103]	? (SSE)	low
**Active independent mobility**					
*Crime/personal safety*					
- General crime/personal safety			[116]	? (SSE)	very low
*Traffic safety*					
- Availability of pedestrian crossings and signals	[116]			- (SSE)	very low
- Busy/dangerous intersections and crossings	[116]			- (SSE)	very low
- Good street lighting			[116]	? (SSE)	very low
- Traffic volume and/or speed			[116]	? (SSE)	very low
*Environmental hazards*					
- High air pollution	[116]			- (SSE)	very low
*Social environment*					
- Physical activity of others in the neighbourhood			[116]	? (SSE)	very low
**Sports participation**					
*Combined scores*					
- General safety			[27]	? (SSE)	low
*Access to destinations and services*					
- Access to public transport			[27]	? (SSE)	low
- Access to sports and recreational facilities, parks, and/or playgrounds			[27], [136]	?	low
*Social environment*					
- Social capital and/or cohesion			[27]	? (SSE)	low
**Active outdoor play**					
*Access to destinations and services*					
- Access to sports and recreational facilities, parks, and/or playgrounds			[65]	? (SSE)	low
*Crime/personal safety*					
- General crime/personal safety			[65]	? (SSE)	low
*Traffic safety*					
- General traffic safety			[65]	? (SSE)	low

Notes: ( +) ≥ 60% of associations were favourable; (+ +) ≥ 60% of associations were favourable and ≥ 4 studies found a favourable association; (-) ≥ 60% of associations were unfavourable; (--) ≥ 60% of associations were unfavourable and ≥ 4 studies found an unfavourable association; (?) mostly non-significant, indeterminate, or inconsistent associations; (??) frequently studied association for which findings were generally mixed, inconsistent or non-significant; when there were two or more papers from the same study, their findings were combined and their citations were enclosed in single brackets; (F) female sample; (M) male sample; (SSE) single-study evidence should be interpreted with caution, as it has not been verified in other studies

### Parental perceptions of neighbourhood environment and physical activity in the mixed-age group including children and adolescents

A total of 74 associations of parental perceptions with physical activity in the mixed-age group were analysed, of which 94.6% were found to be non-significant, indeterminate, or inconsistent. For active travel in the mixed-age group, we found evidence of a favourable association with parental perceptions of availability of parking (in 1 study only; *n* = 365; low quality of evidence) and an unfavourable association with parental perceptions of distance to school (8 out of 13 associations; *n* = 5410; low quality of evidence; Table 5). In the mixed-age group, we found evidence of an unfavourable association between parental perceptions of social disorder and non-type-specific physical activity (in 1 study only; *n* = 1041; low quality of evidence). More favourable other cross-category scores calculated based on parental perceptions of the neighbourhood environment were found to be favourably associated with sports participation in the mixed-age group (in 1 study only; *n* = 64,076; low quality of evidence).

**Table 5:** Associations between parental perceptions of neighbourhood environment and physical activity in the mixed-age group including children and adolescents (age: 5–17 years)

	**Unfavourable (-)**	**Favourable ( +)**	**Mixed/inconsistent/non-significant (?)**	**Summary code**	**Quality of evidence**
**Active travel**					
*Combined scores*					
- General activity friendliness			[93, 94], [188]	?	low
- General safety	[196]	[53], [64], [82], [83]	[54], [62, 188], [66], [146], [165], [181, 182], [187]	??	very low
- Other cross-category scores		[119]	[85], [106], [188]	?	low
*Access to destinations and services*					
- Access to public transport			[30, 180]	?	low
- Access to sports and recreational facilities, parks, and/or playgrounds		[30, 180]	[36, 161], [54], [66], [84, 92], [187]	??	low
- Availability of parking		[9]		+ (SSE)	low
- Distance to school	[37], [92](M), [114], [115], [157], [158], [174], [173]		[10], [31], [92](F), [113], [196]	--	low
- Land use mix / destination mix score			[9], [36, 161], [84, 92], [187]	??	low
*Physical barriers*					
- Hilliness	[157]		[9], [36, 161], [99], [181, 182]	??	low
- Major physical barriers limiting the number of routes			[9], [66]	?	low
*Walking and cycling infrastructure*					
- Availability of walking and/or cycling infrastructure		[37], [99], [115], [174]	[36, 161], [84, 92], [85], [112], [114], [157], [182], [196]	??	low
- General walking and/or cycling infrastructure score			[9], [31], [186], [187]	??	low
- Quality of walking and/or cycling infrastructure			[84, 92]	?	low
*Greenery and aesthetics*					
- More greenery and/or better aesthetics			[9], [36, 161], [66], [84, 92], [85], [99], [112], [157], [186], [187], [196]	??	low
*Street connectivity*					
- Street connectivity		[10]	[9], [36, 161], [84, 92], [99], [174], [186]	??	low
*Residential density*					
- Residential density		[180]	[9], [36, 161], [84, 92], [85], [99], [186]	??	low
*Crime/personal safety*					
- General crime/personal safety		[134]	[9], [30, 180], [31], [36, 161], [37], [66, 115], [84, 92], [85], [99], [93, 94], [112], [113], [114], [157], [173], [174], [181, 182], [185], [186], [192], [196]	??	low
*Traffic safety*					
- Availability of pedestrian crossings and signals		[36, 161]	[30, 180], [62], [181, 182], [192]	??	low
- Busy/dangerous intersections and crossings	[36, 161], [157]		[99], [114], [115]	?	low
- General traffic safety			[37], [66], [84, 92], [85], [93, 94], [84], [112], [134], [174], [181, 182], [186], [192]	??	low
- Good street lighting			[36, 161], [99], [157], [192]	??	low
- Number of roads to cross en route		[37]	[62], [180]	?	low
- Presence of crossing guards		[115]	[31], [114]	?	low
- Traffic volume and/or speed	[53], [157]	[66]	[9], [31], [36, 161], [99], [113], [114], [115], [173], [174], [180], [185], [192], [196]	??	very low
*Environmental hazards*					
- High air pollution			[192]	? (SSE)	low
*Social environment*					
- Physical activity of others in the neighbourhood		[37], [157]	[36, 161], [62, 188], [66], [99], [192]	??	low
- Social capital and/or cohesion		[53], [115], [137](F)	[99], [112], [137](M), [163, 164], [174], [196]	??	low
**Non-type-specific physical activity**					
*Combined scores*					
- General activity friendliness		[41]	[170], [126]	?	low
- General safety		[41], [88, 109], [100], [171]	[66], [117], [169], [170], [176, 177]	??	low
- Other cross-category scores		[135], [167], [195]	[91], [98], [100], [106], [121], [171]	??	low
*Access to destinations and services*					
- Access to public transport			[81]	? (SSE)	low
- Access to sports and recreational facilities, parks, and/or playgrounds		[66], [117]	[11], [81], [84], [86], [87], [167], [170]	??	low
- Availability of parking			[183]	? (SSE)	very low
- Distance to school			[92]	? (SSE)	low
- Land use mix / destination mix score		[117], [183]	[9], [84, 92], [87], [143], [171], [186]	??	low
*Physical barriers*					
- Hilliness			[9], [183]	?	low
- Major physical barriers limiting the number of routes			[9], [66], [183]	?	low
*Walking and cycling infrastructure*					
- Availability of walking and/or cycling infrastructure			[84, 92]	?	low
- General walking and/or cycling infrastructure score		[9]	[87], [143], [183]	?	low
- Quality of walking and/or cycling infrastructure			[84, 92]	?	low
*Greenery and aesthetics*					
- More greenery and/or better aesthetics			[9], [66], [84, 92], [87], [91], [100], [117], [143], [183]	??	low
*Street connectivity*					
- Street connectivity			[9], [84, 92], [87], [143], [183]	??	low
*Residential density*					
- Residential density		[143]	[9], [84, 92], [87], [183]	??	low
*Crime/personal safety*					
- General crime/personal safety		[143]	[9], [66], [73], [84, 92], [86], [87], [127, 145], [143], [183]	??	low
*Traffic safety*					
- Availability of pedestrian crossings and signals			[81], [143]	?	low
- General traffic safety			[66], [73], [81, 86], [84, 92], [87], [127, 145], [143]	??	low
- Number of roads to cross en route			[81]	? (SSE)	low
- Traffic volume and/or speed	[81](M)	[41]	[9], [66], [81](F), [183]	??	very low
*Social environment*					
- Physical activity of others in the neighbourhood		[66]	[117]	?	low
- Social capital and/or cohesion		[91], [100], [117], [152], [171]	[121], [123], [127, 145], [135], [176, 177]	??	low
- Social disorder	[41]			- (SSE)	low
**Active independent mobility**					
*Combined scores*					
- General activity friendliness			[188]	? (SSE)	very low
- General safety			[105, 190, 191], [188]	?	low
- Other cross-category scores			[105, 190, 191], [188]	?	low
*Crime/personal safety*					
- General crime/personal safety			[108]	? (SSE)	low
*Traffic safety*					
- Availability of pedestrian crossings and signals			[108], [190, 191]	?	low
*Social environment*					
- Physical activity of others in the neighbourhood			[188]	? (SSE)	very low
**Sports participation**					
*Combined scores*					
- General safety			[101]	? (SSE)	low
- Other cross-category scores		[101]		+ (SSE)	low
*Access to destinations and services*					
- Access to sports and recreational facilities, parks, and/or playgrounds			[84]	? (SSE)	low
- Land use mix / destination mix score			[84]	? (SSE)	low
*Walking and cycling infrastructure*					
- Availability of walking and/or cycling infrastructure			[84]	? (SSE)	low
- Quality of walking and/or cycling infrastructure			[84]	? (SSE)	low
*Greenery and aesthetics*					
- More greenery and/or better aesthetics			[84], [101]	?	low
*Street connectivity*					
- Street connectivity			[84]	? (SSE)	low
*Residential density*					
- Residential density			[84]	? (SSE)	low
*Crime/personal safety*					
- General crime/personal safety			[84]	? (SSE)	low
*Traffic safety*					
- General traffic safety			[84]	? (SSE)	low
*Social environment*					
- Social capital and/or cohesion		[101]	[141]	?	low
**Active outdoor play**					
*Combined scores*					
- Other cross-category scores			[91]	? (SSE)	low
*Walking and cycling infrastructure*					
- Availability of walking and/or cycling infrastructure			[147]	? (SSE)	low
*Greenery and aesthetics*					
- More greenery and/or better aesthetics			[91]	? (SSE)	low
*Traffic safety*					
- General traffic safety			[147]	? (SSE)	low
*Social environment*					
- Social capital and/or cohesion			[91], [131]	?	low

Notes: ( +) ≥ 60% of associations were favourable; (+ +) ≥ 60% of associations were favourable and ≥ 4 studies found a favourable association; (-) ≥ 60% of associations were unfavourable; (--) ≥ 60% of associations were unfavourable and ≥ 4 studies found an unfavourable association; (?) mostly non-significant, indeterminate, or inconsistent associations; (??) frequently studied association for which findings were generally mixed, inconsistent or non-significant; when there were two or more papers from the same study, their findings were combined and their citations were enclosed in single brackets; (F) female sample; (M) male sample; (SSE) single-study evidence should be interpreted with caution, as it has not been verified in other studies

### Methodological quality of included papers

Only one paper was of high methodological quality [79], 14.2% were of moderate quality, and the remaining 85.2% were of low quality (Table 6 and Additional file 5). Most papers (90.7%) were based on studies using a cross-sectional design, while the remaining used data from longitudinal (8.6%) and experimental studies (0.6%). In 82.7% of the papers, the sample size was ≥ 300, while the remaining 15.4% of the papers included between 100 and 299 participants. In approximately one-third (27.8%) of the papers, the study areas (or participant recruitment) were stratified by key attributes of the neighbourhood environment. The response rate was ≥ 60% (or the sample was representative of the population) in 28.4% of the papers. To assess parental perceptions of the neighbourhood environment, 50.0% of the papers utilized valid and reliable questionnaires. To assess physical activity, 46.3% of the papers used valid and reliable measurement tools. Adjustments for key socio-demographic factors were performed in 66.0% of the papers, while only 3.7% of the papers adjusted the analyses for self-selection into neighbourhoods. Analytical approaches in 50.6% and 83.3% of the papers accounted for area-level clustering and distributional assumptions, respectively. In all included papers, analyses were conducted and presented correctly, including the calculation of effect sizes and their statistical significance, standard errors, or confidence intervals.

**Table 6:** Methodological quality of included papers

**Item** [points]	**%**
*Study design*	
Cross-sectional [0]	90.7
Longitudinal [1]	8.6
Experimental [2]	0.6
*Sample size*	
< 100 [0]	1.9
100 – 299 [1/2]	15.4
≥ 300 [1]	82.7
Study areas or participant recruitment stratified by key environmental attributes [1]	27.8
*Response rate*	
≥ 60% [1]	28.4
< 60% [2]	1.2
Parental perceptions of neighbourhood environment measures shown to be valid and reliable^a^ [1]	50.0
Physical activity outcome measures shown to be valid and reliable^a^ [1]	46.3
Adjustment for key socio-demographic characteristics [1]	66.0
Adjustment for self-selection [1]	3.7
Analytical approach accounted for area-level clustering [1/3]	50.6
Analytical approach accounted for distributional assumptions [1/3]	83.3
Analyses conducted and presented correctly [1/3]	100
*Overall methodological quality*	
Low	85.2
Medium	14.2
High	0.6

^a^The assessment of validity and reliability was based on the interpretation provided by the authors of included studies or by the authors of a validation study of the given questionnaire

### Quality of evidence

The quality of evidence was deemed as “very low” for 9.7% and “low” for 90.3% of the associations (Tables 3, 4 and 5). Given that the evidence for all associations was based mostly on observational studies, the starting quality of evidence in all the respective evaluations was considered to be “low” (Additional file 6). The most prevalent indications for downgrading the quality of evidence were risk of bias due to large representation of studies with low methodological quality and indirectness due to overrepresentation of studies from high-income countries, found for 86.4% and 99.4% of associations, respectively. The other indications for downgrading the quality of evidence were much less represented.

## Discussion

### Key findings

The most consistent finding was that a greater distance to school is unfavourably associated with active travel. Evidence of this association was found in children, adolescents, and mixed-age group. There was some consistency in evidence on favourable associations of: (1) access to public transport, good street lighting, and presence of crossing guards with active travel among children; (2) access to sports and recreational facilities, parks and/or playgrounds with sports participation among children; and (3) access to sports and recreational facilities, parks, and/or playgrounds with non-type-specific physical activity among adolescents. In addition, several associations were found in individual studies only, while others were mostly non-significant, indeterminate, or inconsistent. These findings should be interpreted with caution, because the quality of evidence ranged from very low to low.

### Access to destinations and services

Three correlates of physical activity for which our review found some consistency in evidence are characteristics of access to destinations and services. They include access to public transport, access to sports and recreational facilities, parks and/or playgrounds, and distance to school.

The favourable association between access to public transport and active travel among children has been suggested in a previous review by Davison and Lawson [199], but their finding was based on a single study. The amount of evidence on this topic has since increased, and based on our findings we can now conclude that there is some consistency in evidence supporting this association. Using public transport is not considered as active travel. However, it is often needed to engage in some form of active travel to get to and from public transport stops. This would explain why parental perception of access to public transport is associated with more active travel.

Findings of previous reviews on the association between access to sports and recreational facilities, parks and/or playgrounds and physical activity of children and adolescents were inconsistent. For example, Davison and Lawson [199] suggested that proximity of playgrounds and parks and availability of recreational facilities are favourably associated with non-type-specific physical activity in a mixed-age group including children and adolescents. Similarly, Ding et al. [12] found that access to recreational facilities and open spaces was favourably associated with non-type-specifc physical activity among children. However, two more recent reviews suggested that this association was non-significant in most previous studies among children [8] and in a mixed-age group including children and adolescents [18]. The inconsistency in findings between the reviews may be due to differences in their methodologies (e.g. different methods for data synthesis) and/or due to changes in available evidence over time. It should be noted that the reviews included only studies that used objective measures of the environment [12] or they combined studies that assessed perceived and objective measures [8, 18, 199]. Our review provided novel evidence supporting favourable associations between parental perception of access to sports and recreational facilities, parks and/or playgrounds with non-type-specific physical activity among adolescents and sports participation among children. A recent review found that children and adolescents accumulate the highest amount of moderate-to-vigorous physical activity at home and in recreational facilities [200], which may explain our finding.

Furthermore, our finding of an unfavourable association between parental perceptions of distance to school and active travel in children, adolescent, and a mixed-age group is consistent with previous systematic reviews [8, 15, 16, 201]. According to our findings, greater distance to school is likely to discourage parents from letting their children actively commute to and from school. For example, in some cases active travel to/from school is not even feasible, because the school is located too far away from home. Cole et al. proposed that the feasible distance to replace passive travel with walking and cycling is 1.3 km and 4.2 km, respectively [202]. However, it is also logical to conclude that if the distance from home to school is very short, the contribution of active travel to/from school to achieving the recommended amount of moderate-to-vigorous physical activity (i.e. 420 min/week) will be small. Therefore, there is an optimal range of distances from home to school that would yield significant contributions to the accumulation of health-enhancing doses of physical activity in children and adolescents. Elucidating such optimal range would be an interesting topic for future studies. However, regardless of the optimal distance, it is important to acknowledge that even very short bouts of active travel contribute to overall physical activity and that any engagement in physical activity is better than none [2].

In addition, we found evidence of favourable associations between availability of parking and active travel in the mixed-age group and between access to sports and recreational facilities, parks and/or playgrounds and active outdoor play among children. However, these findings are based on one study only, and thefore their consistency needs to be determined in future studies.

### Traffic safety

Two correlates of physical activity for which our review found some consistency in evidence belong to traffic safety. They include good street lighting and presence of crossing guards. The favourable associations of parental perceptions of these two neighbourhood environment attributes are aligned with findings of previous reviews suggesting that parental concerns about traffic safety are among key barriers of active travel to school [15, 16, 200].

Our finding for parental perceptions of street lighting is novel, because no previous review has assessed the association of this specific variable with physical activity among children. However, in a previous systematic review of objectively measured neighbourhood environment attributes, Wong et al. identified one study on the association of streetlight density and active travel to school, and the reported association was non-significant [201]. Hence, it may be that parental perceptions of street lighting are more important predictor of children’s active travel than the actual quality of street lighting. Good street lighting improves visibility and, consequently, reduces the risk of traffic accidents [203]. It may be that the parents who perceive street lighting in their neighbourhood as adequate are less concerned about traffic accidents and are, therefore, more likely to allow their children to use active modes of transport.

The finding related to the presence of crossing guards is also novel, as this specific association has not been assessed separately in previous reviews focused on children and adolescents. It has been suggested that the presence of crossing guards may improve pedestrian safety and reduce the risk of unintentional injuries among children [204]. It may be that the parents who are aware of the presence of crossing guards in their neighbourhood are less worried about traffic accidents and are, therefore, more likely to allow their children to engage in active travel. We found evidence of an association between presence of crossing guards and active travel also among adolescents. However, given that the evidence comes from a single study, this association remains to be verified in future research.

Furthemore, evidence on the associations of general traffic safety with active outdoor play among children, as well as of the availability of pedestrian crossings and signals, presence of busy/dangerous intersections, and high air pollution with active independent mobility among adolescents comes from individual studies. Therefore, these associations need to be confirmed in future research.

### Other characteristics of neighbourhood environment

The association between parental perceptions of quality of walking and/or cycling infrastructure and active travel among adolescents was found in a single study. Similarly, the associations between other cross-category scores and sports participation and between social disorder and non-type-specific physical activity in the mixed-age group were found in individual studies only. Therefore, more research is needed to determine consistency of these associations. However, it should be noted that the finding for other cross-category scores comes from a study with a very large, population representative sample, which means that it is likely more generalizable than other findings, coming from smaller individual studies.

### Non-significant, indeterminate, or inconsistent associations

The fact that we the vast majority of associations were non-significant, indeterminate, or inconsistent could suggest that many neighbourhood environment attributes are not associated with physical activity. However, it may also be due to relatively small sample sizes in some of the included studies and attenuation of associations due to imperfect reliability of the questionnaires for the assessment of parental perceptions of neighbourhood environment and children’s physical activity. It is also possible that some of the associations vary across different regions and sociocultural contexts, which could explain inconsistency in findings from different studies.

### Implications for policy and practice

Parental perceptions are partially shaped by the actual characteristics of the neighbourhood environment [205]. Therefore, public policies and interventions should focus on improving the neighbourhood environment attributes for which we found at least some consistency in their associations with physical activity among children and adolescents, including access to destinations and services and traffic safety. In specific, it may be beneficial to ensure that: (1) the policies on school catchment areas and the aerial distribution of schools enable most children and adolescents to relatively quickly get to and from their schools using active modes of transport; (2) public transport, sports and recreational facilities, parks, and/or playgrounds are accessible to most children and adolescents; (3) street lighting is adequate; and (4) there are crossing guards on main intersections. However, parental perceptions of neighbourhood environment may also be influenced by factors other than the actual environmental characteristics [206]. For example, parents may not necessarily be aware of the suitability of the route to school for active travel and availability of recreational facilities in their neighbourhood. They may also have unjustified concerns about traffic safety in the neighbourhood. Therefore, interventions should aim to achieve good alignment between the actual characteristics of neighbourhood environment and parental perceptions of the environment.

### Recommendations for future research

Findings of this review have several implications for future research. First, more research focusing on adolescents is needed, because only 15% of the studies included in this review were conducted specifically in this age group. Second, more longitudinal and (quasi)experimental studies are needed to establish prospective and causal relationships, because a vast majority of the included studies were cross-sectional. Third, there is a need for more diversity in future research in terms of study location, because more than 70% of the included studies were conducted in Australia, Belgium, Canada, New Zealand, and the United States. A better representation of studies from low- and middle-income countries should be achieved, to help meet the United Nations recommendations for the prevention and control of non-communicable diseases [207]. However, it should be noted that our literature search was conducted using English keywords and restricted to publications in Chinese and English, which may have contributed to the overrepresentation of included studies from English-speaking countries. Fourth, some neighbourhood environment attributes have been studied much less than others. When possible, future studies should consider covering a wide range of neighbourhood environment attributes, especially the ones that were underrepresented in previous research. Fifth, parental perceptions of neighbourhood environment were assessed using various questionnaires. A relatively large number of studies used newly developed questionnaires or did not state which existing questionnaire was used. To improve comparability of findings between studies, transparent reporting of measurement methods and the use of standardised and widely used questionnaires, such as NEWS [29] and NEWS for Youth [208] should be facilitated in future research. Sixth, different types of parental perceptions of neighbourhood environment were assessed. In some studies, parents provided evaluative assessments of the neighbourhood environment denoting individual preferences for, or level of satisfaction with, environmental features (e.g. “I am satisfied with the number of pedestrian crossings in my neighbourhood.”), while in others the perceived presence or level of specific environmental features were assessed (e.g. “There are no lights/crossings in my area.”). In some cases, the two types of assessment were combined into a single score. Evaluative assessments of the neighbourhood environment are more likely to be influenced by affect and other psychological factors than their perceived presence/level counterparts and are often based on items that do not quantify or accurately describe the environmental feature being measured (e.g., the item “I am satisfied with the number of pedestrian crossings” does not provide any indication of the number of crossings a person is satisfied with). Therefore, future studies should make a clear distinction between the two types of assessment. Seventh, parental perceptions of different neighbourhood environment attributes may have complex interrelations. Future studies should consider exploring their mutual confounding, mediation, moderation, and suppressor effects. Eighth, some of the included papers reported inconsistent findings for female and male samples. Exploring possible differences in associations among females and males was beyond the scope of this review, but this may be an interesting topic for future studies. Ninth, future studies should consider using samples that are large enough to achieve adequate statistical power even if the true effect size is small. Tenth, a better representations of fathers among parent respondents should be achieved, as they may differ from mothers in terms of their perceptions of neighbourhood environment and influence on children’s physical activity. Finally, time spent in physical activity is a part of time-use composition, including also sedentary behaviour and sleep. Therefore, methodological papers have recommended to use compositional data analysis to adequately address interdependency of these time-use components, even if only one of the components is the variable of interest [209–211]. However, none of the included studies has used compositional data analysis. Future studies could consider taking an integrative approach to analysing these behaviours as conceptualised in the framework for Viable Integrative Research in Time-Use Epidemiology (VIRTUE) [212].

### Strengths and limitations of the review

The key strengths of this systematic review are as follows: (1) the literature search was conducted in eight bibliographic databases, which enable us to identify a large number of relevant studies; (2) the focus was exclusively on parental perceptions of neighbourhood environment (as opposed to combining objective measures and perceptions of neighbourhood environment), which enabled drawing specific conclusion about this particular and highly relevant exposure variable; (3) when possible, the evidence synthesis was performed separately for child and adolescent samples, which enabled drawing specific conclusions for each of the age groups; and (4) evidence was synthesised separately for active travel, non-type-specific physical activity, active independent mobility, sports participation, and active outdoor play, which enabled drawing conclusions for each of the physical activity types separately.

There are also several limitations to acknowledge. First, for the purpose of evidence synthesis we aggregated related questionnaire items, in some cases even if they refer to somewhat different neighbourhood environment attributes. For example, items such as “not enough sidewalks”, “not enough bike paths”, and “there is no place to leave the bicycle” were all considered as “availability of walking and/or cycling infrastructure”. This was necessary, because some of the specific questionnaire items were covered by one or few studies only. Second, the classification of some neighbourhood environment attributes into broader categories was not straightforward. For example, hilliness was classified as a physical barrier, according to the factor analysis in a previous study [29]. However, some individuals might actually perceive hilliness as an enabler for physical activities such as mountain biking or alpine skiing. Third, we did not conduct meta-analyses to statistically combine results of the included studies. We selected the current approach, due to a large heterogeneity between studies, particularly in terms of analytical approaches and measures of exposure and outcome variables. Future reviews on this topic could consider using meta-analytical methods for data synthesis [213], as done previously [214, 215]. Fourth, the methodological quality assessment was performed by one author only. However, in case of any doubts, two other authors were consulted. Fifth, due to the non-meta-analytical approach to evidence synthesis, the quality of evidence assessment could not take into account all aspects of GRADE.

## Conclusion

Parental perceptions of traffic safety and access to destinations and services are associated with different types of physical activity among children and adolescents, albeit the quality of evidence we found ranged from very low to low. In specific, a greater distance to school is associated with less active travel among both children and adolescents. In addition, among children, access to public transport, good street lighting, and presence of crossing guards are associated with more active travel, while access to sports and recreational facilities, parks and/or playgrounds is associated with higher sports participation. Among adolescents, access to sports and recreational facilities, parks, and/or playgrounds is associated with more non-type-specific physical activity.

Future systematic reviews on this topic should consider synthesising evidence for each individual exposure variable separately, exploring interrelations between neighbourhood environment attributes, assessing moderation effect of gender, and conducting meta-analyses to calculate pooled effect sizes.

## Supplementary Information

Additional file 1.

Additional file 2.

Additional file 3.

Additional file 4.

Additional file 5.

Additional file 6.

## Data Availability

All data generated and analysed in this systematic review are included in this article and additional files.
