# Supplementary material for: Associations between parental perceptions of neighbourhood environment and physical activity in children and adolescents: a systematic review including 149 studies
Source: Int J Behav Nutr Phys Act. 2025 Jun 6;22:70. doi: 10.1186/s12966-025-01733-8 (PMC12143044; doi:10.1186/s12966-025-01733-8)
Supplement: Supplementary file 1 — Additional file 1. [file 12966_2025_1733_MOESM1_ESM.docx]

**Additional file 1. Deviations from the registered protocol**

(1)

Registered in the protocol:

Literature searches will be conducted in the following bibliographic databases: CINAHL, Embase, Environment Complete, MEDLINE/PubMed, PsycInfo, ScienceDirect, Scopus, SPORTDiscus, Transportation Research Information Services (TRIS), and Web of Science Core Collection.

Deviation:

Literature searches were conducted in the following bibliographic databases: CINAHL, Embase, [Environmental Science](https://www.proquest.com/esdb?accountid=14844), MEDLINE/PubMed, PsycInfo, Scopus, SPORTDiscus, Transportation Research Information Services (TRIS), and Web of Science Core Collection.

(2)

Registered in the protocol:

The Newcastle-Ottawa Quality Assessment Scale (NOS) will be used to assess the quality of included studies (Wells et al., 2014). An adaptation of this scale on built environment and health will be used (Rachele et al., 2019; Van Cauwenberg et al., 2018).

Deviation:

The methodological quality of included papers was assessed using a scale proposed by Cerin and colleagues [45-49], as in previous neighbourhood environment research [50, 51].

(3)

Registered in the protocol:

Subgroups: male and female; children and adolescents; self-reported physical activity and device-measured physical activity.

Deviation:

Subgroups: children, adolescents, and mixed-age group; active travel, non-type-specific physical activity, active independent mobility, sports participation, and active outdoor play
