# Supplementary material for: Associations between parental perceptions of neighbourhood environment and physical activity in children and adolescents: a systematic review including 149 studies
Source: Int J Behav Nutr Phys Act. 2025 Jun 6;22:70. doi: 10.1186/s12966-025-01733-8 (PMC12143044; doi:10.1186/s12966-025-01733-8)
Supplement: Supplementary file 2 — Additional file 2. [file 12966_2025_1733_MOESM2_ESM.docx]

**Additional file 2. Search syntax**

**CINAHL, PsycInfo, and SPORTDiscus (via EBSCOhost)**

(parent* OR “care giver*” OR caregiver* OR family OR families OR mother* OR father* OR custodian* OR guardian*) AND (“built environment*” OR “physical environment*” OR street* OR connectivity OR walkab* OR neighbourhood* OR neighborhood* OR “green space*” OR greenspace* OR “community centre*” OR “community center*” OR park OR parks OR recreation* OR facility OR facilities OR footpath* OR “land use” OR “dwelling density” OR bike OR bicycle OR cycling OR outdoor* OR playground* OR infrastructure OR safety OR crime OR transport OR aesthetics OR “urban form” OR “urban design” OR stranger* OR "social environment" OR traffic OR “social cohesion” OR “social capital” OR “pedestrian infrastructure” OR “public open space” OR “playspace” OR “play space” OR “housing density” OR “residential density” OR “social risk” OR incivilit* OR “social norm” OR “social inequality” OR “sociocultural” OR “socio-cultural” OR “civic participation” OR “community participation” OR “concentrated disadvantage” OR “cultural norms” OR “social connectedness” OR “collective efficacy” OR “social control” OR violence OR “sense of place” OR “sense of belonging”) AND ("physical activit*" OR "physical inactivity" OR "motor activity" OR "energy expenditure" OR exercise OR "active play" OR walking OR running OR biking OR cycling OR "active transport" OR "active travel" OR "travel behavior" OR "travel behaviour" OR sport*) AND (perception* OR perceive* OR concern* OR fear* OR view* OR opinion* OR discernment* OR assessment* OR attitude*) AND (child* OR adolescen* OR youth OR “young people” OR student* OR pupil* OR kids OR teen*)

**Embase**

(parent* OR “care giver*” OR caregiver* OR family OR families OR mother* OR father* OR custodian* OR guardian*) AND (“built environment*” OR “physical environment*” OR street* OR connectivity OR walkab* OR neighbourhood* OR neighborhood* OR “green space*” OR greenspace* OR “community centre*” OR “community center*” OR park OR parks OR recreation* OR facility OR facilities OR footpath* OR “land use” OR “dwelling density” OR bike OR bicycle OR cycling OR outdoor* OR playground* OR infrastructure OR safety OR crime OR transport OR aesthetics OR “urban form” OR “urban design” OR stranger* OR "social environment" OR traffic OR “social cohesion” OR “social capital” OR “pedestrian infrastructure” OR “public open space” OR “playspace” OR “play space” OR “housing density” OR “residential density” OR “social risk” OR incivilit* OR “social norm” OR “social inequality” OR “sociocultural” OR “socio-cultural” OR “civic participation” OR “community participation” OR “concentrated disadvantage” OR “cultural norms” OR “social connectedness” OR “collective efficacy” OR “social control” OR violence OR “sense of place” OR “sense of belonging”) AND ("physical activit*" OR "physical inactivity" OR "motor activity" OR "energy expenditure" OR exercise OR "active play" OR walking OR running OR biking OR cycling OR "active transport" OR "active travel" OR "travel behavior" OR "travel behaviour" OR sport*) AND (perception* OR perceive* OR concern* OR fear* OR view* OR opinion* OR discernment* OR assessment* OR attitude*) AND (child* OR adolescen* OR youth OR “young people” OR student* OR pupil* OR kids OR teen*)

[**Environmental Science**](https://www.proquest.com/esdb?accountid=14844) **(via ProQuest)**

If((parent* OR "care giver*" OR caregiver* OR family OR families OR mother* OR father* OR custodian* OR guardian*) AND ("built environment*" OR "physical environment*" OR street* OR connectivity OR walkab* OR neighbourhood* OR neighborhood* OR "green space*" OR greenspace* OR "community centre*" OR "community center*" OR park OR parks OR recreation* OR facility OR facilities OR footpath* OR “land use” OR “dwelling density” OR bike OR bicycle OR cycling OR outdoor* OR playground* OR infrastructure OR safety OR crime OR transport OR aesthetics OR “urban form” OR “urban design” OR stranger* OR "social environment" OR traffic OR “social cohesion” OR “social capital” OR “pedestrian infrastructure” OR “public open space” OR “playspace” OR “play space” OR “housing density” OR “residential density” OR “social risk” OR incivilit* OR “social norm” OR “social inequality” OR “sociocultural” OR “socio-cultural” OR “civic participation” OR “community participation” OR “concentrated disadvantage” OR “cultural norms” OR “social connectedness” OR “collective efficacy” OR “social control” OR violence OR “sense of place” OR “sense of belonging”) AND ("physical activit*" OR "physical inactivity" OR "motor activity" OR "energy expenditure" OR exercise OR "active play" OR walking OR running OR biking OR cycling OR "active transport" OR "active travel" OR "travel behavior" OR "travel behaviour" OR sport*) AND (perception* OR perceive* OR concern* OR fear* OR view* OR opinion* OR discernment* OR assessment* OR attitude*) AND (child* OR adolescen* OR youth OR “young people” OR student* OR pupil* OR kids OR teen*)) OR abstract((parent* OR "care giver*" OR caregiver* OR family OR families OR mother* OR father* OR custodian* OR guardian*) AND ("built environment*" OR "physical environment*" OR street* OR connectivity OR walkab* OR neighbourhood* OR neighborhood* OR "green space*" OR greenspace* OR "community centre*" OR "community center*" OR park OR parks OR recreation* OR facility OR facilities OR footpath* OR “land use” OR “dwelling density” OR bike OR bicycle OR cycling OR outdoor* OR playground* OR infrastructure OR safety OR crime OR transport OR aesthetics OR “urban form” OR “urban design” OR stranger* OR "social environment" OR traffic OR “social cohesion” OR “social capital” OR “pedestrian infrastructure” OR “public open space” OR “playspace” OR “play space” OR “housing density” OR “residential density” OR “social risk” OR incivilit* OR “social norm” OR “social inequality” OR “sociocultural” OR “socio-cultural” OR “civic participation” OR “community participation” OR “concentrated disadvantage” OR “cultural norms” OR “social connectedness” OR “collective efficacy” OR “social control” OR violence OR “sense of place” OR “sense of belonging”) AND ("physical activit*" OR "physical inactivity" OR "motor activity" OR "energy expenditure" OR exercise OR "active play" OR walking OR running OR biking OR cycling OR "active transport" OR "active travel" OR "travel behavior" OR "travel behaviour" OR sport*) AND (perception* OR perceive* OR concern* OR fear* OR view* OR opinion* OR discernment* OR assessment* OR attitude*) AND (child* OR adolescen* OR youth OR “young people” OR student* OR pupil* OR kids OR teen*)) OR title((parent* OR "care giver*" OR caregiver* OR family OR families OR mother* OR father* OR custodian* OR guardian*) AND ("built environment*" OR "physical environment*" OR street* OR connectivity OR walkab* OR neighbourhood* OR neighborhood* OR "green space*" OR greenspace* OR "community centre*" OR "community center*" OR park OR parks OR recreation* OR facility OR facilities OR footpath* OR “land use” OR “dwelling density” OR bike OR bicycle OR cycling OR outdoor* OR playground* OR infrastructure OR safety OR crime OR transport OR aesthetics OR “urban form” OR “urban design” OR stranger* OR "social environment" OR traffic OR “social cohesion” OR “social capital” OR “pedestrian infrastructure” OR “public open space” OR “playspace” OR “play space” OR “housing density” OR “residential density” OR “social risk” OR incivilit* OR “social norm” OR “social inequality” OR “sociocultural” OR “socio-cultural” OR “civic participation” OR “community participation” OR “concentrated disadvantage” OR “cultural norms” OR “social connectedness” OR “collective efficacy” OR “social control” OR violence OR “sense of place” OR “sense of belonging”) AND ("physical activit*" OR "physical inactivity" OR "motor activity" OR "energy expenditure" OR exercise OR "active play" OR walking OR running OR biking OR cycling OR "active transport" OR "active travel" OR "travel behavior" OR "travel behaviour" OR sport*) AND (perception* OR perceive* OR concern* OR fear* OR view* OR opinion* OR discernment* OR assessment* OR attitude*) AND (child* OR adolescen* OR youth OR “young people” OR student* OR pupil* OR kids OR teen*))

**MEDLINE/PubMed**

(parent*[TW] OR "care giver*"[TW] OR caregiver*[TW] OR family[TW] OR families[TW] OR mother*[TW] OR father*[TW] OR custodian*[TW] OR guardian*[TW]) AND ("built environment*"[TW] OR "physical environment*"[TW] OR street*[TW] OR connectivity[TW] OR walkab*[TW] OR neighbourhood*[TW] OR neighborhood*[TW] OR "green space*"[TW] OR greenspace*[TW] OR "community centre*"[TW] OR "community center*"[TW] OR park[TW] OR parks[TW] OR recreation*[TW] OR facility[TW] OR facilities[TW] OR footpath*[TW] OR "land use"[TW] OR "dwelling density"[TW] OR bike[TW] OR bicycle[TW] OR cycling[TW] OR outdoor*[TW] OR playground*[TW] OR infrastructure[TW] OR safety[TW] OR crime[TW] OR transport[TW] OR aesthetics[TW] OR "urban form"[TW] OR "urban design"[TW] OR stranger*[TW] OR "social environment"[TW] OR traffic[TW] OR "social cohesion"[TW] OR "social capital"[TW] OR "collective efficacy"[TW] OR "pedestrian infrastructure"[TW] OR "public open space"[TW] OR "playspace"[TW] OR "play space"[TW] OR "housing density"[TW] OR "residential density" OR "social risk"[TW] OR incivilit*[TW] OR "social norm"[TW] OR "social inequality"[TW] OR "sociocultural"[TW] OR "socio-cultural"[TW] OR "civic participation"[TW] OR "community participation"[TW] OR "concentrated disadvantage"[TW] OR "cultural norms"[TW] OR "social connectedness"[TW] OR "social control"[TW] OR violence[TW] OR "sense of place"[TW] OR "sense of belonging"[TW]) AND ("physical activit*"[TW] OR "physical inactivity"[TW] OR "motor activity"[TW] OR "energy expenditure"[TW] OR exercise[TW] OR "active play"[TW] OR walking[TW] OR running[TW] OR biking[TW] OR cycling[TW] OR "active transport"[TW] OR "active travel"[TW] OR "travel behavior"[TW] OR "travel behaviour"[TW] OR sport*[TW]) AND (perception*[TW] OR perceive*[TW] OR concern*[TW] OR fear*[TW] OR view*[TW] OR opinion*[TW] OR discernment*[TW] OR assessment*[TW] OR attitude*[TW]) AND (child*[TW] OR adolescen*[TW] OR youth[TW] OR "young people"[TW] OR student*[TW] OR pupil*[TW] OR kids[TW] OR teen*[TW])

**Scopus**

TITLE-ABS-KEY(parent* OR "care giver*" OR caregiver* OR family OR families OR mother* OR father* OR custodian* OR guardian*) AND TITLE-ABS-KEY("built environment*" OR "physical environment*" OR street* OR connectivity OR walkab* OR neighbourhood* OR neighborhood* OR "green space*" OR greenspace* OR "community centre*" OR "community center*" OR park OR parks OR recreation* OR facility OR facilities OR footpath* OR "land use" OR "dwelling density" OR bike OR bicycle OR cycling OR outdoor* OR playground* OR infrastructure OR safety OR crime OR transport OR aesthetics OR "urban form" OR "urban design" OR stranger* OR "social environment" OR traffic OR "social cohesion" OR "social capital" OR "pedestrian infrastructure" OR "public open space" OR "playspace" OR "play space" OR "housing density" OR "residential density" OR "social risk" OR incivilit* OR "social norm" OR "social inequality" OR "sociocultural" OR "socio-cultural" OR "civic participation" OR "community participation" OR "concentrated disadvantage" OR "cultural norms" OR "social connectedness" OR "collective efficacy" OR "social control" OR violence OR "sense of place" OR "sense of belonging") AND TITLE-ABS-KEY("physical activit*" OR "physical inactivity" OR "motor activity" OR "energy expenditure" OR exercise OR "active play" OR walking OR running OR biking OR cycling OR "active transport" OR "active travel" OR "travel behavior" OR "travel behaviour" OR sport*) AND TITLE-ABS-KEY(perception* OR perceive* OR concern* OR fear* OR view* OR opinion* OR discernment* OR assessment* OR attitude*) AND TITLE-ABS-KEY(child* OR adolescen* OR youth OR "young people" OR student* OR pupil* OR kids OR teen*)

**Transportation Research Information Services (TRIS)**

(parent* OR “care giver*” OR caregiver* OR family OR families OR mother* OR father* OR custodian* OR guardian*) AND (“built environment*” OR “physical environment*” OR street* OR connectivity OR walkab* OR neighbourhood* OR neighborhood* OR “green space*” OR greenspace* OR “community centre*” OR “community center*” OR park OR parks OR recreation* OR facility OR facilities OR footpath* OR “land use” OR “dwelling density” OR bike OR bicycle OR cycling OR outdoor* OR playground* OR infrastructure OR safety OR crime OR transport OR aesthetics OR “urban form” OR “urban design” OR stranger* OR "social environment" OR traffic OR “social cohesion” OR “social capital” OR “pedestrian infrastructure” OR “public open space” OR “playspaces” OR “play space” OR “housing density” OR “residential density” OR “social risk” OR incivilit* OR “social norm” OR “social inequality” OR “sociocultural” OR “socio-cultural” OR “civic participation” OR “community participation” OR “concentrated disadvantage” OR “cultural norms” OR “social connectedness” OR “collective efficacy” OR “social control” OR violence OR “sense of place” OR “sense of belonging”) AND ("physical activit*" OR "physical inactivity" OR "motor activity" OR "energy expenditure" OR exercise OR "active play" OR walking OR running OR biking OR cycling OR "active transport" OR "active travel" OR "travel behavior" OR "travel behaviour" OR sport*) AND (perception* OR perceive* OR concern* OR fear* OR view* OR opinion* OR discernment* OR assessment* OR attitude*) AND (child* OR adolescen* OR youth OR “young people” OR student* OR pupil* OR kids OR teen*)

**Web of Science Core Collection**

TS=((parent* OR “care giver*” OR caregiver* OR family OR families OR mother* OR father* OR custodian* OR guardian*) AND (“built environment*” OR “physical environment*” OR street* OR connectivity OR walkab* OR neighbourhood* OR neighborhood* OR “green space*” OR greenspace* OR “community centre*” OR “community center*” OR park OR parks OR recreation* OR facility OR facilities OR footpath* OR “land use” OR “dwelling density” OR bike OR bicycle OR cycling OR outdoor* OR playground* OR infrastructure OR safety OR crime OR transport OR aesthetics OR “urban form” OR “urban design” OR stranger* OR "social environment" OR traffic OR “social cohesion” OR “social capital” OR “pedestrian infrastructure” OR “public open space” OR “playspaces” OR “play space” OR “housing density” OR “residential density” OR “social risk” OR incivilit* OR “social norm” OR “social inequality” OR “sociocultural” OR “socio-cultural” OR “civic participation” OR “community participation” OR “concentrated disadvantage” OR “cultural norms” OR “social connectedness” OR “collective efficacy” OR “social control” OR violence OR “sense of place” OR “sense of belonging”) AND (“physical activit*” OR "physical inactivity" OR "motor activity" OR "energy expenditure" OR exercise OR "active play" OR walking OR running OR biking OR cycling OR "active transport" OR "active travel" OR "travel behavior" OR "travel behaviour" OR sport*) AND (perception* OR perceive* OR concern* OR fear* OR view* OR opinion* OR discernment* OR assessment* OR attitude*) AND (child* OR adolescen* OR youth OR “young people” OR student* OR pupil* OR kids OR teen*))
