# Supplementary material for: Associations between parental perceptions of neighbourhood environment and physical activity in children and adolescents: a systematic review including 149 studies
Source: Int J Behav Nutr Phys Act. 2025 Jun 6;22:70. doi: 10.1186/s12966-025-01733-8 (PMC12143044; doi:10.1186/s12966-025-01733-8)
Supplement: Supplementary file 3 — Additional file 3. [file 12966_2025_1733_MOESM3_ESM.docx]

**Criteria for quality of evidence assessment**

**1. Starting quality of evidence**

Study design

- Evidence coming mostly from observational studies will initially be rated as “low quality”
- Evidence coming mostly from quasi experimental will initially be rated as “high quality”

**2. Indications for downgrading quality of evidence**

Risk of bias

- Large representation of studies of low methodological quality, as assessed using the above-mentioned scale [45-49]
- The findings of studies with moderate or high methodological quality are largely different from the findings of studies with low methodological quality (e.g. three studies of low quality found significant positive associations, while two studies of moderate quality reported non-significant associations)

Inconsistency of results

- Mixture of studies with significant positive (“+”) and significant negative (“-“) results (e.g. three studies found a positive association and two studies found a negative association)

Indirectness of evidence

- Large representation of studies conducted mostly among gender-specific (parent or child) samples (e.g. two out of three studies that found a significant positive association were conducted among girls)
- Large representation of studies conducted in countries with a given national income level (e.g. high-income countries)

Imprecision

- The pooled sample size is smaller than 300

Publication bias

- Disproportionally large representation of observational study designs (vs. experimental study designs) among studies that found significant associations, compared to studies that found non-significant associations
- Disproportionally large representation of small sample sizes (vs. large sample sizes) among studies that found significant associations, compared to studies that found non-significant associations
- Disproportionally large representation of older/early (vs. more recent) studies among studies that found significant associations, compared to studies that found non-significant associations

**3. Indications for upgrading quality of evidence**

- None applicable
