# Supplementary material for: Associations between parental perceptions of neighbourhood environment and physical activity in children and adolescents: a systematic review including 149 studies
Source: Int J Behav Nutr Phys Act. 2025 Jun 6;22:70. doi: 10.1186/s12966-025-01733-8 (PMC12143044; doi:10.1186/s12966-025-01733-8)
Supplement: Supplementary file 4 — Additional file 4. [file 12966_2025_1733_MOESM4_ESM.docx]

**Additional file 4. Characteristics of included papers**

| **Author, year** | **Language of the full-text** | **Study design** | **Country/**  **region** | **Study area** | **Response rate %** | **Sample type** | **Project** | **Sample size** | **Age** | **Data collection year** | **Female %** | **Parent respondents (female %)** | **Measurement** | **Domain/intensity/type of PA** | **Measure** | **Validated and reliable (Yes/No)** | **Questionnaire type** | **Attributes -Parental perceptions of neighbourhood environment** | **Validated and reliable (Yes/No)** | **Statistical Analyses** | **Adjustment for confounders** |
| --- | --- | --- | --- | --- | --- | --- | --- | --- | --- | --- | --- | --- | --- | --- | --- | --- | --- | --- | --- | --- | --- |
| Adkins et al. 2004 [52] | English | cross-sectional | US | Minneapolis/St. Paul | NR | clinic school visits | National Heart, Lung, and Blood Institute-sponsored GEMS | 52 | 8-10 years old | NR | 100 | 90 | device - CSA (computer Science Application) | non-type-specific physical activity: MVPA out of school | average daily minutes of MVPA from 12pm to 6pm | Yes | GEMS psychosocial survey and Family Environment Scale | neighbourhood safety; access to facilities for PA; cohesion | Yes | Pearson correlation coefficients | No |
| Aliyas 2022 [41] | English | cross-sectional | Iran | Bandar Abbas (medium-sized) | NR | random schools selected | NR | 1041 | 7-12 years old | 2019-2020 | 58 | 79.2 | questionnaire (self-reported) | non-type-specific physical activity: MVPA, VPA (non-active and active) | frequency of MVPA/VPA for more than 60 minutes/VPA per week | Yes | adapted questionnaire from previous studies | personal and traffic safety; physical and social disorders; safe to play | Yes | binary logistic regression | parent's gender, parent's job, children's age, parents’ education and economic status |
| Aliyas et al. 2022 [53] | English | cross-sectional | Iran | Bandar Abbas (medium-sized) | NR | two neighbourhoods from each socio-economic level areas (low and high) were randomly selected, and two public schools in each neighbourhood participated | NR | 1001 | 7-12 years old | 2019-2021 | 60 | NR | questionnaire (self-reported) | active travel: school travel | active (walking) and passive (private car, carpool and public bus) | No | Safe Routes to School Program | physical barriers, crime safety, traffic safety; collective efficacy | Yes | partial least square structural equation models | parent's gender, parents' job, children's age, parents’ education and economic status |
| Allen and Vella 2015 [27] | English | longitudinal^a^ | Australia | nation-wide | 20.6 | nationally representative sample | Longitudinal Study of Australian Children (LSAC) Kindergarten cohort at wave 1 and wave 5 | 3956 | 12-13 years old | 2012 | 48.9 | NR | questionnaire (parent reported) | sports participation | the number of days per week * the average number of hours | No | NR | Neighbourhood remoteness; belonging, safety; facilities; and public transport availability | No | multiple regression | sex, indigenous status, language spoken at home, pubertal status, child BMI, general health |
| Appelhans and Li 2016 [54] | English | cross-sectional | US | Chicago | NR | NR | NR | 88 | 6-13 years old | 2012-2013 | 53 | NR | device - ActiGraph GT3X and questionnaire (parent reported) | non-type-specific physical activity: MVPA. sports participation: unstructured active play | total minutes of MVPA; organized sports and unstructured active play (how many days) | Yes | NEWS and NEWS-Y | neighbourhood safety; neighbourhood PA venues | Yes | ordinal logistic regression | poverty to-income ratio, child gender, age, and wight status |
| Aranda-Balboa et al. 2021 [55] | English | cross-sectional | Spain | Granada, Jaén, Toledo and Valencia | 66.83 | randomly selected | Pedalea y Anda al Cole/Cycle andWalk to School (PACO) Study | 401 | 13.26±1.78 years old | 2018-2020 | NR | NR | questionnaire (self-reported) | active travel: active or passive | “mode and frequency of commuting to and from school questionnaire”: The questions were: “How do you usually get to school?” and “How do you usually get home from school?”, and the possible answers were walking, cycling, car, motorbike, scholar bus, public bus, metro/train or other; only one option could be chosen. | Yes | Parental Perception of Barriers Towards Active Commuting to School (PABACS) | The question was formulated in this way: “Here are some situations that might occur on a day-to-day basis. For each situation, please indicate how much you agree or disagree that it might affect your decision not to allow your child to walk/bike to or from school. (Please check only one option for each question.)”. The scale includes 23 different items categorized as general barriers, including those common to both walking and cycling to school (e.g., There is a long distance from home to school), walking barriers, including those referring to walking (e.g., there are no sidewalks or they are in poor condition), and cycling barriers, including those referring to cycling (e.g., there is no bike path or it is in poor condition). The scale asked the participants to rate how strongly they agreed with each statement through a Likert scale of 4 points (from “Nothing” to “Substantially”). | Yes | binary logistic regression | NR |
| Babey et al. 2008 [57] | English | cross-sectional | US | California | NR | representative | NR | 3251 | 12-17 years old | 2003 | NR | NR | questionnaire (self-reported) | physical activity | Regular activity was defined as either at least 20 minutes of vigorous activity on 3 or more of the last 7 days or at least 30 minutes of moderate activity on 5 or more of the last 7 days. | No | NR | parental perception of neighbourhood safety | No | stratified logistic regressions | age, gender, race/ethnicity, urbanicity, housing type, and family income |
| Babey et al. 2009 [56] | English | cross-sectional | US | California | 48.5 | random sample | California Health Interview Survey 2005 | 3983 | 12-17 years old | 2005 | 49 | NR | questionnaire (self-reported) | active travel: active travel to/from school | the number of days per week (walk, bicycle, or skateboard) | Yes | NR | neighbourhood safety | No | logistic regressions | adolescents' age, gender, race, parental household income, household address, parental walking for transportation |
| Barnett et al. 2019 [59] | English | cross-sectional | Hong Kong | Hong Kong | 68 | stratified by census-based medium household income and transport-related walkability | IPEN (International Physical Activity and the Environment Network) Hong Kong | 1299 | 14.70 ± 1.57 years old | 2013-2015 | 57.04 | NR | questionnaire (self-reported) | active travel: active transport modes to/from school | frequency of transport modes/both to and from school [0 day; 1-4 days; 5 days] | Yes | NEWS-Y | proximity to school; proximity of commercial facilities to home; proximity of nearest transit stop to home; proximity of recreational PA facility to home; proximity of food outlets (restaurants/food stores) to home; proximity to destinations (composite index); barriers to walking in the neighbourhood; neighbourhood street connectivity; neighbourhood residential density; neighbourhood traffic safety; neighbourhood safety from crime; neighbourhood aesthetic; access to services; pedestrian infrastructure in the neighbourhood | Yes | generalised linear models and zero-inflated negative binomial models with robust standard errors accounting for TPU- and school-level clustering | adolescent gender and age, social desirability, parent-reported neighbourhood self-selection and length of residence, number of children in the household, highest education level in the household, number of motorised vehicles in the household, neighbourhood-level socio-economic status and monthly household income. |
| Barnett et al. 2019 [58] | English | cross-sectional | Hong Kong | Hong Kong | 68 | stratified by census-based medium household income and transport-related walkability | IPEN (International Physical Activity and the Environment Network) Hong Kong | 1299 | 14.70 ± 1.57 years old | 2013-2015 | 57.04 | NR | questionnaire (self-reported) | active travel: active transport modes to/from school | frequency of transport modes/both to and from school [0 day; 1-4 days; 5 days] * weekly time spent in ATS | Yes | NEWS-Y | proximity to school; proximity of commercial facilities to home; proximity of nearest transit stop to home; proximity of recreational PA facility to home; proximity of food outlets (restaurants/food stores) to home; proximity to destinations (composite index); barriers to walking in the neighbourhood; neighbourhood street connectivity; neighbourhood residential density; neighbourhood traffic safety; neighbourhood safety from crime; neighbourhood aesthetic; access to services; pedestrian infrastructure in the neighbourhood | Yes | hierarchical regression analyses | adolescent gender and age, social desirability, parent-reported neighbourhood self-selection and length of residence, number of children in the household, highest education level in the household, number of motorised vehicles in the household, neighbourhood-level socio-economic status and monthly household income, adolescent-reported social desirability, parents self-selecting a neighbourhood likely to encourage adolescent PA and a two-category school classification (public or aided (referred to as public) and private or international (referred to as private)). |
| Baskin et al. 2013 [60] | English | cross-sectional | US | Birmingham, Alabama | NR | NR | NR | 116 | 12-16 years old | 2011 | 53.44 | NR | device - ActiGraph GT1M | non-type-specific physical activity: MVPA | daily minutes of MVPA for the total week, for weekday, for weekend and for daily weekly MVPA by sex | Yes | safety subscale | perceived safety | No | multivariate logistic regression models | adolescent's age, parent education, parent marital status, weigh status, media equipment in home, family social support, adolescent self-efficacy |
| Beets and Foley 2007 [61] | English | cross-sectional | US | nation-wide | NR | nationally representative sample; multistage cluster sampling design | Early Childhood Longitudinal Study - Kindergarten Cohort 1998-1999 (ECSL-K) | 10,694 | 5-6 years old | 1998 | 48.99 | 75 | questionnaire (parent reported) | non-type-specific physical activity: MVPA | days per week of vigorous PA (0-7) | No | NR | neighbourhood quality: [violent crimes (e.g. drive-by shootings); garbage (litter or broken glass in the street or road, on the sidewalks, or in yards); vacant houses and/or buildings; drugs (selling or using drugs) or excessive drinking in public; and burglaries or robberies] neighbourhood safety is a mediator | No | multilevel structural equation modelling | child weight status, motor skills, ethnicity, and television viewing |
| Bell et al. 2020 [62] | English | cross-sectional | Australia | Victoria | 81 | randomly selected suburbs | Resilience for Eating and Activity Despite Inequality (READI) and the Active Independent Mobility (AIM) studies | 289 | 8-15 years old | 2007 | 54 | 100 | questionnaire (parent reported) | active travel: cycling | frequency and duration of their child's cycling to/from school, for travel and for fun on weekends and weekdays in a usual week [Frequency ≥ once/wk (Ref: <once/wk) and Duration ≥60 minutes wk ((Ref: <60 minutes/wk)] | No | NR | neighbourhood level variables satisfied - easy for children to cycle in neighbourhood (ref ‘disagree’); satisfied with number of pedestrian crossings; can ride to local shops; There are major barriers to walking/cycling; child would have to cross several roads to get to play areas; child has to cross busy road(s) to get to areas to play | No | multivariable logistic regression analyses | child’s age, sex, rural/urban location, maternal education and distance between home and school |
| Brewer and Kimbro 2014 [63] | English | cross-sectional | US | nation-wide | NR | nationally representative sample | Early Childhood Longitudinal Study - Kindergarten Cohort 1998-1999 (ECSL-K) | 17,510 | 5-7 years old | 1998-1999 | 42 | 100 | questionnaire (parent reported) | non-type-specific physical activity: MVPA | days per week of vigorous PA (0-7) | No | NR | neighbourhood safety for playing outside; neighbourhood disorder index (garbage, litter and other problems) | No | zero-inflated Poisson regression | race/ethnicity, child's gender, mother's age, child's age, child's health, FPL, mother's education level, number of siblings, family type, physical education class |
| Bringolf-Isler et al. 2008 [64] | English | cross-sectional | Switzerland | Berne, Biel-Bienne, Payerne | 65 | representative sample | NR | 1345 | 6-14 years old | 2004-2005 | 51.2 | NR | questionnaire (parent reported) | active travel: active transportation modes | walking; bicycling; reference category inactive transportation | No | NR | perceived safety on the way to school | No | multivariate models | child’s height and weight, regular day care attendance after school and the number of cars in the household |
| Bringolf-Isler et al. 2010 [65] | English | cross-sectional | Switzerland | Berne, Biel-Bienne, Payerne | 65 | representative sample | SCARPOL | 1081 | 6-14 years old | 2004-2006 | 50.87 | NR | questionnaire (parent reported) | active outdoor play: vigorously outdoor play | zero minutes, <30 minutes, 30 minutes to one hour, one to two hours, two to four hours, and >4 hours. | Yes | NR | traffic; no garden/green space available; crime | No | multivariable regression models | sex, age, maternal education, community, nationality, number of younger siblings, and day care. |
| Bringolf-Isler et al. 2019 [66] | English | cross-sectional | Switzerland | nation-wide | 64.27 | representative sample | SOPHYA | 1306 | 6-16 years old | 2013-2015 | 48.6 | NR | device - ActiGraph GT1M or GT3X & cycling | non-type-specific physical activity: MVPA. Active travel: cycling duration | MVPA in minutes/day; hours/week in cycling | Yes | Australian CLAN study questionnaire (Children Living in Active Neighbourhoods) | road safety, incivilities and personal safety of their children | Yes | linear regression models | age, sex, household income, SEP, language region, urbanicity and season, accelerometer wearing time and device (GT1M versus GT3x) |
| Buck et al. 2015 [67] | English | cross-sectional | German | Delmenhorst and Lower Saxony (only urban environment) | NR | NR | Identification and prevention of Dietary- and lifestyle-induced health Effects In Children and infantS study (IDEFICS) | 300 | 6-9.9 years old | 2007-2008 | 54.3 | NR | device - ActiGraph | non-type-specific physical activity: MVPA | MVPA in minute/day | Yes | NR | perceived safety | No | basic models | age, body mass index (BMI), education and safety concerns of parents, season and valid wear time of accelerometers. |
| Buliung et al. 2017 [68] | English | cross-sectional | Canada | Toronto | NR | a large0scale interdisciplinary study | Built Environment and Active Transportation Research Project (BEAT) | 1035 | 9-13 years old | NR | 50 | NR | questionnaire (self-reported) | active IM | those who walk independently; and children who walk escorted by an adult | No | NR | busy streets to cross | No | binomial multivariate logistic regression modeling | age, gender, educational attainment from parents |
| Butte et al. 2014 [69] | English | longitudinal | US | San Francisco Bay | NR | randomly selected | NR | 282 | 8.9 ± 05 years old | 2007-2009 | 52.83 | 100% mother; 64% father (both) | device - ActiGraph | non-type-specific physical activity: MVPA | MVPA in minute/day | Yes | Disorder and Victimization subscales of the Neighbourhood Context Scale | neighbourhood disorder and neighbourhood victimization | Yes | longitudinal regression models; cross-lagged panel models | child gender, child age, maternal age, paternal age, maternal education, paternal education, household income, maternal Spanish-language acculturation, paternal Spanish-language acculturation, number of household members |
| Cadogan et al. 2014 [70] | English | cross-sectional | Republic of Ireland | nation-wide | 57 | two-stage clustered sampling method | Growing Up in Ireland (GUI) first wave 2008 | 8,568 | 9 years old | 2008 | 51.4 | 98 | questionnaire (parent reported) | non-type-specific physical activity: PA level | low, moderate, high | Yes | NR | safe places to play | No | multivariate multinomial logistic regression (forward block entry) | the study child’s gender, whether the study child was a member of a sports or fitness club (yes/no), total screen time, the nature of study child’s favourite hobby and the study child’s weight status, caregiver's weight, caregiver's education, siblings, household type |
| Carlson et al. 2014 [71] | English | cross-sectional | US | Baltimore, Maryland-Washington, DC and Seattle-King County, Washington metropolitan | NR | selected from census block representing high or low walkability and high or low income | Teen Environment and Neighborhood (TEAN) | 294 | 12-16 years old | 2009-2011 | NR | NR | questionnaire (self-reported) | active travel: active transport modes to/from school | The total number of active trips to and from school was split into three categories based on the distribution of the continuous variable, and so environmental correlates of occasional (vs no) and habitual (vs no) active travel to/from school could be compared. The categories were 0 trips to/from school, 1–4 trips to/from school (occasional) and 5–10 trips to/from school (habitual). | Yes | NEWS-Y | land use mix-diversity, land use mix-access, street connectivity, walking facilities, neighbourhood aesthetics, traffic safety, pedestrian safety and crime safety | Yes | mixed effects multinomial regression models | distance to school, child's age, gender, non-Hispanic Caucasian, parent with college degree, parent married or living with partner, parent works full-time, vehicles/driver in household |
| Carson et al. 2010 [72] | English | cross-sectional | US | Alberta | 88.5 | stratified into three geographic: urban; towns; municipalities (randomly selected schools) | Raising healthy Eating and Active Living Kids in Alberta (REAL Kids Alberta) | 3028 | 10-11 years old | NR | 52 | NR | questionnaire (self-reported and parent reported) | non-type-specific physical activity: PA. Active travel: active transport | Physical Activity Questionnaire for Older Children (PAQ-C) ("physically active" OR "non active") \| active transport (walking and biking) | Yes | NR | satisfaction/services (parks, playgrounds, places to play, good access to sports and recreation and access); safety for traffic and crime; sidewalks/parks | Yes | principal component analysis and fully adjusted regression models | gender, geographic region, household income, and parental education |
| Carver et al. 2005 [74] | English | cross-sectional | Australia | Sydney | 79.1 | NR | longitudinal Nepean Study (1996-1998) | 347 | 12-13 years old | 2002-2003 | 50.4 | NR | questionnaire (self-reported) | active travel: active transport | duration and frequency in walking for exercise, walking to/from school, | Yes | NR | good facilities for child to use; safe to ride/walk a bike; good places where my child can be physically active; so much traffic | Yes | multiple linear regressions | level of maternal education |
| Carver et al. 2008 [32] | English | cross-sectional | Australia | Melbourne | NR | recruited from various SES | Children Living in Active Neighbourhoods (CLAN) | 346 | 8-9 and 13-15 years old | 2004 | 51.5 | 85 | device - ActiGraph 7164 | non-type-specific physical activity: MVPA | MVPA in minute/day | Yes | NR | road safety, incivilities, personal safety | Yes | linear regression models | No |
| Carver et al. 2014 [76] | English | longitudinal^a^ | UK | Norfolk | 49 | randomly sampled | Sport, Physical activity and Eating behavior: Environmental Determinants in Young people (SPEEDY) | 491 | 9-10 years old | 2007-2008 | 60.7 | NR | questionnaire (self-reported) | active independent mobility | active IM or active travel with adults | No | ANEWS | sense of community score; the traffic makes it too dangerous for my child to walk/cycle to school; I am worried that something will happen to my child on the way to school; walkability score | Yes | multiple logistic regression | IM at T1, parental education level, distance to school, urban/rural location |
| Carver et al. 2015 [75] | English | cross-sectional | Australia | Victoria | 18.5 | randomly sampled | NR | 640 | 11.6 ± 2.0 years old | 2010 | 51 | NR | questionnaire (self-reported) | active travel: cycling | child cycling at least once per week | No | NR | traffic injury; social trust | No | a series of logistic regression | age, sex, enrolment at primary/secondary school and urban/rural location |
| Carver et al. 2023 [73] | English | longitudinal^a^ | US | San Diego County, California and King County | NR | stratified according to their supportiveness of PA | Neighborhood Impact on Kids (NIK) longitudinal study | 727 | 6-12 years old | 2009-2011 | 50.21 | 86 | device - ActiGraph GT1M | non-type-specific physical activity: MVPA | MVPA in minute/day | Yes | NEWS-Y | perceptions of safety | Yes | generalized additive mixed models | parent's age and gender, child's age, gender, and ethnicity; highest parental education, employment, marital status, number of children, number of vehicles, number of adults with driver's licenses, time in neighbourhood, neighbourhood self-selection, SES |
| Chillón et al. 2014 [78] | English | cross-sectional | US | five geographic regions (Alaska and Northwest (NW), California (CA), Southcentral (SC), Southeast (SE), and Northeast (NE)), | NR | nationwide study: 14 schools were randomly selected | National Evaluation of Walk to School (WTS) project | 10809 | 9-11 years old | 2003 | NR | NR | questionnaire (self-reported) | active travel: active school travel | total number of active trips per week | Yes | NR | They included external safety (6 items: bullies, kidnapping, arriving safely to school, unleashed dogs and traffic congestion), suitability of the route (6 items: lack of sidewalks and crosswalks, steep hills, areas without people around, speed and traffic and insufficient daylight in the morning) | No | generalized linear mixed models with log-link functions | distance to school |
| Cohen et al. 2017 [79] | English | randomised controlled trial | New Zealand | Newcastle | 78 | match-paired based on their size and SES | Supporting Children's Outcomes using Rewards, Exercise and Skills (SCORES) | 460 | 7-10 years old | 2012 | 54.1 | NR | device - ActiGraph GT3X+ | non-type-specific physical activity: MVPA | total MVPA | Yes | Children’s Leisure Activities Study Survey | perceived access to PA opportunities in the local community | Yes | multi-level linear analysis | sex, age, BMI-z score, SES and baseline PA values |
| Côté-Lussier et al. 2015 [80] | English | cross-sectional | Canada | Quebec | 48 | population-based sampling strategy | Quebec Adipose and Lifestyle Investigation in Youth (QUALITY) | 630 | 8-10 years old | 2005–2008 | 45.6 | NR | device - ActiGraph 7164 | non-type-specific physical activity: MVPA | daily total MVPA | Yes | NR | general safety: ‘is at high risk of crime’, ‘is attractive’ and ‘kids can play outside without danger’ | No | structural equation models | age, sex, and family income, mother's weight, BMI, child average daily caloric intake and average number of hours of sleep per night |
| Crawford et al. 2010 [81] | English | longitudinal | Australia | Melbourne | 44 | stratified random sampling proportionate to school size | Children Living in Active Neighbourhoods (CLAN) | 314 | 10-12 years old | 2001, 2004, 2006 | 57.475 | NR | device - ActiGraph 7164 | non-type-specific physical activity: MVPA | daily total MVPA | Yes | NR | local traffic, road safety, sporting venues, public transport, no lights/crossings; busy road | Yes | generalized estimating equations analysis | maternal education, parental marital status, siblings, BMI z score |
| Curriero et al. 2013 [82] | English | cross-sectional | US | Baltimore City | NR | NR | Neighborhood Inventory for Environmental Typology (NIfETy) and Multiple Opportunities to Reach Excellence (MORE) Project | 362 | 8-12 years old | 2007 | 54 | NR | questionnaire (self-reported) | active travel: walking to school | Yes (walking to school) or otherwise | No | NR | neighborhood is safe (parent perception) | No | multivariate logistic regression model | gender, age, education, and clustering within Neighborhood Statistical Area (NSA) |
| Cutumisu et al. 2014 [83] | English | cross-sectional | Canada | Quebec | NR | random digit dialling telephone | NR | 809 | 9-13 years old | 2012 | 49.1 | NR | questionnaire (self-reported and parents) | active travel: active transportation to school | child: mode choice; parent: how many days per week walking or biking | No | NR | neighbourhood safety | No | logistical regression models | child's age and sex; child's impairment, child's school level; parent's sex, age, education, family income, material deprivation index, social deprivation index |
| Datar et al. 2013 [88] | English | longitudinal^a^ | US | nationally representative cohort | 43.9 | sampled within primary sampling units, nationally representative | Early Childhood Longitudinal Study - Kindergarten Cohort 1998-1999 (ECSL-K) | 8300 | 5-13 years old | 1999-2007 | NR | NR | questionnaire (parent reported) | non-type-specific physical activity: MVPA | days per week of vigorous PA (0-7) | No | NR | neighbourhood safety | No | linear regression models | age in months, gender, race/ethnicity (non-Hispanic white, non-Hispanic black, Asian, Hispanic, and other race or multiacre), mother’s education (less than high school, high school graduate or equivalent, some college, and college graduate or higher), annual household income (in categories), urbanicity (urban area, city, town), single-parent household, and number of siblings in the household. |
| Datar et al. 2015 [89] | English | cross-sectional^b^ | US | 12 army installations | 24.65 | state-level childhood obesity rates | Military Teenagers Environment Exercise and Nutrition Study (MTEENS) | 903 | 12-13 years old | 2013 | NR | NR | questionnaire (self-reported) | non-type-specific physical activity: MVPA | MPA in minute/day; VPA | No | NEWS-Y | land use mix-diversity, street connectivity, walking/cycling facilities, neighbourhood aesthetics, traffic/pedestrian safety, crime safety, recreational facilities, residential density, land-use mix-access | Yes | multivariate regression models | age, gender, race/ethnicity, parents' marital status, both parents' education levels, military parents rank; household income, number of adults and children in the household, on-post residence and time at current installation. |
| Davidson et al. 2010 [90] | English | cross-sectional | Canada | Alberta | 57.94 | stratified random sampling design | Raising healthy Eating and Active Living Kids in Alberta (REAL Kids Alberta) | 3421 | 10-11 years old | 2008 | NR | NR | questionnaire (self-reported and parents) | non-type-specific physical activity: MVPA | [PAQ-C] Parents and students responded to activity questions on: (a) travel to and from school; (b) time spent to get to and from school; (c) frequency of child’s activities outside of school hours; (d) activities at morning and lunch recess in the past seven days; and (e) frequency of involvement in sports and physical activities in the past seven days. | Yes | NR | neighbourhood satisfaction and services; neighbourhood safety; neighbourhood playgrounds and parks | No | multi-level logistic regression | child’s gender, household income, parental education and whether they resided urban, in towns or rurally |
| Davison et al. 2012 [91] | English | cross-sectional | US | New York | NR | randomly selected | Upstate Health and Wellness Survey 2009 | 355 | 6-12 years old | 2009 | 49.9 | 55.5 | questionnaire (parent reported) | days of PA | days per week of recommended levels of PA (i.e. 60 minutes of PA per day). | Yes | National Survey of Child Health | social capital | Yes | structuaral equation models | child's age, household education, participation in a food assistance program |
| De Meester et al. 2014 [92] | English | cross-sectional | Belgium | Flanders | 76.7 | randomly selected | NR | 736 | 10-12 years old | 2010-2011 | 48.1 | NR | device - ActiGraph GT1M and questionnaire (self-reported) | active travel: active transport to/from school; walking for transport during leisure time; cycling for transport during leisure time. Non-type-specific physical activity: total PA level and daily number of step counts | minutes of all activities from Flemish Physical Activity Questionnaire (FPAQ) | Yes | NEWS-Y | land use mix-diversity, street connectivity, walking/cycling facilities, neighbourhood aesthetics, traffic/pedestrian safety, crime safety, recreational facilities, residential density, land-use mix-access, distance to school, maintenance and quality of walking and cycling infrastructure | Yes | multiple linear regression | educational attainment of mother and father, type of monitor (pedometer or accelerometer). |
| DeWeese et al. 2013 [94] | English | cross-sectional | US | low-income New Jersey cities | 49 | randomly selected | NR | 765 | 6-11 years old | 2009-2010 | 50 | NR | questionnaire (parent reported) | active travel: active travel to/from school | days | Yes | NR | safety from traffic and crime; unpleasantness for walking | No | multivariate logistic regression | student's age, gender, and race; mother's education; family income; number of years the parent has resided in the U.S. |
| DeWeese et al. 2022 [93] | English | longitudinal | US | low-income New Jersey cities | NR | Survey Sampling | New Jersey Child Health Study (HJCHS) | 383 | 6-15 years old | 2009-2017 | 46.5 | NR | questionnaire (parent reported) | active travel: active travel to/from school | days | Yes | NR | safety from traffic; safety from crime; unpleasantness for walking | No | multivariate logistic regression | child age, sex, and race/ethnicity; parent’s education and nativity status; household poverty level; car availability; neighbourhood level characteristics; distance from home to school |
| D'Haese et al. 2011 [85] | English | cross-sectional | Belgium | Flanders | 69.9 | randomly selected | Ghent University Special Research Fund (BOF) BOF08/24J/134 | 696 | 11-12 years old | NR | NR | NR | questionnaire (parent reported) | active travel: active travel to/from school | modes and the number of minutes from Flemish Physical Activity Questionnaire (FPAQ) | Yes | NEWS-Y | street connectivity, walking/cycling facilities, neighbourhood aesthetics, traffic/pedestrian safety, crime safety, residential density, land-use mix-access, accessibility | Yes | two-level logistic regressions | gender, SES, household distance from school |
| D'Haese et al. 2013 [86] | English | cross-sectional | Australia | Melbourne | 44 | stratified random sampling | Children Living in Active Neighbourhoods (CLAN) | 929 | 10-12 years old | 2003 | 53.9 | 82 | device - ActiGraph 7164 | non-type-specific physical activity: MVPA | average minutes/day outside school hours | Yes | previous studies used: (Carver et al., 2008; Timperio et al., 2004) | traffic safety, absence of stranger danger, sport venues, availability of places to be active | Yes | multilevel linear regression | age, sex, maternal and paternal education, SES, number of siblings, family status, and accelerometer wear time |
| D'Haese et al. 2015 [84] | English | longitudinal | Belgium | Flanders | 76.7 | randomly selected | NR | 321 | 11-12 years old | 2009-2012 | 48.9 | NR | device - ActiGraph GT1M or pedometer and questionnaire (self-reported) | active travel: active transportation to and from school, walking/cycling for transport during leisure time. Sports participation: sport during leisure time. Non-type-specific physical activity: average daily steps | active transportation to and from school; Walking for transport during leisure time (minutes/day); cycling for transport during leisure time (minutes/day); sports during leisure time (minutes/day); average daily steps (steps/day) | Yes | NEWS-Y | residential density, land use mix diversity, land use mix access, street network connectivity, availability of walking and cycling infrastructure, maintenance and quality of walking and cycling infrastructures, aesthetics of the neighbourhood, convenience of recreational facilities and crime and traffic safety | Yes | cross-classified multilevel regression models | two proxy measures of individual SES, educational attainment of mother and father |
| D'Haese et al. 2015 [87] | English | cross-sectional | Belgium | Ghent | 61 | NR | Belgian Environmental Physical Activity Study in Children (BEPAS-child) | 606 | 9-12 years old | 2011-2013 | 53.9 | NR | device - ActiGraph GT1M, GT3X, GT3X+; questionnaire (parent reported) | non-type-specific physical activity: MVPA and PA in specific locations | PA in public recreation spaces; PA in nearby streets and on sidewalks; MVPA | Yes | NEWS-Y | residential density, the accessibility and diversity of land use mix, street connectivity, walk- and cycle infrastructure, aesthetics of the neighborhood, and crime- and traffic safety | Yes | bivariate logistic regression | family SES, sex and age of the child |
| DiGuiseppi et al. 1998 [95] | English | cross-sectional | UK | inner London boroughs of Camden and Islington | NR | randomly selected | NR | 2086 | 6 -10 years old | NR | NR | NR | questionnaire (parent reported) | active travel: travel to school | walked or travelled by car | No | NR | parents worried about abduction or molestation; parents worried about child becoming lost; parents worried about traffic danger | No | logistic regression | age, gender, race/ethnicity, type of school, car ownership, year in school, housing, bicycle ownership |
| do Carmo et al. 2020 [96] | English | cross-sectional | Portugal | Porto, Coimbra, Lisbon | NR | stratified random sampling | NR | 4653 | 7-11 years old | 2016-2017 | 49.4 | NR | questionnaire (parent reported) | non-type-specific physical activity: extracurricular PA | hour/day | No | Environmental Module standard questionnaire | unsafe environment; favourable environment | Yes | structural equation modelling | child's sex, age, SES |
| Dollman and Lewis 2007 [97] | English | cross-sectional | Australia | South Australia | 32 | randomly selected | NR | 1,643 | 9-15 years old | 2005 | NR | NR | questionnaire (parent reported) | active travel: transportation mode to/from school; free time PA; neighbourhood active travel to destinations | minutes per day | No | NR | perceived risk in the neighbourhood (traffic and strangers) | No | analysis of covariance (ANCOVA) | age |
| Dollman and Lewis 2009 [98] | English | cross-sectional | Australia | South Australia | 61.7 | representative across the socioeconomic spectrum | NR | 1518 | 10-15 years old | 2005 | 54.5 | NR | questionnaire (self-reported) | non-type-specific physical activity | Physical Activity Questionnaire for Adolescents: the number of times they performed MVPA | Yes | adapted from Sallis and colleagues | enabling (parent-perceived barriers) the parent survey assessed physical environmental factors, focusing on: risks to safety (strangers and traffic); access to facilities and play opportunities (playgrounds and other children in the neighbourhood); and transport availability | Yes | stepwise multiple regression models | age |
| Ducheyne et al. 2012 [99] | English | cross-sectional | Belgium | Flanders | 80 | randomly selected | NR | 840 | 10-12 years old | 2010–2011 | 49.2 | NR | questionnaire (parent reported) | active travel: the mean number of cycle trips per week to/from school | hour/day [Flemish Physical Activity Q] | No | NEWS-Y | residential density; walking and cycling facilities; connectivity; aesthetics; traffic safety; crime safety | Yes | multivariate logistic regression | age, gender, child BMI, household car access, family structure, independent mobility with the bicycle, parental perceived biking skills of the child |
| Duke et al. 2012 [100] | English | cross-sectional | US | nation-wide | 46.7 | nationally representative | National Survey of Children's Health (NSCH) 2007 | 64,076 | 6-17 years old | 2007 | 48 | NR | questionnaire (parent reported) | non-type-specific physical activity | aerobic PA: the number of days exercising at least 20 minutes (dichotomous) | No | National Survey of Children’s  Health (NSCH) | social capital trust, neighborhood condition, neighbourhood resources, neighbourhood safety | No | linear and logistic regression models | gender, race/ethnicity, age, family structure, household poverty status, parent highest education achieved, health status of youth, parent physical activity level in past week, presence of television in youth’s bedroom |
| Duke et al. 2012 [101] | English | cross-sectional | US | nation-wide | 46.7 | nationally representative | National Survey of Children's Health (NSCH) 2007 | 64,076 | 6-17 years old | 2007-2008 | 48 | NR | questionnaire (parent reported) | sports participation | yes or no | No | National Survey of Children’s  Health (NSCH) | social capital trust, neighborhood condition, neighbourhood resources, neighbourhood safety | No | linear and logistic regression | age, youth gender, youth race and ethnicity, household poverty status, parent's highest education achieved and family structure |
| Dunton et al. 2014 [11] | English | cross-sectional | US | San Bernardino County. | NR | randomly selected | Healthy PLACES "Effects of a Smart Growth Community of Prevention of Family Obesity Risk" | 135 | 8-14 years old | 2009-2010 | 50 | 91 | device - ActiGraph GT1M | non-type-specific physical activity: MVPA | excluding 11pm-5pm & 9am-2pm on weekdays during the school season | Yes | NEWS | park proximity | Yes | logistic regression analyses | age, gender, race/ethnicity and annual household income |
| Durand et al. 2012 [9] | English | cross-sectional | US | San Bernardino County, California | NR | randomly selected | NR | 365 | 11.7±1.49 years old | NR | 49 | NR | device - ActiGraph and questionnaire (self-reported) | non-type-specific physical activity: MVPA. active travel: active transport to/from school | actively commuting or nonactive commuting; average daily MVPA | Yes | NEWS | residential density; land use mix–diversity; land use mix–access; street connectivity; walking infrastructure; aesthetics; traffic hazards; crime; hard parking; lack of cul-de-sacs; hilliness; physical barriers; walkways connecting cul-de-sacs; social interaction | Yes | linear regression | age, gender, race/ethnicity, free/reduced lunch status, and community of residence |
| Engelberg et al. 2016 [102] | English | cross-sectional | US | Baltimore, Maryland-Washington, DC and Seattle-King County, Washington metropolitan | NR | selected from census block representing high or low walkability and high or low income | Teen Environment and Neighborhood (TEAN) | 925 | 12-16 years old | 2009-2011 | 50.4 | NR | device - ActiGraph GT1M/GT3X | non-type-specific physical activity: MVPA | average daily MVPA | Yes | NEWS-Y | aesthetics, traffic safety, pedestrian safety, low crime risk, low stranger dangers | Yes | mixed effects regression | adolescent age, gender, White Non-Hispanic race/ethnicity, parent married/living with a partner, parent with college degree, house type |
| Esteban-Cornejo et al. 2016 [103] | English | cross-sectional | US | Baltimore, MD–Washington, DC, and Seattle–King County, WA | NR | randomly selected | Teen Environment and Neighborhood (TEAN) | 928 | 12-16 years old | 2009-2012 | 50.4 | NR | questionnaire (self-reported) | active travel: active transportation; PA in park; PA around neighbourhood |  | No | NEWS-Y | traffic safety, pedestrian safety, crime safety and stranger danger safety | Yes | mixed-effects linear regression | adolescent sex, age, body mass index percentile, and race/ethnicity; parent marital status, parent education, vehicle per adult in the household, time in neighbourhood, and Walkability Index in 500m |
| Fitzhugh et al. 2021 [104] | English | cross-sectional | US | Alabama, Arkansas, Georgia, Kentucky, Mississippi, North Carolina, Tennessee, Boulder, CO; Burlington, VT; Cary, NC; Davis, CA; Gainesville, FL; Portland, OR. | NR | nationally wide | NR | 2952 | 13-14 years old | 2012 | NR | NR | questionnaire (parent reported) | active travel: active transportation to school | walking/biking vs. others; minutes of MVPA | No | Safety Concerns Scale; Convenience Concerns Scale; Neighbourhood Desirability Scale | safety concerns, convenience concerns, neighbourhood desirability for active transportation | No | logistic regression | age, gender, race, education, marital status, city size, parental PA guidelines |
| Foster et al. 2014 [105] | English | cross-sectional | Australia | Perth | 56.5 | randomly sampled from primary schools in low and high walkable school areas | TRavel, Environment and Kids (TREK) project | 1245 | 10-12 years old | 2007 | 52 | 87.8 | questionnaire (self-reported) | active independent mobility | no IM or some IM | No | NR | parental fear of strangers, informal social control | No | logistic regression | child's age, parent gender, maternal education, school socio-economic status, child sick in previous week, parent confidence in child's ability to travel independently, child confidence in their ability to travel independently, school clustering |
| Franzini et al. 2009 [106] | English | cross-sectional | US | Birmingham, Los Angeles, Houston | 75 | 2-stage probability sampling procedure | Healthy Passages, phase 1 | 544 | 11.30 ± 0.51 years old | 2003 | 55 | NR | questionnaire (self-reported and parents) | non-type-specific physical activity: PA. Active travel: active transport | days of VPA; days of MPA; the number of sports teams in which the child participated during the past 12 months; participation in other organized PA or lessons. Transportation modes. | No | project on Human Development in Chicago Neighbourhoods Community Survey questionnaire | neighbourhood social environment [collective efficacy, socialization of children, exchange, ties, safety] & physical environment [traffic, physical disorder, low density, mainly residential land use] | No | multivariate models | the complex survey design, standard errors |
| Fueyo et al. 2016 [107] | English | cross-sectional | Argentina | Cordoba | 87.3 | randomly selected | NR | 1777 | 9-11 years old | 2011 | 47.4 | NR | questionnaire (self-reported) | non-type-specific physical activity: leisure-time PA; frequency of use of parks or unstructured open spaces for PA | frequency and duration of moderate and vigorous leisure-time PA | No | Neighborhood Environment for Physical Activity Surv+ey | availability of places to be active in, activity options, equipment availability; Parents perceived safety for PA: adult supervision, general neighbourhood safety, traffic safety, lighting; Parents perceived social environment for PA: presence of other children, presence of other people. | No | structural equation modelling | children's school grade and mother's educational level |
| Fyhri and Hjorthol 2009 [108] | English | cross-sectional | Norway | national representative | NR | national representative | NR | 1789 | 6-12 years old | 2005 | 48 | NR | questionnaire (self-reported) | active IM: mode of transport to school, sport activitie and friends | walk, bicycle, public transport, private car | No | NR | parents’ experience of traffic safety on way to school; Parents’ experience of safety concerning other issues on way to school (stranger danger) | No | multivariate analysis and structural equation modelling | NR |
| Galaviz et al. 2016 [109] | English | cross-sectional | US | nation-wide | NR | Multistage probability sampling: SES, location types | Early Childhood Longitudinal Study - Kindergarten Cohort 1998-1999 (ECSL-K) | 9827 | 10-12 years old | 1998-1999 | 51.1 | NR | questionnaire (parent reported) | non-type-specific physical activity: MVPA | days per week of vigorous PA (0-7) | No | NR | neighbourhood safety | No | multivariate logistic regression models & multivariate linear regression models | children’s age in years, gender, race, family socioeconomic status (SES) quintile (ECLS-K composite variable created from household income and parents’ education and occupation with categories ranging from quintile 1 [lowest] to quintile 5 [highest]), how the child prefers to spent his/her free time (in sedentary activities or in physical activities or both), and school location (large/medium city, suburb/large town, small town/rural). |
| Gao et al. 2018 [110] | English | cross-sectional | China | Jinjiang, Fujian | NR | NR | NR | 449 | 12-15 years old | 2012 | NR | NR | investigated | active travel | active commuting or motorized traffic mode | Yes | NR | social environment (the social crime rate in your area, traffic accident rate in your area, theft rate of bicycles around campus); non-motorized traffic facilities (pedestrian sidewalk along the way to school, pedestrian crosswalk along the way to school, traffic lights along the way to school, bikeways along the way to school); road traffic conditions (students cross major road on the way to school, such as four-lane road, traffic volume on the way to school, trucks on the way to school, traffic congestion on the way to school) | No | binary logistic regression model | parents work pattern, household income, grade, parents' gender, number of cars in household |
| Gavand et al. 2019 [111] | English | cross-sectional | US | Baltimore, MD–Washington, DC, and Seattle–King County, WA | NR | randomly selected-two-stage stratified cluster: walkability index | Teen Environment and Neighborhood (TEAN) | 928 | 12-16 years old | 2008-2010 | 50.4 | NR | device - ActiGraph GT1M/GT3X and questionnaire (self-reported) | non-type-specific physical activity: MVPA, days of PA, PA near home, PA at recreation facilities/areas | (1) total minutes of MVPA per valid day (average across all valid wearing days) and (2) average non-school minutes of MVPA created by combining weekend wearing with weekday non-school time wearing (3–11 PM). | Yes | NEWS-Y | availability of multiple types of recreation facilities near home | Yes | mixed-model linear regressions | adolescent age; adolescent gender; adolescent race/ethnicity; highest parent education; ratio of number of vehicles to licensed drivers; years parent lived at residence; and number of people in household |
| Guliani et al. 2015 [28] | English | cross-sectional | Canada | Toronto | NR | stratified cluster: 8 inner-urban schools and 8 inner-suburban schools | Built Environment and Active Transport project | 720 | 10-11 years old | 2010-2011 | 52.5 | mostly mothers participated | questionnaire (parent reported) | active travel: active transportation | walk or driven | No | NR | traffic safety: heavy traffic, drivers go too fast, too many busy streets; walking infrastructure: there are not enough sidewalks, major barriers/obstacles to walking in local neighbourhood | No | structural equation modelling approach | gender, mother employed, vehicles per licensed driver, median household income |
| Hino et al. 2021 [112] | English | cross-sectional | Japan | Chiba | NR | randomly selected - online survey | NR | 1545 | 6-12 years old | 2020 | 48.9 | NR | questionnaire (parent reported) | active travel: travel to/from school modes | everyday walkers (0 days), frequent walkers (more than half a month: 1 or 2 days, 3–5 days, 6–10 days), and less frequent walkers (less than half a month: 11 days or more). | No | NEWS-Y and ANEWS | walking facilities (three items), aesthetics (four items), traffic safety (seven items), and crime safety (six items); neighbourhood cohesion, neighbourhood connection | Yes | multinomial logistic regression analysis | distance to school, population density, grade, sex, and number of cars owned |
| Hofer-Fischanger et al. 2023 [10] | English | cross-sectional | Austria | Styria | NR | four schools | NR | 382 | 6-15 years old | 2017 | 53.9 | 86.9 | questionnaire (parent reported) | active travel: active transport | travel modes (walk, cycle, bus and so on); days per week | No | NR | distance to school; shortcuts for walking | Yes | multilevel logistic regression | age (child), gender (child), educational level (parent) and number of cars in the household |
| Hsu and Saphores 2014 [113] | English | cross-sectional | US | California | NR | randomly selected | National Household Travel Survey 2009 | 2,857 | 5-15 years old | 2009 | 47.7 | NR | questionnaire (parent reported) | active travel: school travel mode | active travel; car; transit (including school bus) | No | NR | distance, traffic volume, traffic speed, crime | No | generalized ordered model | parent's gender, parent's age, parent's race, parental education, parental work status, child's gender, child's age, child's school care, school type, distance to school, number of children in the household, household status, household annual income, parental level of active/transit transportation, urban size, population density |
| Huertas-Delgado et al. 2017 [115] | English | cross-sectional | Spain | Granada | NR | convenience sampling | Pedalea y Anda al Colegio (PACO) Spain study | 628 | 9-16 years old | 2014-2015 | 49.12 | 77.4 | questionnaire (parent reported) | active travel: travel modes | active commuters vs. passive commuters | No | Safe Routes to School Program | traffic (traffic volume, speed of traffic); safety (crime, absence of a policeman at crosswalks, absence of adults to walk with); environment (dangerous intersections, no sidewalks or bike lanes); distance to school | Yes | Chi-square test | No |
| Huertas-Delgado et al. 2018 [114] | English | cross-sectional | Ecuador | Riobamba | NR | convenience sampling | Pedalea y Anda al Colegio (PACO) Ecuador study | 381 | 9-12 years old | 2014-2016 | 76.6 | 76.6 | questionnaire (parent reported) | active travel: active commuting to school and mode of commuting | “How does your child usually travel to school/come back from school?” - [active commuters OR passive commuters] | No | Safe Routes to School Program | traffic (traffic volume and speed of traffic); Safety (crime, absence of a policeman at crosswalks, absence of adults to walk with); environment (dangerous intersections and no sidewalks or bike lanes); distance to school | Yes | logistic regression model | parent's gender, father's educational level, mother's educational level, child's gender |
| Huertas-Delgado et al. 2018 [116] | English | cross-sectional | Belgium | Ghent | NR | spread across the city and suburbs | Belgian Environmental Physical Activity Study in Children (BEPAS-child) | 243 | 12-15 years old | 2014-2016 | 54.7 | 76.5 | questionnaire (parent reported) | active independent mobility | average time spent in active travel (walking or cycling) without adult accompaniment per trip in a usual weektransport in order to assess adolescents' IM. | No | NEWS-Y | traffic safety, crime safety | Yes | multilevel regression analyses | adolescents' gender and parents' highest educational level |
| Hume et al. 2009 [33] | English | longitudinal | Australia | Melbourne | 44 | stratified by high and low SES | Children Living in Active Neighbourhoods (CLAN) | 309 | 9.1 ± 0.33 years old; 14.5 ± 0.62 years old | 2004-2006 | 53 | NR | questionnaire (self-reported and parent reported) | active travel: active commuting to/from school | mean trips/week; percentage of active commuting to/from school; | Yes | NEWS | social factors: people in this area are willing to help neighbours, this is a close-knit neighbourhood; people in this area generally get along; people in this area share the same values; I have many friends in this area; I know many people in this area; This area/community is a good place for children to grow up; This area/community is a good place to live; Physical environmental factors: Public transport is limited in my area; The streets in my local neighbourhood are hilly; There are many cul-de-sacs, courts, or not-through roads near where I live; There are many alternative routes for getting from place to place in my neighbourhood (i.e. there is no need to go the same way every time), neighbourhood is generally free from litter, rubbish, or graffiti; There are no traffic lights/crossings for my child to use; There is heavy traffic in our local streets; Stranger danger is a concern of mine; Road safety is a concern in our area, I am satisfied with the number of pedestrian crossings in my neighbourhood, There are footpaths on most streets in our local neighbourhood, There are lots of parked cars on my street | Yes | bivariable logistic regression | gender, socioeconomic position, and clustering by child’s school (the unit of recruitment) |
| Hunter et al. 2020 [117] | English | cross-sectional | Australia | Melbourne | 15.4 | families attending preschools, primary schools, and secondary schools located within a 3-km radius of each park and randomly selected residents residing within 5km of each park | Recording and Evaluating Activity in a Modified Park (REVAMP) | 729 primary school; 239 secondary school | 5-15 years old | 2013-2015 | 47.7 | NR | questionnaire (parent reported) | non-type-specific physical activity: days of PA (meet PA recommendations) | ≥5 days per week | No | neighbourhood scale items | Physical environment: attractiveness, opportunities to be active, opportunities to exercise, easy to walk, pleasant to walk, park quality, play equipment quality, nearby parks/playgrounds; Social environment: social norms for walking, social norms for exercising, sense of community, social trust and cohesion score, social network score, neighbourhood safety score | Yes | multiple logistic regression | child’s sex and whether their child’s physical activity during the last 7 days was typical for their child (yes/no). Park recruitment area (intervention/control) and year of data collection (baseline/follow-up) |
| Hunter et al. 2022 [118] | English | cross-sectional | Canada | Edmonton | 46.55 | NR | Spatial Health Assessment of Physical Environments (SHAPEs) of Things to Come project | 641 | 6-9 years old | 2009-2011 | 52.3 | NR | device - Steps Count pedometers and questionnaire (parent reported) | non-type-specific physical activity: steps per day; minutes per day for several physical activities | Children’s Leisure Activities Study Survey - | Yes | ANEWS | residential density; land-use mix diversity; land use mix access; street connectivity; infrastructure and safety for walking; aesthetics; traffic hazards; crime; lack of parking; lack of cul-de-sacs; perceived hilliness; physical barriers | Yes | regression analyses | child age, sex, health status, and household income |
| Ikeda et al. 2019 [119] | English | cross-sectional | New Zealand | Auckland | 42.81 | school decile (i.e. a neighbourhood-level measure of socioeconomic status; high, medium, low), child-specific school walkability (high, medium, low) and child-specific neighbourhood destination accessibility (NDAI-C; high, medium, low) | Neighborhoods for Active Kids (NfAK) 2015-2016 | 1085 | 8-13 years old | 2015-2016 | 51 | 69.2 | questionnaire (self-reported) | active travel: school travel mode | active travel OR passive travel | No | NR | neighbourhood safety, neighbourhood cohesion and neighbourhood connection | No | structural equation modelling | child’s school year (grade), sex and ethnicity were reported by schools or their parents/caregivers |
| Jerina et al. 2018 [35] | English | cross-sectional | Slovenia | NR | NR | randomly selected | Children's Active Travel | 669 | 9-11 years old | 2011 | 51.2 | NR | questionnaire (parent reported) | non-type-specific physical activity | all types of PA: minutes per week | No | NR | (1) I fear that my child would become a victim of violence or harassment on his/her way to school; (2) I fear that my child would become a victim of violence or harassment in the playground or at recreational sport facilities; (3) I fear that my child would become a victim of violence or harassment near home; (4) There is no adult person’s control in the playground or at recreational sport facilities; (5) There is no adult person’s control near home; (6) There are no other children in the playground or at recreational sport facilities; (7) There are other children near home; (8) There are too many people in the playground or at recreational sport facilities; and (9) There are too many people for physical activity near home. | No | analysis of variance, Pearson’s and Spearman’s correlation coefficients | No |
| Johansson 2006 [120] | English | cross-sectional | Sweden | Lund and Malmo¨ | NR | randomly selected | NR | 248 | 8-11 years old | NR | 50 | 78 | questionnaire (self-reported) | active travel | Walk, Cycle, Public transport, Car | No | NR | traffic environment (There are few busy roads. Cars are not parked closely along the streets. Noise and pollution from traffic are hardly noticed. There are speed bumps or the like to limit the speed. Busy roads have crossings with traffic lights. There are foot-bridges or underpasses by busy roads.); footpaths and cycle paths (The neighbourhood is well provided with footpaths and cycle paths. The pavements are wide and well kept. The street-lighting is sufficient. Pavements are properly separated from cycle lanes. There are cycle lanes, which are properly separated from the traffic. Walkways and cycle paths are not surrounded by high bushes. Walkways and cycle paths are not perceived as dark and deserted. Underpasses feel light and open.); maintenance (The neighbourhood is neat and well kept. Few of the houses are worn and run down. The neighbourhood is free from litter and graffiti. There are no signs of vandalism. Parks and green open spaces are well maintained. the children’s playground equipment is attractive.) | No | multiple regression analyses | NR |
| Kerr et al. 2008 [121] | English | cross-sectional | US | San Diego County | 91.5 | six clinic sites | NR | 803 | 11-15 years old | NR | 56.2 | NR | questionnaire (self-reported) | non-type-specific physical activity: PA | moderate and/or vigorously physically active (at least 60 minutes/d) one five or more days a week or not | Yes | NEWS | neighbourhood safety | Yes | logistic regression | age, race/ethnicity, and parent education |
| Kim and Heinrich 2016 [122] | English | cross-sectional | US | Manhattan, Austin | 41.3 | stratified-sampled elementary schools | Safe Routes to School (SRTS) program | 832 | 9-10 years old | 2010-2011 | NR | NR | questionnaire (parent reported) | active travel | active or non-active | No | National Safe Routes to School | perceived distance to school, safety concern (my child may get bullied, teased, or harassed; attached by stray dogs; hit by a car) | Yes | multiple logistic regression model | child's race, special lunch program, car ownership, education |
| Kim et al. 2010 [123] | English | cross-sectional | US | nation-wide | 65.98 | nationally representative | National Longitudinal Study of Adolescent Health in the United States, wave 1 (1994-1995) | 13668 | 7-12 years old | 1994-1995 | 49 | NR | questionnaire (self-reported) | non-type-specific physical activity: MVPA | five questions related to MVPA (bouts of MVPA) | No | previous studies | informal control and public nuisance | No | multilevel logistic regression models | adolescents’ age, sex, race/ethnicity, in school status, region and nativity, and the highest parental education and household income, census tract-level neighbourhood factors |
| Kingsly et al. 2020 [124] | English | cross-sectional | India | Chennai | NR | convenience sampling | Madras Diabetes Research Foundation (MDRF) | 324 | 12-17 years old | 2015 | 49 | 73 | questionnaire (self-reported) | active travel: active school travel | active travel OR passive travel | No | NEWS and NEWS-Y | perceived crime, Land use mix–diversity, public recreation spaces, land use-mix access, India-specific barriers/obstruction to walking, Infrastructure for walking/cycling, street connectivity, aesthetics, residential density | Yes | logistic regression models. | age, gender, income groups and mode of recruitment |
| Kneeshaw-Price et al. 2015 [125] | English | cross-sectional | US | San Diego | NR | stratified census block groups; spatial sampling | Neighbourhood Impact on Kids (NIK) | 145 | 6-11 years old | 2007-2009 | 51 | NR | device - ActiGraph GT1M | non-type-specific physical activity: MVPA | average MVPA during the time | Yes | NEWS | stranger danger; general crime and disorder; prior victimization; neighbourhood incivilities | Yes | two multilevel linear random effects models | child’s age, sex, race/ethnicity and household income, collective efficacy |
| Kurka et al. 2015 [126] | English | cross-sectional | US | King County and San Diego | NR | probability sampling | Neighbourhood Impact on Kids (NIK) | 678 | 6-12 years old | 2007 | 49.9 | NR | device - ActiGraph GT1M | non-type-specific physical activity: MVPA | total MVPA; out-of-school MVPA | Yes | NEWS and NEWS-Y | Low Walkable, Unsafe, Parks and Recreation Sparse (LW–U–RS); Moderate Walkable, Transit Access, and Recreation (MW–TR); High Walkable, Transit and Recreation Dense (HW–TRD); Low Walkable and Transit Access, Safe with Average Recreation Facilities (LWT–S) | Yes | multilevel ANCOVA model | child gender, race/ethnicity and age, parent marital status, income, and education, number in household, years at current address, number of cars per legal driver in household, high/low walkability/nutrition, and accelerometer wear time. |
| Larouche et al. 2019 [127] | English | cross-sectional | Canada | Ottawa, Ontario; Trois-Rivières, Québec; and Vancouver, British Columbia | 54.2 | Census data: wide variability in characteristics such as population size and density, income, and language spoken. A stratified sample of schools located in urban, suburban, and rural areas that different in area-level SES was recruited. | Active Transportation (AT) and Independent Mobility (IM) study | 1699 | 8-12 years old | 2016-2017 | 55 | NR | device - pedometer | non-type-specific physical activity: MVPA | steps per day | Yes | NR | traffic safety and social cohesion | No | gender-stratified linear mixed model | age, site, area-level SES, type of urbanization |
| Larsen et al. 2012 [128] | English | cross-sectional | Canada | Toronto | NR | selective sampling took place to ensure representation by income and built environment | Built Environment and Active Transport project | 905 | 10.5 years old (mean) | 2010-2011 | 54 | NR | questionnaire (parent reported) | active travel: travel to/from school modes | walk or vehicle passengers | No | NR | fear of strangers; busy streets to cross; not enough sidewalks; not enough crosswalks | No | binomial logistic regression modelling | distance and the genders of the child and the parent, along with automobile availability |
| Larsen et al. 2018 [129] | English | cross-sectional | Canada | Toronto | NR | selective sampling took place to ensure representation by income and built environment | Built Environment and Active Transport project | 236 | 10.65 ± 0.64 years old | NR | 57.2 | NR | questionnaire (parent reported) | active travel: travel to/from school modes | walk or vehicle passengers | No | NR | not enough sidewalks; not enough crosswalks; fear of strangers | No | binomial logistic regression modelling | child's gender, child's age, vehicles per licenced drivers, educational attainment |
| Li et al. 2012 [130] | Chinese | cross-sectional | China | Hangzhou | 95.2 | stratified suburb and grades | NR | 3867 | 12-16 years old | 2009 | 46.75 | NR | questionnaire (parent reported) | non-type-specific physical activity | met MVPA recommendation: yes or no | No | NR | Jogging or walking near home is not appropriate; Easy access to neighbourhood sports venues; Safe to walk or jog near home during the day; Unsafe to be outdoors near home | No | logistic regression modelling | sex, age and mother's education level |
| Lin et al. 2022 [131] | English | cross-sectional | New Zealand | Auckland | NR | stratified SES, walkability, child-specific destination accessibility and geographic spread across the city | Neighborhoods for Active Kids (NfAK) 2015-2016 | 1102 | 8-13 years old | 2015–2016 | 52.2 | NR | device - ActiGraph GT3X | non-type-specific physical activity: MVPA in near home green place; near home street place; public open near home; public in-door place near home | minutes per day; MVPA out-of-school hours on weekdays between 3pm and 9pm | Yes | adapted and modified from Sampson, Rautenbach, and Earls’s (1997) social cohesion | cohesion, social connectedness | Yes | linear regression model | child ethnicity, age, sex, dwelling type |
| Loucaides et al. 2004 [132] | English | cross-sectional | Cyprus | Lefkosia and Lemesos | 83 | NR | NR | 256 | NR | NR | NR | NR | questionnaire (self-reported) | physical activity: MVPA | Previous Day Physical Activity Questionnaire (PDPAR): | No | NR | Safety in the neighbourhood | No | bivariate correlations | None |
| Machado-Rodrigu et al. 2014 [133] | English | cross-sectional | Portugal | mainland | NR | sex and age-specific proportionate stratified random design with district as the primary sampling unit. | Portuguese Prevalence Study of Obesity in Childhood (PPSOC) | 1886 | 7-9 years old | 2009-2010 | 100 | NR | questionnaire (parent reported) | non-type-specific physical activity: PA outside school. Active travel: mode and duration of travel to/from school | PA at school, time spent walking or cycling to and from school, and time spent playing sports outside of school | No | Environmental Module of the International Physical Activity Prevalence Study | access to destination (two questions); connectivity of the street network; infrastructure for walking and cycling; neighbourhood safety (two questions); social environment (one question); aesthetics (one question); recreation facilities (one question) | Yes | multiple linear regressions | age, BMI, time spent in organized sports, and father and mother education |
| Mammen et al. 2012 [134] | English | cross-sectional | Canada | Greater Toronto and Hamilton Area | 40.3 | randomly selected - Census Division | NR | 490 | 6-14 years old | 2006 | 50.41 | 68.4 | questionnaire (parent reported) | active travel: active school travel | unescorted walkers; escorted walkers; escorted drivers | No | NR | 1. People drive safely enough in my neighbourhood 2. I worry about strangers/bullies approaching my child | No | multinomial logistic regression analysis | child's age, gender, parents’ income, employment status, number of cars/household, number of driver licenses, language spoken, region |
| McCormack et al.2011 [135] | English | cross-sectional | Australia | Perth | 56.6 | stratified by socioeconomic status | TRavel, Environment and Kids (TREK) project | 927 | 10-12 years old | 2007 | 54.3 | NR | device - pedometer | non-type-specific physical activity: PA - steps | pedometer-based cut-points: sex-specific pedometer-based cut-points of ≥15000 steps/day for boys and ≥12000 steps/day for girls | Yes | NR | traffic in neighbourhood; friendliness of neighbourhood, distance to nearest friends house from home, distance to nearest relatives house from home, count of different destinations within a 10 minute walk of home | No | generalized estimating equations | school |
| McCormack et al.2023 [136] | English | cross-sectional | US | King County-Seattle, WA and Baltimore, Maryland regions | NR | neighborhoods selected to represent a wide range of built environments and socioeconomic conditions. | Teen Environment and Neighborhood (TEAN) | 928 | 12-17 years old | 2008-2010 | 50.4 | 79.1 | questionnaire (self-reported) | non-type-specific physical activity: PA | How often are you physically active in/at the following locations? | No | NEWS-Y | proximity to parks and recreation facilities | Yes | linear mixed regression | adolescents’ self-reported age, sex (male or female), race/ethnicity. Household covariates included highest household education (recoded as some college or less, or college degree or more), and caregiver marital/partner status. |
| McDonald et al. 2010 [137] | English | cross-sectional | US | Oakland, Berkeley, Albany, and Richmond, California | 26 | stratified sampling: income, race/ethnicity, walkability,and region of the country | NR | 432 | 10-14 years old | 2006-2007 | 51 | NR | questionnaire (parent reported) | active travel: school travel mode | active travelers vs. nonactive travelers | No | previous studies (Sampson et al. 1999) | social environment | Yes | multivariate matching technique | child's age, child's race, child's sex, household vehicles per adult, household income, and network distance between home and school |
| McMillan 2007 [14] | English | cross-sectional | US | California | NR | NR | Safe Routes to School (SRTS) program | 1128 | 8-10 years old | 2004-2005 | NR | NR | questionnaire (parent reported) | active travel to school | walk/bike; private vehicle or neighbourhood carpool | No | NR | neighbourhood safety: neighborhood is not safe for child to walk/bike to/from school alone; Traffic safety: if child were to walk/bike, would they have to travel on road w/traffic >30 mph | No | factors model | number of children, average annual household income |
| Mehdizadeh et al. 2017 [139] | English | cross-sectional | Iran | Rasht | 80 | random clustered and stratified sampling | NR | 711 | 7-9 years old | 2014 | 50.5 | NR | questionnaire (parent reported) | active travel: travel modes | walking vs. motorized modes | No | previous studies(Mehdizadeh, Mamdoohi, and Nordfjaern 2017; Transport for London 2011) | worry (parental concern about accidents with each of the mentioned modes); perceived walking time to school; access to school service; access to public transport | Yes | binary logit model | pupil's grade, gender, parental age, parents’ educational attainment, parents' driving license status, parents' job status, car ownership, income. |
| Mehdizadeh et al. 2019 [138] | English | cross-sectional | Iran | Rasht | 80 | random clustered and stratified sampling | NR | 711 | 7-9 years old | 2014 | 50.5 | NR | questionnaire (parent reported) | active travel: travel modes | walking vs. motorized modes | No | previous studies(Mehdizadeh, Mamdoohi, and Nordfjaern 2017; Transport for London 2011) | worry (parental concern about accidents with each of the mentioned modes); perceived walking time to school; access to school service; access to public transport | Yes | structural equation model | pupil's grade, gender, parental age, parents’ educational attainment, parents' driving license status, parents' job status, car ownership, income. |
| Millstein et al. 2011 [140] | English | cross-sectional | US | San Diego, CA, Boston, MA, and Cincinnati, OH | 47 | stratified sampling: income, race/ethnicity, walkability, and region of the country | NR | 104 | 5-11 years old | 2005-2006 | 51.9 | NR | questionnaire (parent reported) | non-type-specific physical activity: PA index: overall physical activity, organized PA outside of school. Active travel: active transport to and from school | “Not counting school PE classes, how many days per week do you/your child play or practice team sports?” and “Not counting school PE classes, how many days per week do you/your child have physical activity classes or lessons not in a team sport (like martial arts, dance, tennis)?” | No | NEWS-Y | the pedestrian and automobile traffic safety, crime safety (reverse scored), neighbourhood aesthetics, walking and cycling facilities, street connectivity, land use mix-access, and residential density subscales | Yes | hierarchal linear regression model | site, race, gender, parent education |
| Molnar et al. 2004 [141] | English | cross-sectional | US | Chicago, Illinois | NR | 343 neighborhood clusters; The study gathered a random community sample | Human Development in Chicago Neighbourhoods (PHDCN) | 1378 | 11-16 years old | 1995-2001 | 51.1 | NR | questionnaire (parent reported) | non-type-specific physical activity: participant in recreational activities | hours/week | No | NR | Neighbourhood unsafe to play | No | subsequent multivariate analyses employed hierarchical linear modelling techniques | gender, age, family socioeconomic status, race/ethnicity, BMI, neighbourhood education level |
| Muthuri et al. 2016 [142] | English | cross-sectional | Kenya | Nairobi | NR | conveniently recruited from nonboarding public (lower socioeconomic status [SES]) and private (higher SES) primary schools | International Study of Childhood Obesity, Lifestyle and Environment (ISCOLE) | 563 | 9-11 years old | 2012 | 53.5 | NR | device - ActiGraph GT3X and questionnaire (self-reported) | active travel: active transportation. Non-type-specific physical activity: being sufficiently active, directly measured met MVPA guidelines |  | Yes | NEWS and Neighbourhood Impact on Kids Survey | neighbourhood cohesion; proximity and access to facilities, street connectivity, infrastructure for getting around, aesthetics, and safety from traffic and crime | Yes | univariable analysis | sex, attendance by type of school, maternal education level, paternal education level |
| Nakabazzi et al. 2021 [143] | English | cross-sectional | Uganda | Kampala | 42.6 | randomly selected | NR | 256 | 10-12 years old | NR | 55.5 | NR | device - ActiGraph GT3X | non-type-specific physical activity: MVPA | average daily minutes of MVPA | Yes | NEWS-Africa | residential density; land use mix-diversity (destinations); land use mix-diversity (recreation); land use mix-access; street connectivity; sidewalks infrastructure; crossing infrastructure; paths infrastructure; walking and cycling infrastructure; aesthetics; crime safety; traffic safety; personal safety; stranger danger | Yes | linear regression models | child’s date of birth, sex, and time spent at the current residence |
| Napier et al. 2011 [144] | English | cross-sectional | US | Salt Lake, Utah | 62.99 | School-wide assemblies | NR | 177 | 10-11 years old | 2007 | NR | NR | questionnaire (self-reported) | active travel: walks to school | has students report the total number of times (0-10) they usually walked to (0-5) and from (0-5) school during the week. | No | NR | unsafe to cross (“Streets are dangerous to cross along the route to school”); unsafe traffic (“The traffic along the route makes the walk unsafe”); difficult to walk (“It would be or is difficult for my child to walk from home to school”), too far to walk (“The distance between the school and home is too far to walk”), and crime (“The dangers of crime along the route makes the walk unsafe”). | No | multivariate generalized estimating equation | parent education, home owner, rooms in home, distance by road to school |
| Nayakarathna et al. 2022 [145] | English | cross-sectional | Canada | Ottawa, Ontario, Trois-Rivières, Québec, and Vancouver, British Columbia | 54.2 | stratified by area-level socioeconomic status (SES) and type of urbanization within three regions of Canada | Active Transportation (AT) and Independent Mobility (IM) study | 478 | 9-12 years old | 2016-2017 | 52.3 | NR | device - pedometer SC-StepRx | non-type-specific physical activity | steps per day | Yes | NR | traffic safety, social cohesion | No | gender-stratified linear mixed models | age, site, area-level SES, and type of urbanization |
| Nevelsteen et al. 2012 [146] | English | cross-sectional | Belgium | Flanders | NR | randomly selected school | NR | 7968 | 6-12 years old | 2002-2005 | 51 | NR | questionnaire (parent reported) | active travel: travel mode choice | cycling or walking | No | NR | safety | No | binary logistic model | child's gender, age, older brother or sister |
| Nguyen et al. 2018 [147] | English | cross-sectional | Canada | Kingston | NR | proportional representation across the seasons, sex, age, and 12 electoral districts | NR | 458 | 10-13 years old | 2015-2016 | 49.8 | NR | device - accelerometer and GPS and Google using Personal Activity and Location Measurement (PALMS) | active outdoor play | minutes per day | Yes | Carver 2008 | traffic safety: traffic volume, traffic speed, traffic calming, pedestrian infrastructure | Yes | general linear models | sex, age, season, race, family income, Walk Score, temperature and precipitation |
| Oliver et al. 2011 [148] | English | cross-sectional | New Zealand | South Auckland | NR | randomly selected | Pacific Islands Families (PIF) study and the Pacific Islands Families: Child and Parental Physical Activity and Body Size (PIF:PAC) study | 135 | 6 years old | 2006-2007 | 60 | 100 | device - Actical accelerometer | non-type-specific physical activity: MVPA | MVPA per day | Yes | Neighbourhood Problems Scale | neighbourhood problems - total score | No | generalized estimation equation models | child sex and maternal activity, number of sunlight hours |
| Oluyomi et al. 2014 [149] | English | cross-sectional | US | Austin | 20 | randomly selected | Texas Childhood Obesity Prevention Policy Evaluation (T-COPPE) | 830 | 9-10 years old | 2008-2010 | 50.4 | NR | questionnaire (parent reported) | active travel: active school commute | walking: On most days how does your 4th grade child arrive at school and leave after school. Nonwalkers vs. walkers | No | the National Center for Safe Routes to School Parent Survey; the School Physical Activity and Nutrition (SPAN) parent survey; the Urban Hispanic Perceptions of Environment and Activity Among Kids (UH-PEAK); NEWS | traffic safety; personal safety | Yes | a series of multivariable regression models | socio-demographic - student's ethnicity, any type of public assistance (family), car ownership (family). |
| Olvera et al. 2012 [150] | English | cross-sectional | US | Houston | 53 | NR | NR | 132 | 8-11 years old | 2008-2009 | 41.66 | 100 | device - ActiGraph GT1M | non-type-specific physical activity: MVPA | MVPA daily average | Yes | NEWS and Children’s Leisure Activities Study survey | neighbourhood safety perceptions: including:(1) too much traffic, (2) cars going too fast, (3) no sidewalks, (4) no signals at crosswalks, (5) no lighting, (6) gangs, (7) strangers, and (8) stray dogs. | Yes | standard multiple regression analyses | child's sex, age, place of birth, marital status, level of education, family income, BMI |
| Ozbil et al. 2021 [151] | English | cross-sectional | Turkey | Istanbul | NR | randomly selected | NR | 1802 | 12–14 years old | 2014-2015 | 49.67 | NR | questionnaire (self-reported) | active travel: walk to and from school (both ways); walk to or from school (either way) | yes or no | No | NEWS | accessibility and streets; street network layout | Yes | nominal logistic regression model | child gender, parental education, and household car ownership |
| Pabayo et al. 2011 [152] | English | longitudinal | US | Little Rock Arkansas; Irvine, California; Lawrence, Kansas; Boston, Massachusetts; Philadelphia, Pennsylvania; Pittsburgh, Pennsylvania; Charlottesville, Virginia; Seattle, Washington; Hickory and Morgantown, North Carolina; Madison, Wisconsin | NR | randomly selected | National Institute of Child Health and Human Development Study of Early Child Care and Youth Development (NICHD SECCYD) | 889 | 10-15 years old | 2001-2006 | 49.9 | 100 | device - CSA accelerometer | non-type-specific physical activity: MVPA | minutes of MVPA on weekday/weekend | Yes | NR | social cohesion | No | A quadratic term (age2) was also included to estimate whether or not increases/decreases accelerated/decelerated across time. A second set of analyses was performed by adding the main exposure variables, area economic deprivation, social fragmentation and neighbourhood social cohesion, in order to determine whether there was an effect of these on weekday and weekend physical activity minutes at age 10 years (intercept) and across time (linear and quadratic age growth terms). | geographic region, child ethnicity, maternal education and income-to-needs ratio |
| Pabayo et al. 2012 [153] | English | longitudinal | Canada | Quebec | 83.1 | three-stage strategy | Quebec Longitudinal Study of Child Development (QLSCD) | 710 | 6-8 years old | 2003-2006 | 51.7 | 98 | questionnaire (parent reported) | active travel: active transport to school | active (walking/bicycling) or inactive (school bus, public transit, is driven, or multiple modes). | Yes | previous studies (Buka et al., 2003; Cradock et al., 2009; Pabayo et al., 2011a; Sampson et al., 1997; Greenberg et al., 1999) | neighbourhood quality; social cohesion; neighbourhood decay | No | growth curve models are a generalization of the linear model | gender, birth rank, mother is non-European immigrant, income is insufficient at kindergarten, perception of child's health |
| Panter et al. 2010 [155] | English | cross-sectional | UK | Norfolk | 57 | selected by urban–rural status | Sport, Physical activity and Eating behaviour: Environmental Determinants in Young people (SPEEDY) | 2012 | 9–10 years old | 2012 | 49.7 | NR | questionnaire (self-reported) | active travel: active transport to school | motorised travel, by bicycle and on foot | No | ANEWS | residential density, street connectivity and traffic safety | Yes | multilevel statistical modelling | age, gender, child BMI, household car access, modelled distance to school (kilometres) and maternal travel mode to work |
| Panter et al. 2013 [154] | English | cross-sectional | UK | Norfolk | 57 | representative in county | Sport, Physical activity and Eating behaviour: Environmental Determinants in Young people (SPEEDY) | 912 | 10.23 ± 0.30 years old | 2008 | 59.1 | NR | questionnaire (self-reported) | active travel: active travel to/from school | passive vs active | No | ANEWS | social cohesion; physical neighbourhood environment; the route between home and school (the presence of pavements, cycle-paths, concern about dangerous traffic and concern that something would happen to their child along the route to school); safe to play in the neighbourhood | Yes | multilevel logistic regression | gender, weight status, parental education level |
| Perez et al. 2017 [156] | English | cross-sectional | US | Baltimore, MD/Washington, DC and the Seattle-King County, Washington metropolitan regions | NR | randomly selected | Teen Environment and Neighborhood (TEAN) | 910 | 12-16 years old | 2009-2011 | 49.89 | NR | device - ActiGraph 7164 and GT1M and questionnaire (self-reported) | non-type-specific physical activity: neighbourhood leisure-time physical activity (LTPA); Non-neighbourhood LTPA; Non-school moderate-to vigorous-physical activity (MVPA) | Mean daily non-school MVPA minutes | Yes | NEWS-Y | safety from traffic; pedestrian safety; safety from crime; neighbourhood aesthetics | Yes | mixed-effects linear models | adolescents’ age and work/volunteer status, walkability/income quadrant, site. Accelerometer-based model also adjusted for valid wear time and device used. |
| Pfledderer et al. 2021 [157] | English | cross-sectional | US | a metropolitan area of Utah | NR | convenience sample | NR | 98 | 8-12 years old | 2019 | 44.89 | NR | questionnaire (parent reported) | active travel: school commute mode and frequency | ‘‘In an average school week, how many days does your child use the following modes of transportation to get to and from school?’’ Options for modes of transportation included walking, bicycling, and going by car or bus. Parents were asked to indicate frequency (0-5 days) of each mode of transportation to school and from school separately. | No | Active Where Survey | too many hills; no sidewalks; route is boring; bad lighting; traffic; dangerous crossings; get too hot/cold; no other children walk/bike; too much stuff to carry; easier to drive; planning; crime; bullies; stray dogs; distance | No | partial correlations | school-to home distance and child age |
| Pojani and Boussauw 2014 [158] | English | cross-sectional | Albania | Tirana | 34 | NR | Safe Routes to School | 472 | 11-13 years old | 2004-2005 | 44.3 | NR | questionnaire (parent reported) | active travel to school | walk, bus, car | No | NR | parents' perception of distance | No | logistic regression models | schools |
| Pont et al. 2013 [37] | English | cross-sectional | Australia | Brisbane | NR | comparative site sampling: two suburban areas | NR | 206 | 9-12 years old | 2010 | 51.94 | NR | questionnaire (parent reported) | active travel: active travel to/from school | AT at least once in the past week | No | NR | There are adequate pedestrian crossings for my child to walk to and/or from school; There are sufficient footpaths for my child to walk to and/or from school; It is too far for my child to walk to and/or from school; There are too many busy roads for my child to cross to walk to and/or from school; There are too many roads to cross for my child to walk to and/or from school. I am concerned my child will be kidnapped, molested or hurt by someone else; Because of the amount of concern I have about my child being hurt by someone; I am concerned my child will be hurt in a traffic accident; Because of the amount of concern I have about my child being hurt in a traffic accident. | No | bivariate logistic regression | maternal education and parents’ reported annual household income |
| Pouliou et al. 2015 [159] | English | cross-sectional | UK | nation-wide | NR | stratified clustered | Millennium Cohort Study (MCS) | 6497 | 7 years old | 2008-2009 | 49.1 | NR | device - ActiGraph GT1M | non-type-specific physical activity: MVPA | total physical activity (mean daily counts per minute (cpm) of wearing time, mean daily minutes of moderate to vigorous activity (MVPA) and adherence to current recommended guidelines (at least 60 minutes MVPA per day). | Yes | NR | accessibility to play areas; and whether the area in which they lived (defined as one mile or 20 minutes walk from their house) was perceived to be good and safe to raise children. | No | linear regression models | gender, season, child’s ethnic group, child’s BMI |
| Quigg et al. 2012 [160] | English | cross-sectional^b^ | New Zealand | Dunedin | 85 | specific community | NR | 184 | 5-10 years old | 2007-2008 | 58 | NR | device - ActiGraph GT1M | non-type-specific physical activity: MVPA | total daily MVPA | Yes | NR | neighbourhood score | No | linear mixed model | age- and sex-standardized BMI z-scores; relative income, home ownership, familial support, employment status, educational qualifications, living space, telephone, and transport access; ethnicity |
| Roberts et al. 2016 [36] | English | cross-sectional | US | Washington DC | 10 | stratified sampling strategy: in order to ensure adequate inclusion of diverse built environments | Built Environment and Active Play (BEAP) | 144 | 7-12 years old | 2014 | 50 | NR | questionnaire (parent reported) | active travel | active and non-active: frequency and duration | No | NR | aesthetics, walkability and safety, distance to various destinations | No | univariate logistic regression and multivariable logistic regression analysis | child age, sex and race |
| Roberts et al. 2018 [161] | English | cross-sectional | US | Washington DC | 10 | stratified sampling strategy: in order to ensure adequate inclusion of diverse built environments | Built Environment and Active Play (BEAP) | 144 | 7-12 years old | 2014 | 50 | NR | questionnaire (parent reported) | active travel: active transport to school | ATS-walking, ATS-biking, and ATS public: days * minutes | No | NR | aesthetics, walkability, safety, distance to various destinations | No | univariate logistic regression and multivariable logistic regression analysis | child age, sex and race |
| Rosenberg et al. 2009 [162] | English | cross-sectional | US | San Diego, Boston, and Cincinnati areas | 62 | random selection from neighborhoods | NR | 116 | 5-11 years old | 2005 | NR | 86.1 | questionnaire (parent reported) | active travel and PA | active travel: once per week or more; MVPA: moderate-to-vigorous physical activity guidelines (60 minutes of activity 5 days per week). | Yes | NEWS-Y | land use mix-diversity; pedestrian and automobile traffic safety; crime safety; aesthetics walking and cycling facilities; street connectivity; land use mix-access; residential density; recreation facilities | Yes | one-way analysis of covariance | household income (more than or equal to versus less than $50,000 per year), gender, and race (white or non-white). |
| Ross et al. 2017 [164] | English | cross-sectional | US | Phoenix, Arizona | 8 | convenience sample | Safe Routes to School (SRTS) program | 217 | 10.3 + 1.649 years old | NR | 61 | 61 | questionnaire (parent reported) | active travel: active transport to school | active transport or non-ATS behavior. Using the criteria of walking or biking at least one day a week | No | NR | Safety environment; Physical environment | Yes | logistic regression model | gender, age, grade level, parent's employment status, parent's education level, distance from school |
| Ross et al. 2019 [163] | English | cross-sectional | US | Phoenix, Arizona | NR | randomly selected | Safe Routes to School (SRTS) program | 248 | 8-13 years old | NR | NR | NR | questionnaire (parent reported) | active travel: active transport to school | active transport or non-ATS behavior. The total number of days spent actively transporting to and from school was calculated for each student with possible scores ranging from 0 to 10. | No | Safe Routes to School Program | PE: no sidewalk, 1+ dangerous crossings, too much traffic, too far, crossing guards; SC: trust people in neighbourhood | Yes | structured equation modeling | age and gender |
| Rossen et al. 2011 [165] | English | cross-sectional | US | Baltimore in Maryland | 86 | randomly selected | Multiple Opportunities to Research Excellence (MORE) | 365 | 8-13 years old | 2007 | 53.97 | NR | questionnaire (self-reported) | active travel: active transport to school | active transportation vs. non-active transportation | No | NR | neighbourhood safety | No | generalized estimating equations | child age, gender, self-reported race, parent or guardian’s highest level of education attainment, parent-reported household yearly income, parent employment, parent marital status, how much the parent/caregiver works per week, and how many children the parent/caregiver has |
| Rothman et al. 2015 [166] | English | cross-sectional | Canada | Toronto | 38 | randomly selected | Child pedestrian-motor vehicle collisions and walking to school in the City of Toronto: The role of the built environment | 733 | 9-11 years old | 2011 | 51.9 | NR | questionnaire (parent reported) | active travel: walk to school | “walks 4–5 times/week (defined as frequent walker), versus “walks 0–3 times/week”. | Yes | NR | traffic danger: high school route traffic danger and high school site traffic danger | NR | two repeated-measures logistic regression models | grade, sex |
| Rutten et al. 2013 [167] | English | cross-sectional | Belgium | nation-wide | NR | randomly selected per province | NR | 787 | 10-12 years old | 2009 | NR | 74.6 | device - pedometer and questionnaire (self-reported) | non-type-specific physical activity: PA | steps per day | Yes | NR | neighbourhood safety, neighbourhood facilities | No | multivariate analysis of variance bootstrapping approach | gender |
| Salahuddin et al. 2016 [168] | English | cross-sectional | US | Texas-wide | NR | randomly selected | Texas Childhood Obesity Prevention Policy Evaluation (T-COPPE) | 857 | 9-10 years old | 2009 | 50.29 | NR | questionnaire (parent reported) | active travel: active commuting to/from school | active commuting vs. nonactive commuting | No | NEWS, Urban Hispanic Perceptions of Environment and Activity Among Kids (UH-PEAK), the National Center for Safe Routes To School Parent Survey, AND the School Physical Activity and Nutrition (SPAN) parent survey | social cohesion and perceived safety | Yes | bivariate analysis | race/ethnicity, level of education of parents, whether families received any government assistance, car ownership, and school level SES, language spoken and thought in at home, distance of school from home, neighbourhood walkability index, and school walkability index |
| Sallis et al. 1999 [169] | English | longitudinal | US | NR | 52.9 | NR | NR | 732 | 9.54+0.55 years old | NR | 50.5 | NR | device - Caltrac accelerometer | Caltrac accelerometer | physical activity | Yes | NR | how safe it is for "your child to play outdoors with other children in your neighbourhood without adults supervision" | No | multiple regressions | age, school |
| Sallis et al. 2002 [170] | English | cross-sectional | US | Amherst, Leverett, Shutesbury, and Pelham | NR | randomly selected | NR | 781 | 6-17 years old | 1996-1997 | 51.9 | NR | device - CSA accelerometer and questionnaire (parent reported) | non-type-specific physical activity: parent reported VPA | daily minutes in reported vigorous PA | Yes | NR | access to facilities; park distance; park safety | No | bivariate analysis; Spearman correlation (200 students measured VPA) | child's age, race, dual parent, enjoy PA, coordination, recreation, diet, adult PA, peer support, family support |
| Salmon et al. 2013 [171] | English | cross-sectional | Australia | Victoria | 53 | randomly selected | Resilience for Eating and Activity Despite Inequality (READI) and the Active Independent Mobility (AIM) studies | 613 | 5-12 years old | 2007–2008 | 53 | 100 | device - ActiGraph GT1M | non-type-specific physical activity: MVPA | average minutes per day | Yes | NR | neighbourhood has good places to play; neighbourhood personal safety; neighbourhood road safety concerns; social network | No | linear regression model | maternal education, children’s age and sex |
| Scheiner et al. 2019 [172] | English | cross-sectional | German | Lünen | 60.3 | randomly selected | NR | 1064 | 6-10 years old | 2017 | 49.4 | NR | questionnaire (parent reported) | active travel: mode choice for trips | walking, bicycle, scooter, school bus, other bus, and car passenger, plus combinations of any two | No | previous studies (Guliani et al. 2015) | diffuse fear; pavements and lighting in good condition | Yes | multinomial regression analysis | child age and gender, mother's age (that was consistently significant), presence of a sibling of primary-school age, and parental car use for the commute (that may to some extent capture employment effects). |
| Sener et al. 2019 [173] | English | cross-sectional | US | Texas-wide | NR | randomly selected | National Household Travel Survey with the add-on sample for Texas 2009 | 2783 | 5-15 years old | 2009 | NR | NR | questionnaire (self-reported) | active travel: children’s school travel mode | five categories: private vehicle to and from school, walk/bike to and from school, school bus to and from school, combination private vehicle and walk/bike, and combination private vehicle and school bus. | No | NR | aggressive driving; safety concerns | No | multinomial logit (MNL) model | child's gender, age, African American, attend after school care, attend private school, income level, number of household vehicles, rural area, homeowner, resides in an apartment/condominium; distance to school, percent density, percent population under 18 years old, percent population, total retail employment, single parent, parental education level |
| Siiba 2021 [174] | English | cross-sectional | Ghana | Tamale | 60.28 | stratified sampling strategy - clusters (administrative units) | NR | 842 | 6-12 years old; 13-16 years old | 2018 | 53 | 28.9 | questionnaire (parent reported) | active travel: travel mode | active vs. passive travel | No | ANEWS | safety, crime, school distance and infrastructure conditions | Yes | multivariate regression models | socio-demographics of the parent: gender, age, education level, employment status |
| Silva et al. 2018 [175] | English | cross-sectional | Brazil | Curitiba | NR | randomly selected: primary sampling units | IPEN (International Physical Activity and the Environment Network) Curitiba | 495 | 12-17 years old | 2013-2014 | 50.7 | 74.9 | questionnaire (self-reported) | active travel: active commuting to school | zero time/week versus ≥1 times/week | Yes | NEWS and NEWS-Y | traffic, crime and sum of items for traffic and crime | Yes | poisson regression | adolescents: gender, age group, perception of time spent to walk to school, leisure time moderate-to-vigorous physical activity, period spent in school; parents: car ownership |
| Singh et al. 2008 [176] | English | cross-sectional | US | nation-wide | 68.8 | national representative | National Survey of Children's Health (NSCH) 2003 | 68288 | 6-17 years old | 2003-2004 | 48.52 | NR | questionnaire (parent reported) | non-type-specific physical activity: PA | physically active vs. non physically active; days of physical inactivity in past month | Yes | NR | social capital index, neighbourhood safety | No | multivariate logistic and least squares regression models | age, gender, race/ethnicity, household composition, metropolitan/non-metropolitan and region of residence, primary language spoken at home, social capital, and perceived neighborhood safety. The socioeconomic variables included household or parental education and household income/poverty status. |
| Singh et al. 2009 [177] | English | cross-sectional | US | nation-wide | 68.8 | national representative | National Survey of Children's Health (NSCH) 2003 | 68288 | 6-17 years old | 2003-2005 | 48.52 | NR | questionnaire (parent reported) | non-type-specific physical activity: VPA | VPA vs. non VPA | Yes | NR | social capital index, neighbourhood safety | No | multivariate logistic regression models | age, gender, race/ethnicity, household composition, place of residence, language use, household poverty status, neighbourhood safety, social capital, television viewing, recreational computer use, sleep behaviour, and parental physical activity. |
| Solana et al. 2018 [31] | English | cross-sectional | Spain | Huesca | 80.96 | randomly selected school | Centre for the Promotion of PA and Health (CAPAS-City) | 463 | 9-12 years old | 2017 | 49.5 | 100% mother; 100% father (both) | questionnaire (self-reported) | active travel: active commuting to school | active commuters (≥6 active trips per week) versus ‘passive commuters’ to school (< 6 active trips per week): Mode and Frequency of Commuting to and From School Questionnaire | Yes | Safe Routes to School Program | perceived distance to school, traffic, pedestrian and cycling infrastructure, personal and traffic safety | Yes | multivariate binary logistic regression model | child's age, sex, type of school, mother's or father's age |
| Sullivan et al. 2017 [178] | English | cross-sectional | 12countries | 12 countries | NR | multi-stage sampling frame: SES and school | International Study of Childhood Obesity, Lifestyle and Environment (ISCOLE) | 6161 | 9-11 years old | 2011-2013 | 54.4 | NR | device - accelerometer ActiGraph GT3X+ | non-type-specific physical activity: MVPA | mean minutes/day of MVPA | Yes | NEWS-Y | collective efficacy: social cohesion and social control, and perceived crime | Yes | multi-level mixed effects models | age, sex, and highest parental educational attainment, waking wear time |
| Tappe et al. 2013 [179] | English | cross-sectional | US | Seattle and San Diego | 14.6 | two-stage stratified cluster sampling | Neighbourhood Impact on Kids (NIK) | 724 | 6-10 years old | 2007-2009 | 49.3 | NR | device - accelerometer ActiGraph GT1M and questionnaire (parent reported) | non-type-specific physical activity: MVPA, park activity, neighbourhood activity, 60 minutes+ activity days (outside of school) | neighbourhood activity: how often is your chid physically active? Park activity: how often is your child physically active in/at the following location? 60 minutes+ activity days (outside of school): how many days is/was your child PA for a total of at least 60minutes + per day? | Yes | NEWS-Y | getting around in your neighbourhood; proximity to location; barriers to walking and biking | Yes | multivariate regression models | child sex, age, race, BMI percentile, ethnicity and household income |
| Timperio et al. 2004 [30] | English | cross-sectional | Australia | Melbourne | 44 | stratified cluster sampling | NR | 1210 | 5-6 years old; 10-12 years old | 2001 | 50.95 | NR | questionnaire (parent reported) | active travel: walking and cycling to school | ‘‘how often does your child walk or ride a bike to the following places?’’ - never/rarely (0); less than once per week (0.5); 1–2 times per week (1.5); 3–4 times per week (3.5); 5–6 times per week (5.5); and daily (7). | No | NR | traffic density, road safety, strangers, sporting facilities and public transport in their local area. | No | multivariate logistic regression | school, SES and all other variables |
| Timperio et al. 2006 [180] | English | cross-sectional | Australia | Melbourne | 44 | stratified cluster sampling: school size | NR | 912 | 5-6 years old; 10-12 years old | 2001 | 51.35 | NR | questionnaire (parent reported) | active travel: walking and cycling to school | never: infrequent/occasional (one to four times per week); frequent (five or more times per week) | No | NR | heavy local traffic; strong concern about strangers; strong concern about road safety; no lights/crossings; need to cross several roads; limited public transport; not many other children in neighbourhood | No | bivariate logistic regression analysis | gender and maternal education |
| Trapp et al. 2011 [181] | English | cross-sectional | Australia | Perth | 57 | randomly selected | TRavel, Environment and Kids (TREK) project | 1197 | 10-13 years old | 2007 | 52.13 | 87.9 | questionnaire (self-reported) | active travel: cycling to/from school | ≥1 cycle trips/week vs. no cycle trips/week | No | NR | very/extremely fearful child may be injured if they cycled to school without adult; Very/extremely fearful of stranger danger; Often sees/hears news items about traffic dangers. Neighbourhood is safe enough for children to cycle to school with friends; There are steep hills; My child would have to cross a busy road; There are no safe crossings for my child to use. | No | multivariate logistic regression | child’s grade and highest level of maternal education |
| Trapp et al. 2012 [182] | English | cross-sectional | Australia | Perth | 57 | randomly selected | TRavel, Environment and Kids (TREK) project | 1,298 | 10-13 years old | 2007 | 52.46 | 87.8 | questionnaire (self-reported) | active travel: walking to/from school | <6 trips/week and ≥6 trips/week | No | NR | Very/extremely fearful child may be injured if they cycled to school without adult; Very/extremely fearful of stranger danger; often sees/hears news items about traffic dangers. Neighbourhood is safe enough for children to cycle to school with friends; There are steep hills; My child would have to cross a busy road; There are no safe crossings for my child to use。 | No | multivariate logistic regression | child’s grade and highest level of maternal education |
| Tung et al. 2016 [183] | English | cross-sectional | Malaysia | Klang (city), Selangor(state) | 41.6 | multi-stage sampling: school | NR | 250 | 9-12 years old | NR | 58 | 64 | questionnaire (self-reported) | non-type-specific physical activity: child physical activity level: low, moderate, high | The overall physical activity scores were later categorized as low (10 to 2.33), moderate (2.34 to 3.66) and high (3.67 to 5.00) physical activity levels. | Yes | NEWS | residential density; land-use mix (diversity); land-use mix (access); street connectivity; facilities for walking/cycling; aesthetics; traffic hazards; crime; access to parking; hilly streets; physical/natural obstacles; not many cul-de-sacs | Yes | multiple linear regression analysis | child's gender, parents' education, household income, defensive behaviour |
| Uys et al. 2016 [184] | English | cross-sectional | South Africa | Cape Town | NR | randomly selected within five SES strata | International Study of Childhood Obesity, Lifestyle and Environment (ISCOLE) | 258 | 9-11 years old | 2012-2013 | 56.2 | NR | device - accelerometer ActiGraph GT3X+ | non-type-specific physical activity: MVPA (before school, after school, weekend) | Average daily minutes of MVPA | Yes | NEWS-Y, Neighbourhood Impact on Kids study | proximity to community facilities; neighbourhood safety; traffic safety; walkability; social cohesion | Yes | multilevel linear regression models | age, gender, SES (as measured by family income), school |
| Van Kann et al. 2016 [185] | English | cross-sectional | Netherlands | Southern Limburg | 54 | randomly selected | NR | 722 | 8-12 years old | 2012 | 52.4 | NR | questionnaire (self-reported) | active travel: active school transportation | “On how many days in the past week did you come to school on foot or by bike?” This question resulted in a continuous variable ranging from 0 to 5 days a week | No | NR | traffic load, stranger danger | No | multivariate linear regression analyses | gender, grade, household situation, educational level of parents, ethnicity of parents, |
| Vanwolleghem et al. 2016 [186] | English | cross-sectional | Belgium | Flanders | 70 | randomly selected from suburban and urban | NR | 126 | 10-12 years old | 2013-2014 | 80 | NR | device - accelerometer ActiGraph GT3X or GT1M and questionnaire (self-reported) and GPS-determined transport (in trips/day) | non-type-specific physical activity: MVPA. Active travel: trip modes | Average daily minutes of MVPA; walk, cycling vs passive transport (trips/day) | Yes | NEWS-Y | residential density, land use mix access, street network connectivity, walking and cycling facilities, aesthetics, traffic safety, crime safety | Yes | generalized linear models | age (continuous), sex, SES, wear time and school |
| Vanwolleghem et al. 2017 [187] | English | longitudinal | Belgium | Flanders | 68.2 | randomly selected from suburban and urban | NR | 313 | 11-12 years old | 2009-2010 | 48.88 | NR | questionnaire (self-reported) | active travel: transport to school; transport to leisure time destinations | The Flemish Physical Activity Questionnaire (FPAQ): (1) switching to active transport, (2) maintaining active transport and (3) switching to or maintaining passive transport | Yes | NEWS-Y | residential density, land use mix diversity, land use mix access, street network connectivity, availability and quality of walking and cycling facilities, aesthetics, perceived safety from traffic and crime, convenience of recreational facilities in the neighbourhood, social norm | Yes | multinomial logistic regression analyses | children’s baseline transport behaviour (primary school), separately for transport to school and transport to leisure time destinations. |
| Veitch et al. 2017 [188] | English | longitudinal | Australia | Melbourne | 45 | randomly selected | Resilience for Eating and Activity Despite Inequality (READI) baseline | 184 in 2010; 179 in 2012 | 5-12 years old | 2010-2012 | 55 | 100 | questionnaire (self-reported) | active travel: active transport to/from school; active independent mobility: travel by walking or cycling to local destinations | active transport | No | NR | personal safety, road safety barriers, neighbourhood satisfaction | No | logistic regression models | age and sex of the child, as well as for location (urban/rural), maternal education and employment, and clustering based on suburb of residence (the unit of recruitment) |
| Veugelers et al. 2008 [189] | English | cross-sectional | Canada | Nova Scotia | 51.1 | randomly selected | Children's Lifestyle and School-performance Study | 5471 | 10-11 years old | 2003 | 48.4 | NR | questionnaire (parent reported) | sports participation | longitudinal Study of Children and Youth: 1) number of times per week their child engages in sports with a coach; 2) number of times per week their child engages in sports without a coach | Yes | NR | access to shops; access to playgrounds and parks; access to recreational facilities; safe places for children to play during the day | Yes | multivariate multilevel ordinal logistic regression | child gender, parental education and household income |
| Villanueva et al. 2013 [190] | English | cross-sectional | Australia | Perth | 56.5 | randomly selected | TRavel, Environment and Kids (TREK) project | 977 | 10-12 years old | 2007 | 517 | NR | questionnaire (parent reported) & (self-reported) | active independent mobility | some IM vs no IM | No | NR | parent perception of positive neighbourhood friendliness (4 items), parent perception of safe neighbourhood crossings (2 items), parents’ lack of fear in their child’s personal safety in their neighbourhood | No | multivariate logistic regression | highest level of maternal education, the child’s school year, and whether or not the child was sick in the week prior to survey data collection. |
| Villanueva et al. 2014 [191] | English | cross-sectional | Australia | Perth | 56.5 | randomly selected | TRavel, Environment and Kids (TREK) project | 1061 | 10-12 years old | 2007 | 52.31 | NR | questionnaire (parent reported) & (self-reported) | active independent mobility | some IM vs no IM | No | NR | parent perception of positive neighbourhood friendliness (4 items), parent perception of safe neighbourhood crossings (2 items), parents’ lack of fear in their child’s personal safety in their neighbourhood | No | multilevel logistic regression | highest level of maternal education, the child’s school year and whether or not the child was sick in the week prior to survey data collection, and robust standard errors |
| Wang et al. 2022 [192] | English | cross-sectional | China | Shenyang | NR | Two schools from each of the 13 administrative districts of Shenyang were selected randomly | NR | 3670 | 8-15 years old | 2017 | 49.01 | NR | questionnaire (self-reported) | active travel: active travel to school | active or non-active | No | NEWS-Y | traffic safety and crime safety | Yes | logistic regression model | distance from household to school |
| Waygood and Susilo 2015 [193] | English | cross-sectional | UK | Scotland | NR | state-wide representative | Scottish Household Survey (SHS) 2005/2006 | 315 | 10-11 years old | 2005-2006 | 47.5 | NR | questionnaire (self-reported) | active travel: children’s school travel mode | walk to school vs. otherwise | No | NR | local shops and facilities; friendly people in neighbourhood; traffic is slow or safe | No | two binary (or direct) logistic regression models | family structure, income, education, car availability and use, married or living together, employment status; child's sex and age |
| Wex et al. 2023 [194] | English | cross-sectional | Germany | Leipzig | NR | randomly selected | LIFE Child study | 1070 | 6-10 years old | 2018-2020 | NR | NR | questionnaire (self-reported) | active travel: active school transportation | yes or no | No | Neighborhood Quality of Life Study and NEWS | physical environment: (1) It is too far, (2) It takes too much time, (3) The route is too hilly/exhausting, (4) There are no sidewalks or bike paths, (5) The route is too monotonous/boring, (6) The route is badly illuminated; Safety: (1) The route is too dangerous, (2) It is too dangerous because of crime (strangers, gangs, drugs) (3) There is too much traffic on the route; Social environment: (1) There are no other children walking or going by bike, (2) I am / my child is bullied, annoyed or harassed Individual/Family preferences, (3) I do not have confidence in myself/ My child does not have the confidence to go independently, (4) I have too heavy a load to carry./My child has too heavy a load to carry, (5) An adult gives me a lift on the way to other errands./ It is easier for me to take my child by car when I am on the way to other errands, (6) I have / my child has no desire to do so | Yes | multivariate modelling | child's age, child's gender, SES, number of perceived effective barriers |
| Wilson et al. 2011 [195] | English | cross-sectional^b^ | US | South Carolina | 29.5 | randomly selected | Active by Choice Today (ACT) | 198 | 11.43 ± 0.67 years old | 2005-2007 | 52.5 | 81.8 | device - MTI Actigraph | non-type-specific physical activity: MVPA | average daily MVPA | Yes | NEWS | traffic; crime; social norms; stray dogs | Yes | multilevel modelling | baseline levels of MVPA, free and reduced-price lunch (an index of low-income status), gender, ethnicity, baseline BMI, parental education, and intervention condition. |
| Wilson et al. 2018 [196] | English | cross-sectional | Canada | Southwestern Ontario | 41.7 | randomly selected and schools were selected from stratified groups based on neighbourhood income | Active and Safe Routes to School (ASRTS) and Spatial Temporal Envrionment and Activity Monitoring (STEAM) | 1296 | 9-14 years old | 2010-2013 | 55.2 | NR | questionnaire (parent reported) & (self-reported) | active travel: active travel to/from school | AST to school more than 50% of the total trips vs. AST from school for more than 50% of the total trips | No | Neighborhood Quality of Life Study and the NEWS | Physical Environment: (1) It is too far or takes too much time, (2) There are not enough sidewalks, (3) There are not enough bike paths/ lanes, (4) There are not a lot of trees along the streets in my neighbourhood; Safety: (1) It feels unsafe due to traffic on the route, (2) Most drivers go too fast while driving in our neighbourhood, (3) There are too many busy streets to cross, (4) There is a lot of crime in our neighbourhood, (5) It feels unsafe to walk by myself around my neighbourhood during the day, (6) It feels unsafe to walk with friends or siblings in my neighbourhood during the day | Yes | logistic regression models | child's gender, age, parental education level, parent occupation status, siblings, number of vehicles in family, distance home to school, urbanicity |
| Woldeamanuel 2016 [197] | English | cross-sectional | US | nation-wide | NR | nationally representative | National Household Travel Survey with the add-on sample for Texas 2009 | 7550 | 12-16 years old | 2009 | 47 | NR | questionnaire (parent reported) | active travel | modes: personal vehicle, public transit, and walk/bike | No | NR | crime; school distance; traffic speed; traffic amount | No | binomial logit model | student's gender, school type, urban size, household income, household size, number of vehicles in the HH, number of eligible drivers in the HH |
| Yu and Woo 2017 [77] | English | cross-sectional | Republic of Ireland | nation-wide | NR | national study | Growing Up in Ireland (GUI) second wave 2012 | 7525 | 13 years old | 2011-2012 | NR | NR | questionnaire (self-reported) | non-type-specific physical activity: MVPA | days of exercise that made them breathe hard and heartbeat fast | Yes | NR | safety of walking and playing; neighbourhood safety | No | structural equation models (SEM) to test the hypothesized conceptual framework (the mediating effect of parental safety concern on adolescent’s physical activity) | adolescent's gender, health, parent's highest education, parent's PA, walk/bike to school |
| Žaltauskė and Petrauskienė 2016 [198] | English | cross-sectional | Lithuania | 10 districts | NR | NR | World Health Organization (WHO) European Childhood Obesity Surveillance Initiative (COSI) | 3802 | 7-8 years old | 2013 | 50.2 | NR | questionnaire (parent reported) | non-type-specific physical activity: frequency of daily PA; attendance of sport or dancing clubs | (1) Time spent by children in physically active way from moderate-to-vigorous intensity: less than 1h a day; 1-2 h a day; more than 2 hours a day. (2) Attendance to sport or dancing clubs | No | NR | roads safety to/from school, recreation areas | No | binary logistic regression | gender |
| Ziviani et al. 2004 [38] | English | cross-sectional | Australia | Brisbane | NR | NR | National Center for Chronic Disease Prevention and Health Promotion (2002) | 164 | 9.1 ± 2.02 years old | NR | 53.66 | NR | questionnaire (parent reported) | active travel: active travel to/from school | walking to school at least once per week, and never walking to school | No | NR | distance; traffic; available outside shelter; manned crossings; child’s personal safety; lack of adult company; lack of child company | No | logistic regression | child’s name, age, grade, address, number of siblings, approximate distance lived from the school and parents’ hours of work |

Abbreviations: BMI, body mass index; IM, independent mobility; MVPA: moderate-to-vigorous physical activity; NR: not report; NEWS: Neighbourhood Environment Walkability Scale; NEWS-Y: Neighbourhood Environment Walkability Scale for Youth; PA: physical activity; SES, social ecological status; VPA: vigorous physical activity.

^a^ Results from longitudinal and cross-sectional data were presented

^b^ Cross-sectional data were used to analyse the associations between parental perceptions of neighbourhood environment and children’s physical activity
