# Supplementary material for: Associations between parental perceptions of neighbourhood environment and physical activity in children and adolescents: a systematic review including 149 studies
Source: Int J Behav Nutr Phys Act. 2025 Jun 6;22:70. doi: 10.1186/s12966-025-01733-8 (PMC12143044; doi:10.1186/s12966-025-01733-8)
Supplement: Supplementary file 5 — Additional file 5. [file 12966_2025_1733_MOESM5_ESM.docx]

**Additional file 5. Methodological quality of included papers: detailed results**

| **Author, year** | **Study design** | **Sample size** | **Stratification** | **Response rate** | **Parental perceptions of neighbourhood environment measures shown to be valid and reliable** | **Physical activity outcome measures shown to be valid and reliable** | **Adjustment for socio-demographic covariates (age, sex and education)** | **Adjustment for self-selection into neighbourhoods** | **Analytical approach accounted for area-level clustering** | **Analytical approach accounted for distributional assumptions** | **Analyses conducted and presented correctly** | **Overall score** | **Level** |
| --- | --- | --- | --- | --- | --- | --- | --- | --- | --- | --- | --- | --- | --- |
| Adkins et al. 2004 [52] | 0 | 0 | 0 | 0 | 1 | 1 | 0 | 0 | 0 | 0 | 0.33 | 2.33 | low |
| Aliyas 2022 [41] | 0 | 1 | 0 | 0 | 1 | 1 | 1 | 0 | 0 | 0 | 0.33 | 4.33 | low |
| Aliyas et al. 2022 [53] | 0 | 1 | 1 | 0 | 1 | 0 | 0 | 0 | 0 | 0.33 | 0.33 | 3.66 | low |
| Allen and Vella 2015 [27] | 1 | 1 | 0 | 1 | 0 | 0 | 0 | 0 | 0 | 0.33 | 0.33 | 3.66 | low |
| Appelhans and Li 2016 [54] | 0 | 0 | 0 | 0 | 1 | 1 | 1 | 0 | 0 | 0 | 0.33 | 3.33 | low |
| Aranda-Balboa et al. 2021 [55] | 0 | 1 | 0 | 1 | 1 | 1 | 0 | 0 | 0 | 0.33 | 0.33 | 4.66 | low |
| Babey et al. 2008 [57] | 0 | 1 | 0 | 0 | 0 | 0 | 1 | 0 | 0 | 0.33 | 0.33 | 2.66 | low |
| Babey et al. 2009 [56] | 0 | 1 | 0 | 0 | 0 | 1 | 0 | 0 | 0 | 0.33 | 0.33 | 2.66 | low |
| Barnett et al. 2019 [59] | 0 | 1 | 1 | 1 | 1 | 1 | 1 | 1 | 0.33 | 0.33 | 0.33 | 7.99 | moderate |
| Barnett et al. 2019 [58] | 0 | 1 | 1 | 1 | 1 | 1 | 1 | 1 | 0.33 | 0.33 | 0.33 | 7.99 | moderate |
| Baskin et al. 2013 [60] | 0 | 0.5 | 0 | 0 | 0 | 1 | 0 | 0 | 0 | 0.33 | 0.33 | 2.16 | low |
| Beets and Foley 2007 [61] | 0 | 1 | 1 | 1 | 0 | 0 | 1 | 0 | 0.33 | 0.33 | 0.33 | 4.99 | low |
| Bell et al. 2020 [62] | 0 | 0.5 | 1 | 1 | 0 | 0 | 1 | 0 | 0 | 0 | 0.33 | 3.83 | low |
| Brewer and Kimbro 2014 [63] | 0 | 1 | 0 | 1 | 0 | 0 | 1 | 0 | 0.33 | 0.33 | 0.33 | 3.99 | low |
| Bringolf-Isler et al. 2008 [64] | 0 | 1 | 0 | 1 | 0 | 0 | 0 | 0 | 0 | 0.33 | 0.33 | 2.66 | moderate |
| Bringolf-Isler et al. 2010 [65] | 0 | 1 | 0 | 1 | 0 | 1 | 0 | 0 | 0.33 | 0.33 | 0.33 | 3.99 | low |
| Bringolf-Isler et al. 2019 [66] | 0 | 1 | 0 | 1 | 1 | 1 | 1 | 0 | 0 | 0.33 | 0.33 | 5.66 | moderate |
| Buck et al. 2015 [67] | 0 | 1 | 0 | 0 | 0 | 1 | 0 | 0 | 0.33 | 0.33 | 0.33 | 2.99 | low |
| Buliung et al. 2017 [68] | 0 | 1 | 1 | 0 | 0 | 0 | 1 | 0 | 0 | 0.33 | 0.33 | 3.66 | low |
| Butte et al. 2014 [69] | 1 | 0.5 | 0 | 0 | 1 | 1 | 1 | 0 | 0.33 | 0 | 0.33 | 5.16 | low |
| Cadogan et al. 2014 [70] | 0 | 1 | 0 | 0 | 0 | 1 | 1 | 0 | 0 | 0.33 | 0.33 | 3.66 | low |
| Carlson et al. 2014 [71] | 0 | 0.5 | 1 | 0 | 1 | 1 | 0 | 0 | 0.33 | 0 | 0.33 | 4.16 | low |
| Carson et al. 2010 [72] | 0 | 1 | 1 | 0 | 1 | 1 | 1 | 0 | 0.33 | 0.33 | 0.33 | 5.99 | moderate |
| Carver et al. 2005 [74] | 0 | 1 | 0 | 1 | 1 | 1 | 1 | 0 | 0 | 0.33 | 0.33 | 5.66 | moderate |
| Carver et al. 2008 [32] | 0 | 1 | 1 | 0 | 1 | 1 | 0 | 0 | 0 | 0.33 | 0.33 | 4.66 | low |
| Carver et al. 2014 [76] | 1 | 1 | 0 | 0 | 1 | 0 | 0 | 0 | 0.33 | 0.33 | 0.33 | 3.99 | low |
| Carver et al. 2015 [75] | 0 | 1 | 0 | 0 | 0 | 0 | 1 | 0 | 0.33 | 0.33 | 0.33 | 2.99 | low |
| Carver et al. 2023 [73] | 1 | 1 | 0 | 0 | 1 | 1 | 1 | 1 | 0.33 | 0.33 | 0.33 | 6.99 | moderate |
| Chillón et al. 2014 [78] | 0 | 1 | 0 | 1 | 0 | 1 | 1 | 0 | 0.33 | 0.33 | 0.33 | 4.99 | low |
| Cohen et al. 2017 [79] | 2 | 1 | 1 | 1 | 1 | 1 | 1 | 0 | 0.33 | 0.33 | 0.33 | 8.99 | high |
| Côté-Lussier et al. 2015 [80] | 0 | 1 | 0 | 0 | 0 | 1 | 1 | 0 | 0 | 0.33 | 0.33 | 3.66 | low |
| Crawford et al. 2010 [81] | 1 | 1 | 0 | 0 | 1 | 1 | 1 | 0 | 0.33 | 0.33 | 0.33 | 5.99 | moderate |
| Curriero et al. 2013 [82] | 0 | 1 | 0 | 0 | 0 | 0 | 1 | 0 | 0.33 | 0.33 | 0.33 | 2.99 | low |
| Cutumisu et al. 2014 [83] | 0 | 1 | 0 | 0 | 0 | 0 | 1 | 0 | 0 | 0.33 | 0.33 | 2.66 | low |
| Datar et al. 2013 [88] | 1 | 1 | 0 | 1 | 0 | 0 | 1 | 1 | 0.33 | 0.33 | 0.33 | 5.99 | moderate |
| Datar et al. 2015 [89] | 0 | 1 | 0 | 0 | 1 | 0 | 1 | 1 | 0 | 0.33 | 0.33 | 4.66 | low |
| Davidson et al. 2010 [90] | 0 | 1 | 0 | 0 | 0 | 1 | 1 | 0 | 0.33 | 0.33 | 0.33 | 4.99 | low |
| Davison et al. 2012 [91] | 0 | 1 | 0 | 0 | 1 | 1 | 1 | 0 | 0.33 | 0.33 | 0.33 | 4.99 | low |
| De Meester et al. 2014 [92] | 0 | 1 | 0 | 1 | 1 | 1 | 1 | 0 | 0.33 | 0.33 | 0.33 | 5.99 | moderate |
| DeWeese et al. 2013 [94] | 0 | 1 | 0 | 0 | 0 | 1 | 0 | 0 | 0.33 | 0.33 | 0.33 | 2.99 | low |
| DeWeese et al. 2022 [93] | 1 | 1 | 0 | 0 | 0 | 1 | 1 | 0 | 0.33 | 0.33 | 0.33 | 4.99 | low |
| D'Haese et al. 2011 [85] | 0 | 1 | 0 | 1 | 1 | 1 | 0 | 0 | 0.33 | 0.33 | 0.33 | 4.99 | low |
| D'Haese et al. 2013 [86] | 0 | 1 | 0 | 0 | 1 | 1 | 1 | 0 | 0.33 | 0.33 | 0.33 | 4.99 | low |
| D'Haese et al. 2015 [84] | 1 | 1 | 0 | 2 | 1 | 1 | 1 | 0 | 0.33 | 0.33 | 0.33 | 7.99 | moderate |
| D'Haese et al. 2015 [87] | 0 | 1 | 0 | 1 | 1 | 1 | 1 | 0 | 0.33 | 0.33 | 0.33 | 5.99 | moderate |
| DiGuiseppi et al. 1998 [95] | 0 | 1 | 0 | 0 | 0 | 0 | 1 | 0 | 0.33 | 0.33 | 0.33 | 2.99 | low |
| do Carmo et al. 2020 [96] | 0 | 1 | 0 | 0 | 1 | 0 | 1 | 0 | 0 | 0.33 | 0.33 | 3.66 | low |
| Dollman and Lewis 2007 [97] | 0 | 1 | 0 | 0 | 0 | 0 | 1 | 0 | 0 | 0.33 | 0.33 | 2.66 | low |
| Dollman and Lewis 2009 [98] | 0 | 1 | 1 | 1 | 1 | 1 | 1 | 0 | 0.33 | 0.33 | 0.33 | 6.99 | moderate |
| Ducheyne et al. 2012 [99] | 0 | 1 | 0 | 1 | 1 | 0 | 1 | 0 | 0.33 | 0.33 | 0.33 | 4.99 | low |
| Duke et al. 2012 [100] | 0 | 1 | 0 | 1 | 0 | 0 | 1 | 0 | 0 | 0.33 | 0.33 | 3.66 | low |
| Duke et al. 2012 [101] | 0 | 1 | 0 | 1 | 0 | 0 | 1 | 0 | 0 | 0.33 | 0.33 | 3.66 | low |
| Dunton et al. 2014 [11] | 0 | 0.5 | 0 | 0 | 1 | 1 | 1 | 0 | 0 | 0 | 0.33 | 3.83 | low |
| Durand et al. 2012 [9] | 0 | 1 | 0 | 0 | 1 | 1 | 1 | 0 | 0 | 0.33 | 0.33 | 4.66 | low |
| Engelberg et al. 2016 [102] | 0 | 1 | 1 | 0 | 1 | 1 | 1 | 0 | 0.33 | 0.33 | 0.33 | 5.99 | moderate |
| Esteban-Cornejo et al. 2016 [103] | 0 | 1 | 0 | 0 | 1 | 0 | 1 | 0 | 0.33 | 0.33 | 0.33 | 3.99 | low |
| Fitzhugh et al. 2021 [104] | 0 | 1 | 0 | 1 | 0 | 0 | 0 | 0 | 0 | 0.33 | 0.33 | 2.66 | low |
| Foster et al. 2014 [105] | 0 | 1 | 0 | 0 | 0 | 0 | 1 | 0 | 0.33 | 0.33 | 0.33 | 2.99 | low |
| Franzini et al. 2009 [106] | 0 | 1 | 0 | 1 | 0 | 0 | 0 | 0 | 0.33 | 0.33 | 0.33 | 2.99 | low |
| Fueyo et al. 2016 [107] | 0 | 1 | 0 | 1 | 0 | 0 | 0 | 0 | 0 | 0.33 | 0.33 | 2.66 | low |
| Fyhri and Hjorthol 2009 [108] | 0 | 1 | 0 | 0 | 0 | 0 | 0 | 0 | 0 | 0.33 | 0.33 | 1.66 | low |
| Galaviz et al. 2016 [109] | 0 | 1 | 1 | 0 | 0 | 0 | 0 | 0 | 0 | 0.33 | 0.33 | 2.66 | low |
| Gao et al. 2018 [110] | 0 | 1 | 0 | 0 | 0 | 1 | 1 | 0 | 0 | 0.33 | 0.33 | 3.66 | low |
| Gavand et al. 2019 [111] | 0 | 1 | 1 | 0 | 1 | 1 | 1 | 0 | 0.33 | 0.33 | 0.33 | 5.99 | moderate |
| Guliani et al. 2015 [28] | 0 | 1 | 1 | 0 | 0 | 0 | 0 | 0 | 0 | 0.33 | 0.33 | 2.66 | low |
| Hino et al. 2021 [112] | 0 | 1 | 0 | 0 | 1 | 0 | 1 | 0 | 0 | 0.33 | 0.33 | 3.66 | low |
| Hofer-Fischanger et al. 2023 [10] | 0 | 1 | 0 | 0 | 1 | 0 | 1 | 0 | 0 | 0.33 | 0.33 | 3.66 | low |
| Hsu and Saphores 2014 [113] | 0 | 1 | 0 | 0 | 0 | 0 | 0 | 0 | 0 | 0.33 | 0.33 | 1.66 | low |
| Huertas-Delgado et al. 2017 [115] | 0 | 1 | 0 | 0 | 1 | 0 | 0 | 0 | 0 | 0.33 | 0.33 | 2.66 | low |
| Huertas-Delgado et al. 2018 [114] | 0 | 1 | 0 | 0 | 1 | 0 | 0 | 0 | 0 | 0 | 0.33 | 4.16 | low |
| Huertas-Delgado et al. 2018 [116] | 0 | 0.5 | 1 | 0 | 1 | 0 | 1 | 0 | 0.33 | 0 | 0.33 | 2.33 | low |
| Hume et al. 2009 [33] | 1 | 1 | 1 | 0 | 1 | 1 | 1 | 0 | 0.33 | 0.33 | 0.33 | 6.99 | moderate |
| Hunter et al. 2020 [117] | 0 | 1 | 1 | 0 | 1 | 0 | 1 | 0 | 0.33 | 0.33 | 0.33 | 4.99 | low |
| Hunter et al. 2022 [118] | 0 | 1 | 0 | 0 | 1 | 1 | 1 | 0 | 0 | 0.33 | 0.33 | 4.66 | low |
| Ikeda et al. 2019 [119] | 0 | 1 | 1 | 0 | 0 | 0 | 0 | 0 | 0.33 | 0.33 | 0.33 | 2.99 | low |
| Jerina et al. 2018 [35] | 0 | 1 | 0 | 0 | 0 | 0 | 0 | 0 | 0 | 0 | 0.33 | 1.33 | low |
| Johansson 2006 [120] | 0 | 1 | 0 | 0 | 0 | 0 | 0 | 0 | 0 | 0.33 | 0.33 | 1.66 | low |
| Kerr et al. 2008 [121] | 0 | 1 | 0 | 1 | 1 | 1 | 1 | 0 | 0 | 0.33 | 0.33 | 5.66 | moderate |
| Kim and Heinrich 2016 [122] | 0 | 1 | 0 | 0 | 1 | 0 | 1 | 1 | 0 | 0.33 | 0.33 | 4.66 | low |
| Kim et al. 2010 [123] | 0 | 1 | 0 | 1 | 0 | 0 | 1 | 0 | 0 | 0.33 | 0.33 | 3.66 | low |
| Kingsly et al. 2020 [124] | 0 | 1 | 0 | 0 | 1 | 0 | 1 | 0 | 0.33 | 0.33 | 0.33 | 3.99 | low |
| Kneeshaw-Price et al. 2015 [125] | 0 | 0.5 | 1 | 0 | 1 | 1 | 1 | 0 | 0.33 | 0 | 0.33 | 5.16 | low |
| Kurka et al. 2015 [126] | 0 | 1 | 0 | 0 | 1 | 1 | 1 | 0 | 0.33 | 0.33 | 0.33 | 4.99 | low |
| Larouche et al. 2019 [127] | 0 | 1 | 1 | 0 | 0 | 1 | 1 | 0 | 0.33 | 0.33 | 0.33 | 4.99 | low |
| Larsen et al. 2012 [128] | 0 | 1 | 1 | 1 | 0 | 0 | 0 | 0 | 0 | 0.33 | 0.33 | 3.66 | low |
| Larsen et al. 2018 [129] | 0 | 0.5 | 1 | 1 | 0 | 0 | 0 | 0 | 0.33 | 0 | 0.33 | 3.16 | low |
| Li et al. 2012 [130] | 0 | 1 | 0 | 1 | 0 | 0 | 0 | 0 | 0.33 | 0.33 | 0.33 | 2.66 | low |
| Lin et al. 2022 [131] | 0 | 1 | 1 | 0 | 1 | 1 | 0 | 0 | 0.33 | 0.33 | 0.33 | 4.99 | low |
| Loucaides et al. 2004 [132] | 0 | 1 | 0 | 1 | 0 | 0 | 1 | 0 | 0 | 0.33 | 0.33 | 3.66 | low |
| Machado-Rodrigu et al. 2014 [133] | 0 | 1 | 1 | 0 | 1 | 0 | 1 | 0 | 0.33 | 0.33 | 0.33 | 4.99 | low |
| Mammen et al. 2012 [134] | 0 | 1 | 0 | 0 | 0 | 0 | 0 | 0 | 0 | 0.33 | 0.33 | 1.66 | low |
| McCormack et al.2011 [135] | 0 | 1 | 1 | 0 | 0 | 1 | 0 | 0 | 0.33 | 0.33 | 0.33 | 3.99 | low |
| McCormack et al.2023 [136] | 0 | 1 | 1 | 0 | 1 | 0 | 1 | 0 | 0 | 0.33 | 0.33 | 4.66 | low |
| McDonald et al. 2010 [137] | 0 | 1 | 1 | 0 | 1 | 0 | 1 | 0 | 0 | 0.33 | 0.33 | 4.66 | low |
| McMillan 2007 [14] | 0 | 1 | 1 | 0 | 0 | 0 | 0 | 0 | 0.33 | 0.33 | 0.33 | 2.99 | low |
| Mehdizadeh et al. 2017 [139] | 0 | 1 | 0 | 0 | 1 | 0 | 1 | 0 | 0 | 0.33 | 0.33 | 3.66 | low |
| Mehdizadeh et al. 2019 [138] | 0 | 1 | 0 | 0 | 1 | 0 | 1 | 0 | 0 | 0.33 | 0.33 | 3.66 | low |
| Millstein et al. 2011 [140] | 0 | 0.5 | 1 | 0 | 1 | 0 | 0 | 0 | 0.33 | 0 | 0.33 | 3.16 | low |
| Molnar et al. 2004 [141] | 0 | 1 | 0 | 0 | 0 | 0 | 1 | 0 | 0 | 0.33 | 0.33 | 2.66 | low |
| Muthuri et al. 2016 [142] | 0 | 1 | 1 | 0 | 1 | 1 | 0 | 0 | 0 | 0.33 | 0.33 | 4.66 | low |
| Nakabazzi et al. 2021 [143] | 0 | 0.5 | 0 | 0 | 1 | 1 | 1 | 0 | 0.33 | 0.33 | 0.33 | 4.49 | low |
| Napier et al. 2011 [144] | 0 | 0.5 | 0 | 1 | 0 | 0 | 1 | 0 | 0.33 | 0 | 0.33 | 3.16 | low |
| Nayakarathna et al. 2022 [145] | 0 | 1 | 1 | 0 | 0 | 1 | 1 | 0 | 0.33 | 0.33 | 0.33 | 4.99 | low |
| Nevelsteen et al. 2012 [146] | 0 | 1 | 0 | 0 | 0 | 0 | 1 | 0 | 0 | 0 | 0.33 | 2.33 | low |
| Nguyen et al. 2018 [147] | 0 | 1 | 0 | 0 | 1 | 1 | 1 | 0 | 0 | 0.33 | 0.33 | 4.66 | low |
| Oliver et al. 2011 [148] | 0 | 0.5 | 0 | 0 | 0 | 1 | 0 | 0 | 0 | 0.33 | 0.33 | 2.16 | low |
| Oluyomi et al. 2014 [149] | 0 | 1 | 0 | 0 | 1 | 0 | 0 | 0 | 0 | 0.33 | 0.33 | 2.66 | low |
| Olvera et al. 2012 [150] | 0 | 0.5 | 0 | 0 | 1 | 1 | 0 | 0 | 0 | 0 | 0.33 | 2.83 | low |
| Ozbil et al. 2021 [151] | 0 | 1 | 0 | 0 | 1 | 0 | 1 | 0 | 0.33 | 0.33 | 0.33 | 3.99 | low |
| Pabayo et al. 2011 [152] | 1 | 1 | 0 | 0 | 0 | 1 | 0 | 0 | 0.33 | 0.33 | 0.33 | 3.99 | low |
| Pabayo et al. 2012 [153] | 1 | 1 | 0 | 1 | 0 | 1 | 0 | 0 | 0 | 0.33 | 0.33 | 4.66 | low |
| Panter et al. 2010 [155] | 0 | 1 | 1 | 0 | 1 | 0 | 1 | 0 | 0.33 | 0.33 | 0.33 | 4.99 | low |
| Panter et al. 2013 [154] | 0 | 1 | 0 | 1 | 1 | 0 | 1 | 0 | 0.33 | 0.33 | 0.33 | 4.99 | low |
| Perez et al. 2017 [156] | 0 | 1 | 0 | 0 | 1 | 1 | 1 | 0 | 0.33 | 0.33 | 0.33 | 4.99 | low |
| Pfledderer et al. 2021 [157] | 0 | 0 | 0 | 0 | 0 | 0 | 1 | 0 | 0 | 0 | 0.33 | 1.33 | low |
| Pojani and Boussauw 2014 [158] | 0 | 1 | 0 | 0 | 0 | 0 | 1 | 0 | 0.33 | 0.33 | 0.33 | 2.99 | low |
| Pont et al. 2013 [37] | 0 | 0.5 | 0 | 0 | 0 | 0 | 1 | 0 | 0 | 0 | 0.33 | 1.83 | low |
| Pouliou et al. 2015 [159] | 0 | 1 | 0 | 0 | 0 | 1 | 1 | 0 | 0.33 | 0.33 | 0.33 | 3.99 | low |
| Quigg et al. 2012 [160] | 0 | 0.5 | 0 | 1 | 0 | 1 | 0 | 0 | 0.33 | 0.33 | 0.33 | 3.49 | low |
| Roberts et al. 2016 [36] | 0 | 0.5 | 1 | 0 | 0 | 0 | 1 | 0 | 0 | 0 | 0.33 | 2.83 | low |
| Roberts et al. 2018 [161] | 0 | 0.5 | 1 | 0 | 0 | 0 | 1 | 0 | 0 | 0 | 0.33 | 2.83 | low |
| Rosenberg et al. 2009 [162] | 0 | 1 | 1 | 1 | 1 | 1 | 0 | 0 | 0.33 | 0.33 | 0.33 | 5.99 | moderate |
| Ross et al. 2017 [164] | 0 | 0.5 | 0 | 0 | 1 | 0 | 1 | 0 | 0 | 0 | 0.33 | 2.83 | low |
| Ross et al. 2019 [163] | 0 | 0.5 | 0 | 0 | 1 | 0 | 1 | 0 | 0 | 0.33 | 0.33 | 3.16 | low |
| Rossen et al. 2011 [165] | 0 | 1 | 0 | 1 | 0 | 0 | 1 | 0 | 0.33 | 0.33 | 0.33 | 3.99 | low |
| Rothman et al. 2015 [166] | 0 | 1 | 0 | 0 | 1 | 1 | 1 | 0 | 0.33 | 0.33 | 0.33 | 5.00 | low |
| Rutten et al. 2013 [167] | 0 | 1 | 0 | 0 | 0 | 1 | 0 | 0 | 0 | 0.33 | 0.33 | 2.66 | low |
| Salahuddin et al. 2016 [168] | 0 | 1 | 0 | 0 | 1 | 0 | 0 | 0 | 0.33 | 0.33 | 0.33 | 2.99 | low |
| Sallis et al. 1999 [169] | 1 | 1 | 0 | 0 | 0 | 1 | 1 | 0 | 0 | 0.33 | 0.33 | 4.66 | low |
| Sallis et al. 2002 [170] | 0 | 1 | 0 | 0 | 0 | 1 | 1 | 0 | 0 | 0.33 | 0.33 | 3.66 | low |
| Salmon et al. 2013 [171] | 0 | 1 | 0 | 0 | 0 | 1 | 1 | 0 | 0.33 | 0.33 | 0.33 | 3.99 | low |
| Scheiner et al. 2019 [172] | 0 | 1 | 0 | 1 | 1 | 0 | 0 | 0 | 0 | 0.33 | 0.33 | 3.66 | low |
| Sener et al. 2019 [173] | 0 | 1 | 0 | 0 | 0 | 0 | 0 | 0 | 0 | 0.33 | 0.33 | 1.66 | low |
| Siiba 2021 [174] | 0 | 1 | 1 | 1 | 1 | 0 | 1 | 0 | 0.33 | 0.33 | 0.33 | 5.99 | moderate |
| Silva et al. 2018 [175] | 0 | 1 | 1 | 0 | 1 | 1 | 1 | 0 | 0 | 0.33 | 0.33 | 5.66 | moderate |
| Singh et al. 2008 [176] | 0 | 1 | 0 | 1 | 0 | 1 | 1 | 0 | 0 | 0.33 | 0.33 | 4.66 | low |
| Singh et al. 2009 [177] | 0 | 1 | 0 | 1 | 0 | 1 | 1 | 0 | 0.33 | 0.33 | 0.33 | 4.99 | low |
| Solana et al. 2018 [31] | 0 | 1 | 0 | 1 | 1 | 1 | 0 | 0 | 0 | 0.33 | 0.33 | 4.66 | low |
| Sullivan et al. 2017 [178] | 0 | 1 | 1 | 0 | 1 | 1 | 1 | 0 | 0.33 | 0.33 | 0.33 | 5.99 | moderate |
| Tappe et al. 2013 [179] | 0 | 1 | 1 | 0 | 1 | 1 | 0 | 0 | 0 | 0.33 | 0.33 | 4.66 | low |
| Timperio et al. 2004 [30] | 0 | 1 | 0 | 0 | 0 | 0 | 1 | 0 | 0.33 | 0.33 | 0.33 | 2.99 | low |
| Timperio et al. 2006 [180] | 0 | 1 | 0 | 0 | 0 | 0 | 1 | 0 | 0.33 | 0.33 | 0.33 | 2.99 | low |
| Trapp et al. 2011 [181] | 0 | 1 | 0 | 0 | 0 | 0 | 1 | 0 | 0.33 | 0.33 | 0.33 | 2.99 | low |
| Trapp et al. 2012 [182] | 0 | 1 | 0 | 0 | 0 | 0 | 1 | 0 | 0.33 | 0.33 | 0.33 | 2.99 | low |
| Tung et al. 2016 [183] | 0 | 0.5 | 0 | 0 | 1 | 1 | 0 | 0 | 0 | 0 | 0.33 | 2.83 | low |
| Uys et al. 2016 [184] | 0 | 0.5 | 1 | 0 | 1 | 1 | 1 | 0 | 0.33 | 0 | 0.33 | 5.16 | low |
| Van Kann et al. 2016 [185] | 0 | 1 | 0 | 0 | 0 | 0 | 0 | 0 | 0.33 | 0.33 | 0.33 | 1.99 | low |
| Vanwolleghem et al. 2016 [186] | 0 | 0.5 | 1 | 1 | 1 | 1 | 1 | 0 | 0.33 | 0.33 | 0.33 | 6.49 | moderate |
| Vanwolleghem et al. 2017 [187] | 1 | 1 | 1 | 1 | 1 | 1 | 0 | 0 | 0.33 | 0 | 0.33 | 6.66 | moderate |
| Veitch et al. 2017 [188] | 1 | 0.5 | 0 | 0 | 0 | 0 | 1 | 0 | 0.33 | 0 | 0.33 | 3.16 | low |
| Veugelers et al. 2008 [189] | 0 | 1 | 0 | 0 | 1 | 1 | 1 | 0 | 0.33 | 0.33 | 0.33 | 4.99 | low |
| Villanueva et al. 2013 [190] | 0 | 1 | 0 | 0 | 0 | 0 | 1 | 0 | 0.33 | 0.33 | 0.33 | 2.99 | low |
| Villanueva et al. 2014 [191] | 0 | 1 | 0 | 0 | 0 | 0 | 1 | 0 | 0.33 | 0.33 | 0.33 | 2.99 | low |
| Wang et al. 2022 [192] | 0 | 1 | 1 | 0 | 1 | 0 | 0 | 0 | 0 | 0.33 | 0.33 | 3.66 | low |
| Waygood and Susilo 2015 [193] | 0 | 1 | 0 | 1 | 0 | 0 | 0 | 0 | 0 | 0.33 | 0.33 | 2.66 | low |
| Wex et al. 2023 [194] | 0 | 1 | 0 | 0 | 1 | 0 | 0 | 0 | 0 | 0.33 | 0.33 | 2.66 | low |
| Wilson et al. 2011 [195] | 0 | 0.5 | 0 | 0 | 1 | 1 | 1 | 0 | 0.33 | 0 | 0.33 | 4.16 | low |
| Wilson et al. 2018 [196] | 0 | 1 | 1 | 0 | 1 | 0 | 1 | 0 | 0.33 | 0.33 | 0.33 | 4.99 | low |
| Woldeamanuel 2016 [197] | 0 | 1 | 0 | 1 | 0 | 0 | 1 | 0 | 0 | 0.33 | 0.33 | 3.66 | low |
| Yu and Woo 2017 [77] | 0 | 1 | 0 | 1 | 0 | 1 | 1 | 0 | 0 | 0.33 | 0.33 | 4.66 | low |
| Žaltauskė and Petrauskienė 2016 [198] | 0 | 1 | 0 | 0 | 0 | 0 | 1 | 0 | 0 | 0.33 | 0.33 | 2.66 | low |
| Ziviani et al. 2004 [38] | 0 | 0.5 | 0 | 0 | 0 | 0 | 0 | 0 | 0 | 0 | 0.33 | 0.83 | low |
